# Mortality Associated With Mental Disorders and Comorbid General Medical Conditions

**DOI:** 10.1001/jamapsychiatry.2022.0347

**Published:** 2022-03-30

**Authors:** Natalie C. Momen, Oleguer Plana-Ripoll, Esben Agerbo, Maria K. Christensen, Kim Moesgaard Iburg, Thomas Munk Laursen, Preben B. Mortensen, Carsten B. Pedersen, Anders Prior, Nanna Weye, John J. McGrath

**Affiliations:** 1 National Centre for Register-based Research, School of Business and Social Sciences, Aarhus University, Aarhus, Denmark; 2 Department of Clinical Epidemiology, Aarhus University and Aarhus University Hospital, Aarhus, Denmark; 3 CIRRAU – Centre for Integrated Register-based Research, Aarhus BSS, Aarhus University, Aarhus, Denmark; 4 Department of Public Health, Aarhus University, Aarhus, Denmark; 5 Research Unit for General Practice, Aarhus, Denmark; 6 Queensland Brain Institute, University of Queensland, St Lucia, Queensland, Australia; 7 Queensland Centre for Mental Health Research, The Park Centre for Mental Health, Wacol, Queensland, Australia

## Abstract

**Importance:**

Premature mortality has been observed among people with mental disorders. Comorbid general medical conditions contribute substantially to this reduction in life expectancy.

**Objective:**

To provide an analysis of mortality associated with comorbidity between a broad range of mental disorders and general medical conditions.

**Design, Setting, and Participants:**

Population-based cohort study of 5 946 800 individuals born in Denmark from 1900 to 2015 and residing in the country at the start of follow-up (January 1, 2000, or their date of birth, whichever occurred later).

**Exposures:**

Danish health registers were used to identify people with mental disorders and general medical conditions.

**Main Outcomes and Measures:**

Considering pairs of mental disorders and general medical conditions, we calculated mortality rate ratios (MRRs) and differences in life expectancy (ie, life-years lost) to assess the association of mortality with both disorders of interest compared with the mental disorder of interest, the general medical condition of interest, and neither disorder of interest.

**Results:**

The study population comprised 2 961 397 males and 2 985 403 females, with a median (IQR) age of 32.0 years (7.3-52.9) at start of follow-up and 48.9 years (42.5-68.8) at the end. Based on all pairs of comorbid mental disorders and general medical conditions, the mean MRR compared with people without these conditions was 5.90 (median, 4.94; IQR, 3.80-7.30), and the mean reduction of life expectancy compared with the general population was 11.35 years (median, 11.08; range, 5.27-23.53; IQR, 8.22-13.72). The association with general medical condition comorbidity in those with mental disorders varied by general medical condition; for example, the addition of a neurological condition for each of the mental disorders was associated with a mean MRR of 1.22, whereas for cancer, the mean MRR for all mental disorders was 4.07.

**Conclusions and Relevance:**

In this study, shorter life expectancy was associated with comorbid mental disorders and general medical conditions compared with the entire population and also when compared with patients who had either mental disorders only or general medical conditions only. Prevention and early detection of comorbidities could reduce premature mortality in patients with mental disorders.

## Key Points

**Question:**

How does comorbidity between mental disorders and general medical conditions affect life expectancy?

**Findings:**

In this cohort study of 5 946 800 individuals, those with mental disorder and general medical condition comorbidity had an increased risk of dying and shorter life expectancy compared with the general population, patients with a mental disorder only, and those with general medical conditions only.

**Meaning:**

To reduce premature mortality in those with mental disorders, we need to prevent and actively treat comorbid general medical conditions.

## Teaser

This cohort study examines data from Danish nationwide registries to evaluate how comorbidity between mental disorders and general medical conditions affects life expectancy.

## Introduction

On average, people with mental disorders die earlier than those without them. Our recent study covering the Danish population showed that women and men with a mental disorder die 7 and 10 years earlier, respectively, than the age- and sex-matched general population.^1^ Using state-of-the-art methods, we examined the contribution of different causes of death to the total reduction in life expectancy in people with different mental disorders (eg, mood disorders, schizophrenia, etc). For example, men diagnosed with substance use disorders have a mean life expectancy that is 14.8 years shorter than the general population.^1^ While 5.4 years of this reduction was explained by suicides and unintentional injuries, the remainder (9.4 years) was due to general medical conditions (GMCs), such as diabetes or cardiovascular or respiratory diseases. Other studies focusing on specific mental disorders have also reported that mortality rates from various GMCs are higher among people with mental disorders, although they did not report absolute measures like life expectancy.^2,3,4,5^

The fact that a large part of the mortality gap in those with mental disorders is related to GMCs is not unexpected, given that people with mental disorders have an increased risk of developing comorbid conditions.^6,7^ A recent study reported that people with mental disorders develop GMCs earlier than those without mental disorders and were more likely to die younger than those without a mental disorder or physical disorder.^8^ While it is generally accepted that mental disorder–GMC comorbidity is a key factor underlying premature mortality, there are important gaps in the empirical evidence base. Previous studies have not comprehensively considered a broad range of mental disorder–GMC pairs, making it hard to draw comparisons across pairs, or they have not provided sex-specific estimates. In addition, research has focused on mortality rate ratios (MRRs) or other relative measures, but in recent years, health metrics for assessing premature mortality have developed greatly.^1,9,10,11^ Using both types of measures gives a more thorough picture of the mortality associated with these disorders and their comorbidity.

In our previous study, we quantified MRRs and life-years lost (LYLs) associated with different types of mental disorders, using Danish nationwide-register data.^1^ Here, we extend this investigation by estimating excess mortality associated with pairs of comorbid mental disorders and GMCs. In particular, we are interested in 3 comparisons: we will show MRRs and LYLs for each mental disorder–GMC pair to quantify the association with mortality for the mental disorder–GMC comorbidity compared with (1) people with the mental disorder alone, (2) people with the GMC alone, and (3) those without these disorders or the general population. We present all person- and sex-specific estimates. We prepared an interactive website to facilitate data interrogation (https://nbepi.com/M).

## Methods

### Study Population

This population-based cohort study included all 5 946 800 individuals born in Denmark between 1900 and 2015 and residing there at the start of follow-up (January 1, 2000, or their date of birth, whichever occurred later), identified in the Danish Civil Registration System (2 961 397 males and 2 985 403 females).^12^ The Danish Data Protection Agency and the Danish Health Data Authority approved this study. According to Danish law, informed consent is not required for register-based studies. All data were deidentified and not recognizable at the individual level.

### Ascertainment of Disorders and Mortality

Information on mental disorders was obtained from hospital contact diagnoses (before 1995: inpatient admissions only; 1995 and later: outpatient visits and emergency visits also) recorded in the Danish Psychiatric Central Research Register from January 1969 to December 2016.^13^ Diagnosis dates were defined as the first admission date. To aid comparability with previous publications,^6,14,15^ we focused on 10 broad types of mental disorders. More information about the registers is provided in the eMethods in the Supplement, and details of the specific diagnoses within each mental disorder group are presented in Table 1.

**Table 1.** Mental Disorder Definitions and Frequencies (N = 5 946 800)

Mental disorders	*ICD-10*	*ICD-8* equivalency	Frequency in study population, No. (%)
Organic, including symptomatic, mental disorders	F00-F09	290.09, 290.10, 290.11, 290.18, 290.19, 292.x9, 293.x9, 294.x9, 309.x9	108 821 (1.83)
Mental and behavioral disorders due to psychoactive substance use	F10-F19	291.x9, 294.39, 303.x9, 303.20, 303.28, 303.90, 304.x9	137 031 (2.30)
Schizophrenia and related disorders	F20-F29	295.x9, 296.89, 297.x9, 298.29-298.99, 299.04, 299.05, 299.09, 301.83	87 596 (1.47)
Mood disorders	F30-F39	296.x9 (excluding 296.89), 298.09, 298.19, 300.49, 301.19	227 466 (3.83)
Neurotic, stress-related, and somatoform disorders	F40-F48	300.x9 (excluding 300.49), 305.x9, 305.68, 307.99	294 506 (4.95)
Eating disorders	F50	306.50, 306.58, 306.59	23 570 (0.40)
Specific personality disorders	F60	301.x9 (excluding 301.19), 301.80, 301.81, 301.82, 301.84	128 691 (2.16)
Intellectual disabilities	F70-F79	311.xx, 312.xx, 313.xx, 314.xx, 315.xx	23 124 (0.39)
Pervasive developmental disorders	F84	299.00, 299.01, 299.02, 299.03	33 651 (0.57)
Behavioral and emotional disorders with onset usually occurring in childhood and adolescence	F90-F98	306.x9, 308.0x	86 994 (1.46)

Abbreviations: *ICD-10*, *International Statistical Classification of Diseases and Related Health Problems, Tenth Revision*; *ICD-8*, *International Classification of Diseases, Revision 8*.

Information about GMCs was ascertained from January 1995 until December 2016, using established criteria^6,16^ and combining data on diagnoses made during hospital visits from the Danish National Patient Register^17,18^ and redeemed prescriptions in the Danish National Prescription Register.^19^ Table 2 shows criteria and frequencies for the 9 broad GMC categories and the 31 GMCs they can be divided into. The diagnosis date for the GMC was the admission date for the first hospital diagnosis or date of relevant prescription redemption, whichever occurred first. Date of death was obtained from the Danish Civil Registration System,^12^ which is continuously updated and validated.

**Table 2.** General Medical Conditions: Disorders Within Each Category and Their Frequencies (N = 5 946 800)

Category	Coding definition	Diagnosis codes (*ICD-10*)	Medication		Frequency in study population, No. (%)
			Drug codes (ATC)	Time frame for prescriptions	
Circulatory system					2 091 874 (35.18)
Hypertension	Diagnosis and/or prescription for antihypertensive drugs^a^	I10-I13, I15	C02, C03, C04, C07, C08, C09	Twice in 1 y	1 715 324 (28.84)
Dyslipidemia	Diagnosis and/or prescription for lipid-lowering drugs^b^	E78	C10	Twice in 1 y	718 252 (12.08)
Ischemic heart disease	Diagnosis and/or prescription for antianginal drug	I20-I25	C01DA	Twice in 1 y	496 179 (8.34)
Atrial fibrillation	Diagnosis	I48			317 756 (5.34)
Heart failure	Diagnosis	I50			226 584 (3.81)
Peripheral artery occlusive disease	Diagnosis	I70-I74			200 967 (3.38)
Stroke	Diagnosis	I60-I64, I69			323 376 (5.44)
Endocrine system					701 207 (11.79)
Diabetes	Diagnosis and/or prescription for antidiabetic drugs	E10-E14	A10A, A10B	Twice in 1 y	389 474 (6.55)
Thyroid disorder	Diagnosis and/or prescription for thyroid therapy	E00-E05, E061-E069, E07	H03	Twice in 1 y	325 962 (5.48)
Gout	Diagnosis	E79, M10			45 891 (0.77)
Pulmonary system and allergy					2 065 927 (34.74)
Chronic pulmonary disease	Diagnosis and/or prescription for obstructive airway disease drugs	J40-J47	R03	Twice in 1 y	1 320 124 (22.20)
Allergy	Diagnosis and/or prescription for nonsedative antihistamines and/or nasal antiallergic drugs	J30.1-J30.4, L23, L50.0, T78.0. T78.2, T78.4	R06AX, R06AE07, R06AE09, R01AC, R01AD	Twice in 1 y	1 178 428 (19.82)
Gastrointestinal system					405 284 (6.82)
Ulcer/chronic gastritis	Diagnosis	K221, K25-K28, K293-K295			168 968 (2.84)
Chronic liver disease	Diagnosis	B16-B19, K70, K74, K766, I85			55 708 (0.94)
Inflammatory bowel disease	Diagnosis	K50-K51			66 059 (1.11)
Diverticular disease of intestine	Diagnosis	K57			153 531 (2.58)
Urogenital system					338 356 (5.69)
Chronic kidney disease	Diagnosis	N03, N11, N18-N19			95 761 (1.61)
Prostate disorders	Diagnosis and/or prescription for prostate hyperplasia therapy	N40	C02CA, G04C	Twice in 1 y	264 827 (4.45)
Musculoskeletal system					395 245 (6.65)
Connective tissue disorders	Diagnosis	M05-M06, M08-M09, M30-M36, D86			154 935 (2.61)
Osteoporosis	Diagnosis and/or prescription for osteoporosis drugs	M80-M82	M05B, G03XC01, H05AA	Twice in 1 y	280 383 (4.71)
Painful conditions	Repeated prescriptions of analgesics		N02A, N02BA51, N02BE, M01A, M02A	4× in 1 y	1 833 828 (30.84)
Hematological system					242 914 (4.08)
HIV/AIDS	Diagnosis	B20-B24			4142 (0.07)
Anemias	Diagnosis	D50-D53, D55-D59, D60-D61, D63-D64			239 140 (4.02)
Cancers	Diagnosis	C00-C43, C45-C97			549 081 (9.23)
Neurological system					1 244 857 (20.93)
Vision problems	Diagnosis	H40, H25, H54			448 885 (7.55)
Hearing problems	Diagnosis	H90-H91, H931			422 046 (7.10)
Migraine	Diagnosis and/or prescription for specific antimigraine drugs	G43	N02C	Twice in 1 y	252 834 (4.25)
Epilepsy	Diagnosis and prescription for antiepileptic drugs	G40-G41	N03	Twice in 1 y	77 826 (1.31)
Parkinson disease	Diagnosis	G20-G22			27 458 (0.46)
Multiple sclerosis	Diagnosis	G35			18 757 (0.32)
Neuropathies	Diagnosis	G50-G64			269 995 (4.54)

Abbreviations: ATC, Anatomical Therapeutic Chemical; *ICD-10*, *International Statistical Classification of Diseases and Related Health Problems, Tenth Revision*.

^a^ Ascertained solely by prescriptions only in absence of ischemic heart disease or heart failure and by diuretics only if no kidney disease.

^b^ Prescriptions used if no previous ischemic heart disease.

### Statistical Analysis

Individuals were followed up until the first of the following: date of death, date of emigration, or April 22, 2017 (end of the available data). For each mental disorder–GMC pair (90 for GMC categories, 310 for GMCs), we estimated excess mortality through MRRs and differences in life expectancy (LYLs). For both types of estimates, people with both disorders in the pair of interest were considered exposed, but the comparators differed, as described below. For tractability, we did not consider order of diagnoses.

### Mortality Rate Ratios

**Figure 1.**
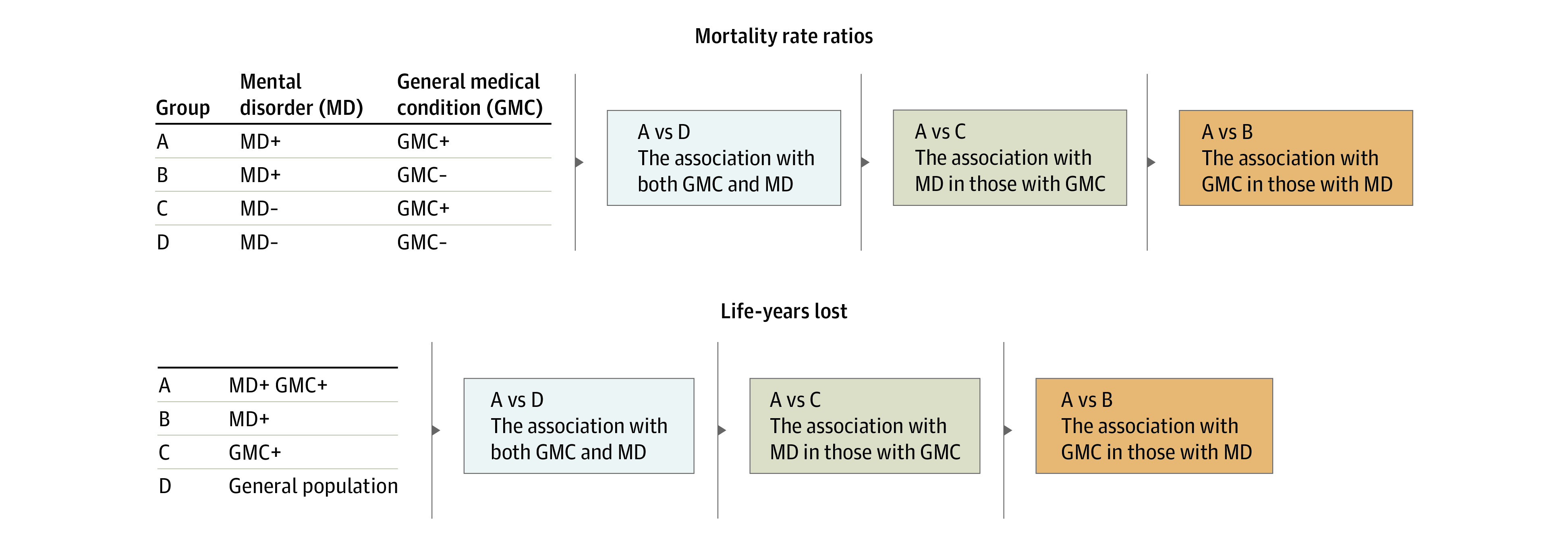
Groups and Comparisons for Mortality Rate Ratios and Life-Years Lost For mortality rate ratios, the general population is divided into 4 discrete groups: mental disorders only (MD+), general medical condition only (GMC+), both (MD+ GMC+), and those without either the mental disorder or the GMC (MD– GMC–). For life-years lost, the general population is divided into 3 overlapping groups. First, the MD+ group comprises everyone with an MD (ie, including those in the MD+ GMC+ group). The GMC+ group comprises everyone with a GMC (ie, including those in the MD+ GMC+ group). Third, the general population comprises everyone (ie, including those in each of the 3 groups MD+, GMC+, and MD+ GMC+ as well as everyone else).

We calculated MRRs using Cox proportional hazards models with age as the underlying time scale, adjusting for sex and calendar time. For each mental disorder–GMC pair, we classified individuals into 4 mutually exclusive categories (Figure 1), depending on whether they were diagnosed with the specific mental disorder or not (MD+ or MD–) and the GMC of interest (GMC+ or GMC–). We then compared the risk of death among people with both disorders of interest (MD+ and GMC+) with the risk of death in each of the other 3 groups. All disorders were treated as time-varying conditions. People with both the mental disorder and GMC of interest were considered exposed to each of these disorders with different onsets depending on the date of first diagnosis for each disorder.

### Life-Years Lost

We calculated differences in mean life expectancy after diagnosis with each mental disorder–GMC pair, compared with people from the general population, people diagnosed with the GMC, and people diagnosed with the mental disorder. These were calculated separately for all persons, men, and women. We previously applied this method, which has been described in detail elsewhere,^9,10,20^ to investigate premature mortality among people with mental disorders.^1,11,21^ The calculation of LYLs is based on the difference in life expectancy between a subcohort of individuals with a particular mental disorder–GMC pair and either (1) the full cohort (ie, a general population sample regardless of mental disorder or GMC status), (2) a subcohort with the mental disorder (regardless of GMC status), or (3) a subcohort with the GMC (regardless of mental disorder status) (Figure 1).

The difference in life expectancy, defined as LYLs, can be interpreted as the mean number of years lost in excess by persons with a specific mental disorder–GMC pair compared with people in each comparator group with the same sex and age.

## Results

The cohort consisted of 5.9 million Danish residents, who were followed up for 86.5 million person-years. At the start of follow-up, median (IQR) age was 32.0 years (7.3-52.9) and at the end, 48.9 years (42.5-68.8). During this time, 901 473 persons died and 94 629 emigrated. Baseline characteristics are presented in eTable 1 in the Supplement. Numbers of cases of each mental disorder diagnosed from 1969 to 2016 are shown in Table 1 and each GMC diagnosed from 1995 to 2016 in Table 2.

**Figure 2.**
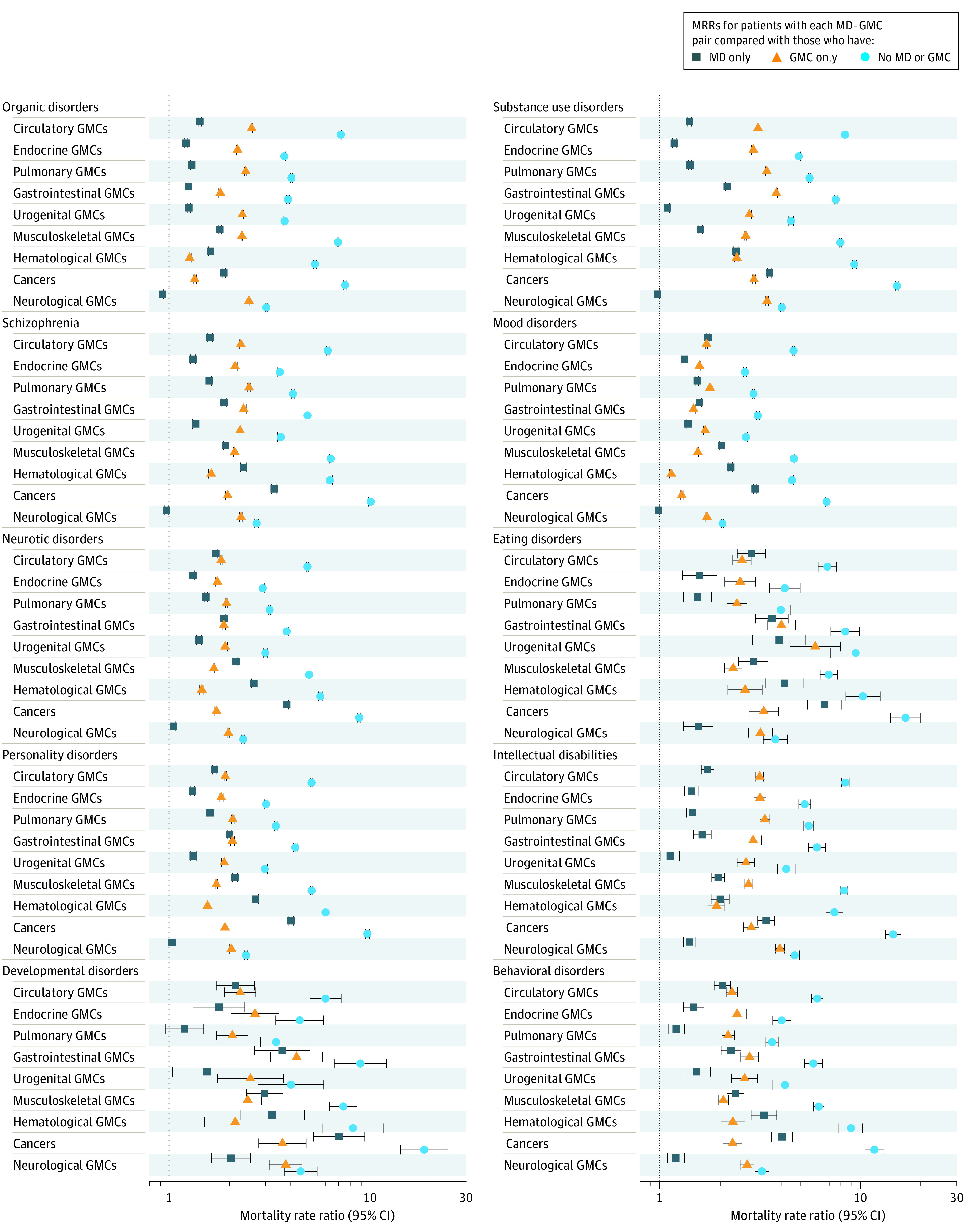
Mortality Rate Ratios (MRRs) for Mental Disorder (MD)–General Medical Condition (GMC) Pairs MRRs are shown for people with a diagnosis of both the MD and GMC of interest compared with people who have no MD or GMC (association with MD-GMC comorbidity), people who have the GMC only (association with MD in people with the GMC), and people who had the MD only (association with GMC in people with the MD). MRRs and 95% CIs are shown on a log scale. Narrow 95% CIs may not be visible. All estimates were adjusted for age, sex, and calendar time.

**Figure 3.**
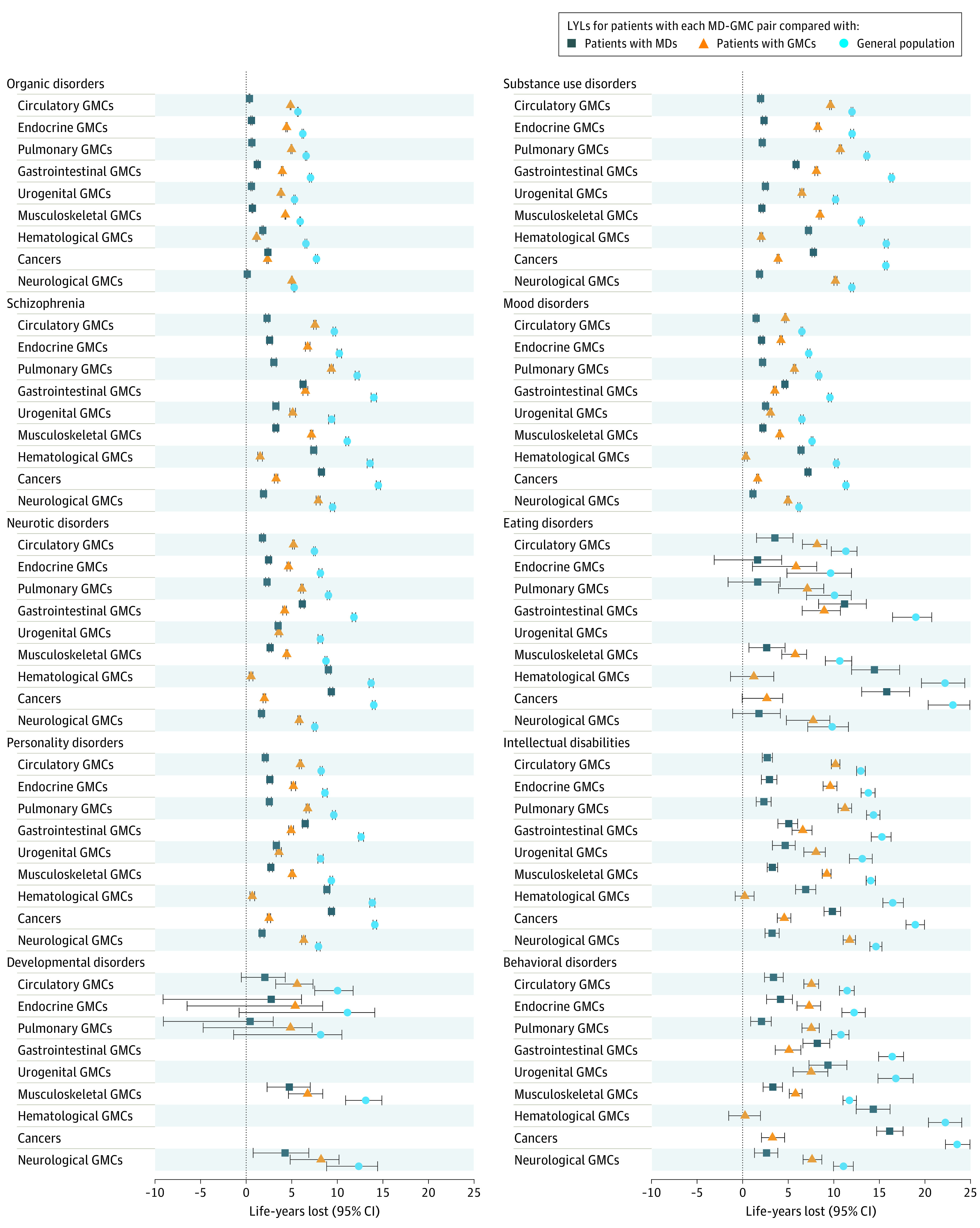
Excess Life-Years Lost (LYLs) for Mental Disorder (MD)–General Medical Condition (GMC) Pairs Life-years lost (the reduction in life expectancy) with 95% CIs are shown for people with a diagnosis of both the MD and GMC of interest compared with those in the general population (association with MD-GMC comorbidity), people with GMCs, regardless of MD status (association with the MD in those with the GMC), and people with MD, regardless of GMC status (association with the GMC in those with the MD). This is calculated for people of the same sex who were alive at ages corresponding to the age-at-onset distribution for those with the MD and GMC. Estimates are not shown where numbers did not meet requirements for reporting of Danish registry data.

The MRRs and LYLs for all persons are displayed in Figure 2 and Figure 3, respectively. For tractability, here we present only results for all persons for pairs of mental disorders and the 9 broad GMC categories and 1 example of a pair including 1 of the 31 GMCs (mood disorders and heart failure) to explain results more fully. The MRRs and LYLs for all persons are shown in eFigures 1A-J in the Supplement; sex-specific MRRs and LYLs are shown in eFigures 2A-J in the Supplement. All estimates can also be viewed in eTable 2 in the Supplement and online (https://nbepi.com/M).

### Mortality Rate Ratios

The MRRs are shown in Figure 2. Overall, mortality rates of people with both disorders of interest (MD+ GMC+) were higher than for those with neither (MD– GMC–) for all 90 mental disorder–GMC category pairs. The mean MRR was 5.90 (median, 4.94; range, 2.05-18.55; IQR, 3.80-7.30).

When we examined association with a mental disorder in addition to a GMC, mortality rates were higher for people with both disorders (MD+ GMC+) compared with those who had only the GMC (MD– GMC+) for all 90 pairs. The mean MRR was 2.40 (median, 2.30; range 1.14-6.11; IQR, 1.90-2.71). The association with mental disorder comorbidity in those with GMCs varied by mental disorder type; for example, the addition of a mood disorder for each GMC resulted in a mean MRR of 1.55, whereas an eating disorder resulted in a mean MRR of 3.23. Mean MRR with the addition of a mental disorder in those with GMCs was 2.61 for males and 2.29 for females (eFigures 2A-J in the Supplement).

When we examined association with a GMC in addition to a mental disorder, mortality rates were higher for people with both disorders of interest (MD+ GMC+) compared with those who had only the mental disorder (MD+ GMC–) for 85 of 90 pairs. The mean MRR was 2.07 (median, 1.66; range, 0.93-7.01; IQR, 1.41-2.32). In 4 pairs, the MRR did not differ for the MD+ GMC+ group compared with the MD+ GMC– group, and for organic disorders and neurological conditions, the MRR indicated lower mortality rates in people with both disorders compared with those who had only organic disorders (MRR, 0.93; 95% CI, 0.91-0.94). The additional mortality associated with GMC comorbidity in people with mental disorders varied by GMC; the addition of a neurological condition to each mental disorder resulted in a mean MRR of 1.22, whereas for cancer, the mean MRR for all mental disorders was 4.07. The addition of a GMC resulted in a mean MRR of 2.14 for males and 2.23 for females (eFigures 2A-J in the Supplement).

For mood disorders and heart failure, the mortality rate for people with both disorders was more than 4 times higher than for people with neither disorder (MRR, 4.45; 95% CI, 4.37-4.54). In addition, MRRs for people with both diagnoses was 1.34 (95% CI, 1.31-1.36), compared with those who had a diagnosis of heart failure only and 2.23 (2.19, 2.28) compared with those who had a diagnosis of mood disorders only. Estimates for the specific 31 GMCs are available in eFigures 1A-J and 2A-J in the Supplement.

### Life-Years Lost

Life-years lost are shown for all persons in eFigures 1A-J in the Supplement. We were unable to present results for all persons for 5 of 90 mental disorder–GMC pairs because of the small numbers of cases. Compared with the entire population of same age and sex (general population), those with both disorders of interest (MD+ GMC+) had a reduced life expectancy for all remaining 85 pairs. Mean number of LYLs was 11.35 years (median, 11.08; range, 5.27-23.53; IQR, 8.22-13.72). People who had both disorders of interest (MD+ GMC+) also had a reduced life expectancy compared with all individuals of the same age with the GMC, regardless of mental disorder status (GMC+) and compared with all individuals who had the mental disorder, regardless of GMC status (MD+), for the 85 pairs. The association with mental disorder comorbidity in people with GMCs was a mean of 5.54 LYLs (median, 5.29; range, 0.23-11.74; IQR, 3.95-7.54), whereas the association with GMC comorbidity in those with mental disorders was a mean of 4.11 LYLs (median, 2.67; range, 0.12-16.13; IQR, 2.06-5.94).

Returning to the example of mood disorders and heart failure, people with both disorders had a reduction in life expectancy of 8.16 years (95% CI, 8.02-8.29) compared with the general population. In addition, the reduction in life expectancy was 1.27 years (95% CI, 1.16-1.39) compared with that for people with a diagnosis of heart failure and 4.95 years (95% CI, 4.82-5.09) compared with those who had a diagnosis of mood disorders (eFigures 1A-J and 2A-J in the Supplement).

## Discussion

This population-based study provides detailed estimates of mortality associated with mental disorder–GMC comorbidity. We believe these estimates provide the most detailed assessments of the association with comorbid GMCs among people who have mental disorders. We want to highlight 3 key findings.

First, MRRs were elevated for all disorder pairs compared with people who had neither disorder; the mean mortality rate for those who had both disorders in the mental disorder–GMC pair of interest compared with people who had neither disorder was almost 6 times higher. Compared with people who had only the mental disorder of interest or only the GMC of interest, it was more than double. Thus, regardless of the mental disorder or GMC type, comorbidity between mental disorders and GMCs is pervasively associated with a substantially increased MRR.

Second, some disorders affect mortality rates more than others. Considering the association with the addition of a GMC (compared with only the mental disorder of interest), the largest mean MRRs were observed for the addition of cancer (4.07) and hematological GMCs (2.67). This is consistent with the general lethality of these disorders; however, regardless of GMC type, MRRs were increased. Considering the association with the addition of a mental disorder (compared with only the GMC of interest), the largest mean MRRs were observed for the addition of eating disorders (3.23) and substance use disorders (3.05). It should be noted that for some combinations of mental disorders and specific GMCs (eg, several mental disorders and dyslipidemia, allergy, migraine, vision problems, and hearing problems), mortality was lower among those with both the GMC and mental disorder, compared with people who had the mental disorder only. While this finding may be due to chance for some pairs, there may be GMC-specific reasons for these observations.

Third, life expectancy was 11.5 years lower in people with comorbid mental disorders and GMCs compared with life expectancy in the general population. The contribution of different GMCs to premature mortality in those with MDs varied but for some pairs was substantial; the reduction in life expectancy ranged between 1.5 months (organic disorders and neurological GMCs) to 16 years (behavioral disorders and cancer). Similarly, the addition of a mental disorder in people with GMCs ranged between 2.5 months (intellectual disorders and hematological GMCs) and 12 years (intellectual disorders and neurological GMCs).

The association between mental disorders and premature mortality is well established.^1,11,22,23^ Earlier articles acknowledge that although some of the premature mortality in people with severe mental disorders could be attributed to external causes (eg, suicide, accidents), a substantial proportion of the premature mortality was attributable to GMC comorbidity, particularly heart disease,^2,4,24^ diabetes,^2,5^ cancer,^3,5,24,25^ and chronic obstructive pulmonary disorder.^2,5^ A study of veterans with type 2 diabetes found that the risk of death rose with increasing GMC comorbidity, regardless of number of psychiatric conditions.^26^

Our article highlights that among people with a mental disorder, the addition of a GMC was generally associated with increased premature mortality; however, the addition of mental disorders in those with GMCs also reduces life expectancy. Although the analyses for the MRRs and LYLs effectively consider the same associations, that is, the association with the addition of a GMC, a mental disorder, or both, the magnitude of results for the 3 comparisons does not always follow the same pattern. It should be remembered that MRRs and LYLs use different comparison groups (Figure 2).

Our study uses the Danish national registers, which provide a large sample size. These data limit recall and self-reporting bias. Because data were available on the entire population and Danish citizens have free and equal access to health care, selection bias is minimized.

### Limitations

There are several important limitations to our study, some of which are covered in more detail in the eDiscussion in the Supplement. First, we restricted GMCs to 9 broad chronic disorder categories and 31 more specific disorders; they did not include accidents and injuries (including self-harm) or acute conditions. We also considered mental disorder–GMC pairs. Future research should consider more specific types of mental disorders and GMCs to examine comorbidity in finer detail, as well as more complex comorbidity, potentially with multiple mental disorders and GMCs (ie, multimorbidity).^26,27^ Combinations of mental disorders and GMCs could be explored, based on analytic methods previously used by our group.^28^ Here, scenarios do not cover the entire complex and transactional pathways that influence mental disorder–GMC temporal patterning (eg, diagnostic overshadowing among those with mental disorders).^29^ Second, it is likely that there is underdetection of both mental disorders and GMCs. Some people will not seek medical advice for conditions. In addition, we have no data from general practitioner visits, although some GMCs were ascertained by prescriptions. It is likely that our study does not detect some of the less severe cases of mental disorders or GMCs, which may lead to overestimation of the associations observed. However, Denmark has free, universal health care, making it likely that disorders are captured in the registry. Third, although mental disorders could be identified in the psychiatric register from 1969 onwards, follow-up for mortality only started in 2000. Thus, for an unknown proportion, we were unable to observe the initial phase after the onset of the mental disorder, when MRRs are higher.^30,31,32^ In addition, we were unable to ascertain GMCs before 1995 due to lack of prescription data. Therefore, for the majority of cases, mental disorder diagnoses will appear first; however, due to the younger age at onset of mental disorders compared with GMCs, this is plausible for many pairs. Furthermore, it is likely that our findings have limited generalizability outside of Denmark: patterns of comorbidity and mortality vary between countries, particularly among those with different health care and socioeconomic structures.

Future research can consider mortality in people with mental disorder–GMC comorbidity in more detail. For many mental disorder–GMC pairs, it may be that neither disorder is the underlying cause of death. A multitude of risk factors contribute to excess mortality in people with mental disorders, such as individual factors, health systems, and social determinants of health.^33^ In our recent article,^1^ we further categorized LYLs for people with mental disorders by cause of death; this could also be carried out for mental disorder–GMC pairs to further add to our knowledge on mortality and comorbidity.

## Conclusions

Our findings highlight that individuals with mental disorder–GMC comorbidity have an increased risk of dying; their life expectancy is shorter than that of both the entire population and people with either mental disorders or GMCs only. Mental-physical multimorbidity is increasing and challenging health care systems globally; it is associated with high health care utilization, costs, and social inequality.^16,27,34,35,36^ Logically, it follows that early identification and good management of mental disorders, as well as prevention of GMC comorbidity, could help reduce some of the risk of premature mortality in people with mental disorders. Prevention and early detection of comorbidity could help reduce the association with mortality, and several studies have demonstrated a reduction in mortality with good mental disorder management.^37,38,39^ However, reviews of interventions to address GMCs and risk behaviors have concluded that health outcomes improve if interventions can be targeted at risk factors such as depression in people with comorbidity.^40^ Although some well-designed interventions appear to be effective at reducing risk factors in people with severe mental disorders, there was low strength of evidence for most interventions.^41^

We hope these estimates provide a foundation for future research aiming to improve life expectancy among people with comorbidity. They highlight the need to optimize screening for GMCs among people with mental disorders so comorbidity can either be prevented or identified early and managed well.

## Notes

Supplement.
eMethods

eDiscussion
**eTable 1.** Characteristics of the study population **eTable 2.** Mortality rate ratios and life-years lost for all pairs **eFigures 1A-1J.** Comparison of mortality rate ratios (MRRs) and life-years lost (LYLs) for pairs of 9 mental disorders and 31 types of general medical conditions within 9 broad categories, all persons **eFigures 2A-2J.** Sex-specific comparison of mortality rate ratios (MRRs) and life-years lost (LYLs) for pairs of 9 mental disorders and 31 types of general medical conditions within 9 broad categories
