# Supplementary material for: Mortality Associated With Mental Disorders and Comorbid General Medical Conditions
Source: JAMA Psychiatry. 2022 Mar 30;79(5):444–53. doi: 10.1001/jamapsychiatry.2022.0347 (PMC8968685; doi:10.1001/jamapsychiatry.2022.0347)
Supplement: Supplement. — eMethods eDiscussion eTable 1. Characteristics of the study population eTable 2. Mortality rate ratios and life-years lost for all pairs eFigures 1A-1J. Comparison of mortality rate ratios (MRRs) and life-years lost (LYLs) for pairs of 9 mental disorders and 31 types of general medical conditions within 9 broad categories, all persons eFigures 2A-2J. Sex-specific comparison of mortality rate ratios (MRRs) and life-years lost (LYLs) for pairs of 9 mental disorders and 31 types of general medical conditions within 9 broad categories [file jamapsychiatry-e220347-s001.pdf]

Supplemental Online Content

Momen NC, Plana-Ripoll O, Agerbo E, et al. Mortality associated with mental disorders and comorbid general medical conditions. *JAMA Psychiatry*. Published online March 30, 2022.
doi:10.1001/jamapsychiatry.2022.0347

eMethods

eDiscussion

eTable 1. Characteristics of the study population

eTable 2. Mortality rate ratios and life-years lost for all pairs

eFigures 1A-1J. Comparison of mortality rate ratios (MRRs) and life-years lost (LYLs) for pairs of 9 mental disorders and 31 types of general medical conditions within 9 broad categories, all persons

eFigures 2A-2J. Sex-specific comparison of mortality rate ratios (MRRs) and life-years lost (LYLs) for pairs of 9 mental disorders and 31 types of general medical conditions within 9 broad categories

This supplemental material has been provided by the authors to give readers additional information about their work.

Aims of the study

In this study, we were interested in quantifying three sets of comparisons. First, we showed MRRs and LYLs for all pairs of mental disorders and GMCs compared to people with neither the mental disorder nor the GMC of interest. This allowed us to quantify the difference in the association of each set of pairwise comorbidity compared to those without these disorders. Second, we will estimate MRRs and LYLs for the same set of pairs of mental disorders and GMCs compared to people with the mental disorder but not the GMC of interest. This allowed us to quantify the difference in the association that is seen when GMCs are diagnosed in addition to the mental disorder of interest. Finally, we estimated MRRs and LYLs for the pairs of mental disorders and GMCs compared to people with the GMC but not the mental disorder of interest; quantifying the difference in the association that is seen when mental disorders are diagnosed in addition to the GMC of interest.

eMethods

Ascertainment of disorders

The diagnoses (ICD-10 codes) and drugs (Anatomical Therapeutic Chemical Classification System codes) used to define each condition are shown in Tables 1 and 2.

Information on mental disorders was obtained from the Danish Psychiatric Central Research Register,[1] which contains details of diagnoses for between 1969 and 2016. This register contains information on psychiatric diagnoses made during hospital contacts (before 1995 only inpatient admissions were included; from 1995 onwards, outpatient visits and emergency visits were also registered). Mental disorder diagnoses were identified between 1969 (when data on psychiatric healthcare contacts are first available) and December 2016. We focused on ten broad types of mental disorders, based on the International Classification of Diseases, Tenth edition, Diagnostic Research Criteria (ICD-10) subchapter F categories (F00-F09, F10-F19, etc.) and corresponding diagnoses in ICD-8. Details of the specific diagnoses within each mental disorder group (and their short labels) are presented in Table 1. Minimum ages of diagnosis varied for each disorder, as in previous studies.[2-4]

Information about GMCs was ascertained from 1995 onwards (the year prescription data became complete) until December 2016, using criteria previously used in Danish comorbidity research.[3,5] Individuals with GMCs were identified by combining data from two sources: 1) diagnoses made during inpatient admissions, and outpatient and emergency visits from the Danish National Patient Register[6,7] and 2) redeemed prescriptions for GMC-specific medications in the Danish National Prescription Register.[8] As for the Danish Psychiatric Central Register, before 1995 only inpatient admissions were included in the Danish National Patient Register, but from 1995 onwards, outpatient visits and emergency visits were also registered. See Table 2 for the criteria for the 31 GMCs, which are grouped into nine GMC categories: circulatory, endocrine, pulmonary, gastrointestinal, urogenital, musculoskeletal, hematological, cancers, and neurological.

© 2022 Momen NC et al. *JAMA Psychiatry*.

Statistical analysis

All mental disorders and GMCs were treated as time-varying conditions. Thus, any individual was considered unexposed until the first diagnosis of a specific disorder (if it occurred); subsequently, the individual was considered to be exposed to the specific mental disorder or GMC. Hence, the person-years and deaths contributed by individuals who were diagnosed before start of follow-up were continually counted as exposed. For individuals who were diagnosed during follow-up, the person-years and deaths were dealt with in the analyses by splitting the follow-up for these individuals into unexposed and exposed parts for each disorder in the pair of interest, and treating the parts as distinct categories with variables that did not change with time.

Mortality rate ratios

To provide an example, of the exposed group and comparator groups for MRRs: when mood disorders were the mental disorder of interest and circulatory conditions were the GMC of interest, we compared risk of death among those with both mood disorders and circulatory conditions (MD+ GMC+), to each of the following: those with mood disorders but not circulatory conditions (MD+ GMC-), those with no mood disorders but with circulatory conditions (MD- GMC+), and those with neither mood disorders nor circulatory conditions (MD- GMC-). When the rates of death among those with and without the mental disorder and GMC of interest are not proportional over time, estimates can be interpreted as an average MRR over the entire follow-up period.[9]

We calculated the mean, median, range and interquartile range (IQR) of MRRs for each pair of disorders.

Life years lost

To provide an example, of the exposed group and comparator groups for life years lost (LYLs): when mood disorders were the mental disorder of interest and circulatory conditions were the general medical condition of interest, we compared risk of death among those with both mood disorders and circulatory conditions (MD+ GMC+), to each of the following: those with mood disorders regardless of circulatory condition status, those with circulatory conditions regardless of mood disorder status and those in the general population sample regardless of mood disorder or circulatory condition status.

Additionally, we calculated the mean, median, range and IQR of LYLs for each pair of disorders.

© 2022 Momen NC et al. *JAMA Psychiatry*.

eDiscussion

Further comments on the limitations of the study

Simplified view of comorbidity

We considered 10 broad categories of mental disorders and nine broad categories of chronic GMCs, as well as looking at GMCs in more detail (31 more specific GMCs). The study did not include accidents and injuries (including self-harm) or acute conditions, e.g. infections, and we did not look at specific diagnoses. We felt this was necessary in order to keep the analyses and their presentation tractable. Future studies should consider comorbidity with additional disorders or injuries.

Further to this, we considered pairs of mental disorders-GMCs. However, many people with comorbidity are diagnosed with multiple disorders. More complex patterns of comorbidity and their associations with mortality should be investigated in future. This could use analytic methods previously implemented by our group to explore combinations of comorbid mental disorders and mortality.[10] This paper is a foundational paper, which aims to give an overview of the association between mental disorder-GMC comorbidity and mortality, but there is further work to be done.

Misclassification of disorders

We expect that there is under-detection of both mental disorders and GMCs in the Danish national registers. This is partly because some people will not seek medical advice regarding conditions. Without a hospital diagnosis or redemption of a relevant prescription, they will not be identified as having a mental disorder or GMC. It is difficult to predict how this may affect our results.

An additional limitation is the lack of diagnosis data from general practitioner visits. While this may be mitigated by the fact that some GMCs were ascertained by prescription redemption, some disorders treated entirely in primary care and without medications included in our criteria will not be identified. It is likely that our study does not detect some of the less severe cases of mental disorders or GMCs, which may lead to an overestimation of the associations observed.

© 2022 Momen NC et al. *JAMA Psychiatry*.

eTable 1. Characteristics of the study population

**Sex**	Male	2,961,397	49.8%
	Female	2,985,403	50.2%
**Birth year**	1900-1910	37,908	0.6%
	1911-1920	192,783	3.2%
	1921-1930	357,663	6.0%
	1931-1940	470,614	7.9%
	1941-1950	709,860	11.9%
	1951-1960	680,633	11.5%
	1961-1970	724,485	12.2%
	1971-1980	636,257	10.7%
	1981-1990	542,310	9.1%
	1991-2000	663,498	11.2%
	2001-2010	642,963	10.8%
	2011-2015	287,826	4.8%
**Age at start of follow-up, median (IQR)**		32.0	(7.3-52.9)
**Age at end of follow-up, median (IQR)**		48.9	(24.5-68.8)

All results are presented in n (%) unless otherwise stated. IQR: Interquartile range

© 2022 Momen NC et al. *JAMA Psychiatry*.

eTable 2. Mortality rate ratios and LYLs for all pairs

Mental disorder	General medical condition	Sex	Compared to those with neither MD nor GMC		Compared to those with GMC only		Compared to those with MD only	
			MRR	LYLs	MRR	LYLs	MRR	LYLs
Organic	Circulatory system	Persons	7.14 (7.07-7.21)	5.67 (5.63-5.72)	2.58 (2.56-2.6)	4.87 (4.83-4.92)	1.42 (1.4-1.45)	0.37 (0.32-0.41)
Substance Use	Circulatory system	Persons	8.35 (8.25-8.45)	12 (11.89-12.1)	3.09 (3.05-3.12)	9.64 (9.53-9.74)	1.41 (1.38-1.44)	1.97 (1.84-2.09)
Schizophrenia	Circulatory system	Persons	6.16 (6.06-6.25)	9.67 (9.53-9.82)	2.28 (2.24-2.31)	7.55 (7.41-7.69)	1.6 (1.56-1.64)	2.3 (2.12-2.48)
Mood	Circulatory system	Persons	4.63 (4.58-4.68)	6.5 (6.41-6.59)	1.71 (1.7-1.73)	4.68 (4.6-4.77)	1.74 (1.71-1.78)	1.48 (1.36-1.58)
Neurotic	Circulatory system	Persons	4.88 (4.82-4.94)	7.49 (7.39-7.59)	1.82 (1.8-1.84)	5.19 (5.09-5.3)	1.71 (1.67-1.75)	1.79 (1.65-1.93)
Eating	Circulatory system	Persons	6.82 (6.14-7.59)	11.32 (9.73-12.55)	2.57 (2.31-2.86)	8.16 (6.57-9.4)	2.86 (2.43-3.36)	3.54 (1.53-5.52)
Personality	Circulatory system	Persons	5.11 (5.04-5.18)	8.23 (8.11-8.36)	1.91 (1.88-1.93)	5.95 (5.83-6.09)	1.69 (1.64-1.73)	2.1 (1.94-2.27)
Intellectual	Circulatory system	Persons	8.37 (8.01-8.75)	12.96 (12.5-13.46)	3.15 (3.01-3.29)	10.19 (9.74-10.68)	1.73 (1.61-1.86)	2.7 (2.15-3.27)
Developmental	Circulatory system	Persons	6 (5.02-7.18)	10.03 (7.52-11.73)	2.26 (1.89-2.7)	5.6 (3.1-7.34)	2.14 (1.72-2.67)	2.05 (-0.53-4.29)
Behavioral	Circulatory system	Persons	6.08 (5.7-6.49)	11.46 (10.62-12.24)	2.29 (2.15-2.44)	7.55 (6.72-8.34)	2.05 (1.87-2.25)	3.38 (2.4-4.45)
Organic	Circulatory system	Males	7.57 (7.47-7.68)	6.38 (6.31-6.46)	2.71 (2.68-2.75)	5.32 (5.25-5.4)	1.47 (1.42-1.51)	0.45 (0.37-0.53)
Substance Use	Circulatory system	Males	9.16 (9.02-9.31)	12.39 (12.26-12.52)	3.28 (3.23-3.33)	9.69 (9.56-9.82)	1.43 (1.39-1.46)	2.14 (1.98-2.3)
Schizophrenia	Circulatory system	Males	6.96 (6.79-7.13)	10.88 (10.67-11.11)	2.52 (2.46-2.58)	8.14 (7.94-8.37)	1.53 (1.48-1.59)	2.6 (2.36-2.89)
Mood	Circulatory system	Males	5.01 (4.93-5.09)	7.31 (7.18-7.45)	1.82 (1.8-1.85)	5.06 (4.93-5.2)	1.65 (1.6-1.7)	1.62 (1.45-1.79)
Neurotic	Circulatory system	Males	5.4 (5.3-5.5)	8.41 (8.24-8.58)	1.97 (1.94-2.01)	5.61 (5.44-5.78)	1.56 (1.51-1.61)	1.93 (1.72-2.15)
Eating	Circulatory system	Males	9.42 (6.79-13.06)	NA	3.49 (2.52-4.83)	NA	2.68 (1.65-4.35)	NA
Personality	Circulatory system	Males	5.62 (5.5-5.74)	9.12 (8.91-9.33)	2.05 (2.01-2.1)	6.4 (6.18-6.61)	1.57 (1.51-1.63)	2.36 (2.1-2.6)
Intellectual	Circulatory system	Males	8.31 (7.81-8.83)	13.1 (12.48-13.79)	3.07 (2.88-3.26)	9.81 (9.21-10.49)	1.87 (1.7-2.06)	3.39 (2.68-4.15)
Developmental	Circulatory system	Males	5.51 (4.43-6.86)	8.88 (5.69-11.05)	2.04 (1.64-2.54)	3.85 (0.72-6.05)	2.06 (1.58-2.67)	2.31 (-1.12-4.81)
Behavioral	Circulatory system	Males	6.77 (6.2-7.4)	12.53 (11.42-13.59)	2.5 (2.29-2.73)	7.81 (6.7-8.89)	2.09 (1.85-2.35)	4.03 (2.63-5.5)
Organic	Circulatory system	Females	6.83 (6.75-6.92)	5.2 (5.14-5.25)	2.49 (2.46-2.51)	4.56 (4.51-4.62)	1.4 (1.36-1.43)	0.31 (0.25-0.37)
Substance Use	Circulatory system	Females	7.39 (7.25-7.52)	11.45 (11.27-11.61)	2.84 (2.8-2.89)	9.57 (9.4-9.74)	1.46 (1.41-1.52)	1.72 (1.51-1.94)
Schizophrenia	Circulatory system	Females	5.64 (5.52-5.75)	8.79 (8.6-8.98)	2.14 (2.1-2.18)	7.12 (6.92-7.31)	1.78 (1.71-1.86)	2.07 (1.84-2.3)
Mood	Circulatory system	Females	4.37 (4.31-4.43)	6.04 (5.93-6.15)	1.66 (1.64-1.67)	4.47 (4.36-4.58)	1.85 (1.8-1.91)	1.39 (1.25-1.53)
Neurotic	Circulatory system	Females	4.52 (4.45-4.59)	6.93 (6.79-7.07)	1.73 (1.71-1.75)	4.94 (4.8-5.08)	1.93 (1.87-1.99)	1.71 (1.53-1.88)

© 2022 Momen NC et al. *JAMA Psychiatry*.

Mental disorder	General medical condition	Sex	Compared to those with neither MD nor GMC		Compared to those with GMC only		Compared to those with MD only	
			MRR	LYLs	MRR	LYLs	MRR	LYLs
Eating	Circulatory system	Females	6.51 (5.82-7.28)	11.14 (9.47-12.43)	2.5 (2.24-2.79)	8.06 (6.4-9.33)	2.89 (2.43-3.43)	3.47 (1.41-5.53)
Personality	Circulatory system	Females	4.73 (4.65-4.82)	7.7 (7.53-7.87)	1.81 (1.78-1.85)	5.68 (5.51-5.85)	1.9 (1.83-1.97)	1.95 (1.73-2.18)
Intellectual	Circulatory system	Females	8.42 (7.91-8.96)	12.81 (12.16-13.5)	3.23 (3.03-3.44)	10.59 (9.93-11.29)	1.52 (1.36-1.7)	1.99 (1.17-2.81)
Developmental	Circulatory system	Females	7.4 (5.45-10.05)	12.21 (8.38-15.2)	2.84 (2.09-3.86)	8.91 (5.04-11.91)	2.15 (1.43-3.22)	1.57 (-2.59-6.26)
Behavioral	Circulatory system	Females	5.42 (4.93-5.96)	10.26 (9.07-11.39)	2.09 (1.9-2.29)	7.25 (6.04-8.38)	2.31 (1.95-2.73)	2.66 (1.23-4.2)
Organic	Hypertension	Persons	4.72 (4.67-4.76)	5.52 (5.47-5.57)	2.52 (2.5-2.54)	4.81 (4.76-4.86)	1.17 (1.15-1.19)	0.35 (0.3-0.4)
Substance Use	Hypertension	Persons	5.82 (5.75-5.89)	11.84 (11.71-11.95)	3.2 (3.16-3.24)	9.57 (9.44-9.69)	1.2 (1.18-1.22)	2.01 (1.87-2.15)
Schizophrenia	Hypertension	Persons	4.18 (4.11-4.25)	9.39 (9.23-9.54)	2.29 (2.25-2.33)	7.41 (7.25-7.56)	1.3 (1.27-1.33)	2.22 (2.04-2.4)
Mood	Hypertension	Persons	3.13 (3.1-3.17)	6.34 (6.25-6.43)	1.71 (1.69-1.73)	4.65 (4.56-4.74)	1.33 (1.31-1.35)	1.45 (1.34-1.55)
Neurotic	Hypertension	Persons	3.41 (3.37-3.45)	7.42 (7.3-7.53)	1.88 (1.86-1.9)	5.27 (5.15-5.39)	1.37 (1.34-1.39)	1.87 (1.73-2.01)
Eating	Hypertension	Persons	5.23 (4.66-5.88)	11.28 (9.42-12.81)	2.9 (2.59-3.26)	8.36 (6.52-9.89)	2.18 (1.86-2.56)	3.53 (1.35-5.76)
Personality	Hypertension	Persons	3.56 (3.51-3.61)	8.14 (7.99-8.28)	1.96 (1.94-1.99)	5.98 (5.84-6.12)	1.36 (1.32-1.39)	2.15 (1.96-2.33)
Intellectual	Hypertension	Persons	5.78 (5.51-6.07)	12.53 (12.07-13.03)	3.2 (3.05-3.36)	9.95 (9.49-10.44)	1.39 (1.29-1.49)	2.41 (1.83-3.01)
Developmental	Hypertension	Persons	4.46 (3.66-5.43)	9.82 (7.02-11.75)	2.47 (2.03-3.01)	5.67 (2.84-7.59)	1.71 (1.35-2.16)	1.74 (-1.14-4.27)
Behavioral	Hypertension	Persons	4.38 (4.07-4.71)	11.38 (10.43-12.28)	2.43 (2.26-2.61)	7.69 (6.73-8.57)	1.52 (1.38-1.67)	3.41 (2.25-4.45)
Organic	Hypertension	Males	5.01 (4.94-5.09)	6.23 (6.15-6.32)	2.65 (2.61-2.68)	5.24 (5.16-5.32)	1.2 (1.17-1.23)	0.48 (0.39-0.56)
Substance Use	Hypertension	Males	6.41 (6.31-6.52)	12.22 (12.06-12.37)	3.43 (3.37-3.48)	9.53 (9.37-9.69)	1.24 (1.21-1.26)	2.25 (2.07-2.43)
Schizophrenia	Hypertension	Males	4.75 (4.62-4.88)	10.63 (10.37-10.89)	2.55 (2.49-2.62)	7.94 (7.67-8.19)	1.27 (1.23-1.32)	2.61 (2.33-2.9)
Mood	Hypertension	Males	3.39 (3.33-3.45)	7.13 (6.97-7.28)	1.82 (1.79-1.85)	4.93 (4.77-5.08)	1.3 (1.27-1.34)	1.63 (1.44-1.8)
Neurotic	Hypertension	Males	3.8 (3.73-3.88)	8.36 (8.17-8.54)	2.05 (2.02-2.09)	5.61 (5.41-5.79)	1.31 (1.27-1.34)	2.09 (1.87-2.3)
Eating	Hypertension	Males	6.93 (4.78-10.04)	NA	3.78 (2.61-5.47)	NA	2 (1.23-3.25)	NA
Personality	Hypertension	Males	3.93 (3.84-4.02)	9.01 (8.77-9.22)	2.12 (2.08-2.17)	6.31 (6.09-6.53)	1.31 (1.27-1.36)	2.47 (2.19-2.73)
Intellectual	Hypertension	Males	5.62 (5.24-6.02)	12.61 (11.92-13.39)	3.06 (2.85-3.27)	9.42 (8.73-10.18)	1.44 (1.31-1.58)	3.11 (2.29-4)
Developmental	Hypertension	Males	3.95 (3.08-5.07)	8.57 (5.49-10.81)	2.15 (1.68-2.76)	3.71 (0.69-5.97)	1.58 (1.19-2.1)	2.05 (-1.21-4.84)
Behavioral	Hypertension	Males	4.95 (4.46-5.49)	12.58 (11.27-13.86)	2.69 (2.43-2.99)	7.9 (6.6-9.16)	1.58 (1.39-1.79)	4.19 (2.65-5.68)
Organic	Hypertension	Females	4.52 (4.46-4.57)	5.08 (5.02-5.14)	2.45 (2.42-2.47)	4.55 (4.49-4.62)	1.17 (1.14-1.19)	0.28 (0.21-0.34)
Substance Use	Hypertension	Females	5.16 (5.07-5.26)	11.35 (11.16-11.54)	2.94 (2.89-2.99)	9.62 (9.42-9.81)	1.22 (1.18-1.26)	1.71 (1.48-1.94)

© 2022 Momen NC et al. *JAMA Psychiatry*.

Mental disorder	General medical condition	Sex	Compared to those with neither MD nor GMC		Compared to those with GMC only		Compared to those with MD only	
			MRR	LYLs	MRR	LYLs	MRR	LYLs
Schizophrenia	Hypertension	Females	3.85 (3.77-3.93)	8.58 (8.39-8.77)	2.17 (2.12-2.21)	7.07 (6.86-7.26)	1.42 (1.37-1.47)	1.98 (1.74-2.2)
Mood	Hypertension	Females	2.97 (2.93-3.01)	5.93 (5.82-6.04)	1.66 (1.64-1.68)	4.51 (4.4-4.63)	1.39 (1.36-1.42)	1.35 (1.22-1.49)
Neurotic	Hypertension	Females	3.16 (3.12-3.21)	6.91 (6.76-7.07)	1.79 (1.77-1.82)	5.09 (4.94-5.24)	1.49 (1.45-1.53)	1.76 (1.57-1.93)
Eating	Hypertension	Females	5.02 (4.45-5.67)	11.16 (9.21-12.81)	2.85 (2.52-3.22)	8.32 (6.38-9.99)	2.22 (1.87-2.63)	3.51 (1.22-5.93)
Personality	Hypertension	Females	3.32 (3.25-3.38)	7.67 (7.48-7.85)	1.88 (1.84-1.91)	5.81 (5.62-6)	1.48 (1.43-1.53)	1.98 (1.74-2.21)
Intellectual	Hypertension	Females	5.92 (5.54-6.34)	12.45 (11.78-13.08)	3.36 (3.14-3.59)	10.45 (9.78-11.07)	1.28 (1.15-1.42)	1.76 (0.94-2.56)
Developmental	Hypertension	Females	5.81 (4.19-8.05)	11.87 (6.65-15.16)	3.29 (2.38-4.57)	8.89 (3.67-12.18)	1.83 (1.21-2.77)	1.23 (-4-5.86)
Behavioral	Hypertension	Females	3.9 (3.52-4.32)	10.21 (8.82-11.43)	2.21 (2-2.45)	7.48 (6.09-8.71)	1.65 (1.41-1.93)	2.65 (1-4.29)
Organic	Dyslipidemia	Persons	3.09 (3.04-3.13)	5.36 (5.28-5.45)	2.79 (2.75-2.84)	5.56 (5.47-5.66)	1.08 (1.06-1.09)	-0.5 (-0.58--0.41)
Substance Use	Dyslipidemia	Persons	3.36 (3.29-3.43)	8.13 (7.86-8.31)	3.02 (2.96-3.09)	7.99 (7.73-8.17)	0.8 (0.78-0.82)	-0.98 (-1.24--0.8)
Schizophrenia	Dyslipidemia	Persons	2.75 (2.67-2.83)	7.48 (7.23-7.75)	2.49 (2.42-2.57)	7.31 (7.06-7.58)	1.05 (1.01-1.08)	0.03 (-0.26-0.31)
Mood	Dyslipidemia	Persons	2.02 (1.99-2.06)	4.67 (4.51-4.79)	1.84 (1.81-1.87)	4.64 (4.48-4.77)	1.04 (1.02-1.06)	-0.31 (-0.48--0.18)
Neurotic	Dyslipidemia	Persons	2.13 (2.09-2.17)	5 (4.81-5.16)	1.94 (1.91-1.98)	4.87 (4.68-5.04)	0.97 (0.95-0.99)	-0.31 (-0.5--0.11)
Eating	Dyslipidemia	Persons	3.91 (3.2-4.78)	8.72 (3.73-11.07)	3.58 (2.93-4.37)	8.14 (3.14-10.49)	1.54 (1.24-1.92)	1.52 (-3.43-4.34)
Personality	Dyslipidemia	Persons	2.13 (2.08-2.19)	5.07 (4.78-5.29)	1.94 (1.89-1.99)	4.96 (4.67-5.19)	0.92 (0.89-0.94)	-0.49 (-0.8--0.25)
Intellectual	Dyslipidemia	Persons	3.8 (3.44-4.2)	8.48 (6.61-10.15)	3.47 (3.14-3.84)	8.08 (6.2-9.75)	1.03 (0.93-1.15)	-1.77 (-3.67--0.05)
Developmental	Dyslipidemia	Persons	2.68 (1.84-3.91)	NA	2.45 (1.68-3.58)	NA	1.04 (0.7-1.54)	NA
Behavioral	Dyslipidemia	Persons	2.79 (2.48-3.14)	8.04 (5.56-9.43)	2.55 (2.27-2.87)	7.33 (4.88-8.73)	1.01 (0.89-1.14)	0.38 (-2.04-1.92)
Organic	Dyslipidemia	Males	3.21 (3.14-3.29)	5.74 (5.62-5.87)	2.85 (2.79-2.92)	5.84 (5.71-5.98)	1.02 (0.99-1.04)	-0.58 (-0.71--0.46)
Substance Use	Dyslipidemia	Males	3.54 (3.45-3.63)	8.42 (8.18-8.67)	3.1 (3.02-3.18)	8.16 (7.91-8.41)	0.75 (0.73-0.78)	-0.94 (-1.21--0.67)
Schizophrenia	Dyslipidemia	Males	2.99 (2.86-3.12)	8.11 (7.68-8.53)	2.66 (2.54-2.78)	7.72 (7.28-8.14)	0.94 (0.9-0.99)	-0.13 (-0.59-0.28)
Mood	Dyslipidemia	Males	2.2 (2.15-2.26)	5.38 (5.16-5.58)	1.97 (1.92-2.02)	5.18 (4.95-5.39)	1.01 (0.98-1.04)	-0.19 (-0.43-0.04)
Neurotic	Dyslipidemia	Males	2.32 (2.26-2.39)	5.66 (5.39-5.91)	2.07 (2.01-2.13)	5.35 (5.09-5.6)	0.89 (0.86-0.92)	-0.36 (-0.66--0.08)
Eating	Dyslipidemia	Males	5.37 (3.12-9.24)	NA	4.83 (2.8-8.31)	NA	1.53 (0.83-2.8)	NA
Personality	Dyslipidemia	Males	2.27 (2.19-2.35)	5.47 (5.01-5.82)	2.02 (1.95-2.1)	5.19 (4.72-5.54)	0.83 (0.8-0.87)	-0.68 (-1.16--0.28)
Intellectual	Dyslipidemia	Males	3.67 (3.19-4.23)	7.02 (3.54-9.95)	3.29 (2.86-3.8)	6.46 (2.99-9.39)	1.04 (0.89-1.2)	-2.85 (-6.32-0.1)
Developmental	Dyslipidemia	Males	2.89 (1.92-4.34)	NA	2.59 (1.72-3.9)	NA	1.21 (0.79-1.86)	NA

© 2022 Momen NC et al. *JAMA Psychiatry*.

Mental disorder	General medical condition	Sex	Compared to those with neither MD nor GMC		Compared to those with GMC only		Compared to those with MD only	
			MRR	LYLs	MRR	LYLs	MRR	LYLs
Behavioral	Dyslipidemia	Males	3.02 (2.58-3.53)	8.94 (5.97-10.38)	2.71 (2.31-3.17)	8.09 (5.12-9.53)	0.97 (0.82-1.15)	0.93 (-2.2-2.56)
Organic	Dyslipidemia	Females	2.98 (2.91-3.04)	5.03 (4.91-5.15)	2.75 (2.69-2.81)	5.32 (5.2-5.44)	1.1 (1.08-1.13)	-0.43 (-0.54--0.32)
Substance Use	Dyslipidemia	Females	3.12 (3.01-3.23)	7.68 (7.14-7.96)	2.89 (2.79-2.99)	7.75 (7.22-8.03)	0.88 (0.84-0.91)	-1.03 (-1.54--0.74)
Schizophrenia	Dyslipidemia	Females	2.57 (2.47-2.68)	6.95 (6.59-7.3)	2.39 (2.29-2.49)	6.96 (6.59-7.33)	1.12 (1.07-1.17)	0.16 (-0.24-0.53)
Mood	Dyslipidemia	Females	1.9 (1.86-1.94)	4.17 (3.94-4.35)	1.77 (1.73-1.81)	4.26 (4.04-4.44)	1.05 (1.02-1.08)	-0.4 (-0.63--0.21)
Neurotic	Dyslipidemia	Females	2 (1.95-2.05)	4.52 (4.27-4.72)	1.86 (1.81-1.91)	4.53 (4.27-4.74)	1.02 (0.99-1.05)	-0.27 (-0.53--0.03)
Eating	Dyslipidemia	Females	3.74 (3.01-4.63)	9.28 (4.38-11.87)	3.49 (2.81-4.32)	8.73 (3.86-11.29)	1.53 (1.21-1.93)	2.28 (-3.02-5.59)
Personality	Dyslipidemia	Females	2.03 (1.96-2.1)	4.8 (4.47-5.07)	1.89 (1.83-1.95)	4.81 (4.47-5.09)	0.98 (0.94-1.01)	-0.35 (-0.72--0.04)
Intellectual	Dyslipidemia	Females	3.94 (3.42-4.54)	10.03 (8.29-11.36)	3.67 (3.18-4.23)	9.8 (8.09-11.14)	1.02 (0.87-1.18)	-0.61 (-2.42-0.87)
Developmental	Dyslipidemia	Females	NA	NA	NA	NA	NA	NA
Behavioral	Dyslipidemia	Females	2.56 (2.15-3.05)	6.8 (3.44-9.58)	2.39 (2-2.84)	6.26 (2.83-9.02)	1.11 (0.91-1.34)	-0.39 (-4.04-2.46)
Organic	Ischemic heart disease	Persons	3.96 (3.91-4.02)	5.47 (5.39-5.54)	2.09 (2.06-2.12)	3.44 (3.37-3.51)	1.22 (1.21-1.24)	0.73 (0.66-0.8)
Substance Use	Ischemic heart disease	Persons	4.7 (4.6-4.8)	10.86 (10.69-11.03)	2.55 (2.5-2.61)	6.48 (6.31-6.65)	1.1 (1.07-1.12)	2.28 (2.1-2.45)
Schizophrenia	Ischemic heart disease	Persons	3.76 (3.66-3.87)	9.4 (9.15-9.63)	2.05 (1.99-2.11)	5.44 (5.21-5.67)	1.35 (1.31-1.4)	3.32 (3.07-3.55)
Mood	Ischemic heart disease	Persons	2.86 (2.82-2.91)	6.65 (6.53-6.77)	1.55 (1.53-1.58)	3.1 (2.98-3.23)	1.41 (1.39-1.44)	2.52 (2.39-2.66)
Neurotic	Ischemic heart disease	Persons	2.94 (2.88-2.99)	7.51 (7.36-7.66)	1.61 (1.58-1.64)	3.11 (2.96-3.27)	1.31 (1.29-1.34)	2.64 (2.47-2.81)
Eating	Ischemic heart disease	Persons	3.67 (2.82-4.77)	NA	2.02 (1.55-2.62)	NA	1.38 (1.05-1.82)	NA
Personality	Ischemic heart disease	Persons	3.07 (3-3.14)	8.05 (7.85-8.25)	1.68 (1.64-1.72)	3.61 (3.4-3.82)	1.31 (1.27-1.34)	2.92 (2.68-3.13)
Intellectual	Ischemic heart disease	Persons	5.47 (4.94-6.04)	13.15 (12.11-14.05)	3 (2.71-3.32)	8.31 (7.31-9.16)	1.42 (1.27-1.58)	4.24 (3.21-5.11)
Developmental	Ischemic heart disease	Persons	4.87 (2.94-8.08)	NA	2.68 (1.61-4.44)	NA	1.85 (1.1-3.11)	NA
Behavioral	Ischemic heart disease	Persons	3.57 (3.17-4.03)	11.74 (10.36-13.14)	1.96 (1.74-2.21)	5.65 (4.29-7.03)	1.26 (1.11-1.44)	4.46 (2.97-5.89)
Organic	Ischemic heart disease	Males	4.21 (4.13-4.3)	5.87 (5.76-5.97)	2.26 (2.21-2.31)	3.86 (3.75-3.97)	1.19 (1.16-1.21)	0.6 (0.49-0.7)
Substance Use	Ischemic heart disease	Males	4.95 (4.83-5.08)	10.93 (10.71-11.13)	2.69 (2.62-2.76)	6.67 (6.45-6.87)	1.04 (1.01-1.07)	1.94 (1.71-2.17)
Schizophrenia	Ischemic heart disease	Males	4.29 (4.11-4.47)	10.42 (10.07-10.79)	2.35 (2.26-2.46)	6.4 (6.08-6.76)	1.29 (1.23-1.36)	3.42 (3.07-3.8)
Mood	Ischemic heart disease	Males	3.06 (2.99-3.14)	7.1 (6.9-7.28)	1.68 (1.64-1.72)	3.64 (3.45-3.83)	1.34 (1.3-1.38)	2.3 (2.09-2.51)
Neurotic	Ischemic heart disease	Males	3.14 (3.06-3.23)	7.78 (7.54-8.01)	1.73 (1.68-1.78)	3.56 (3.32-3.8)	1.18 (1.14-1.22)	2.16 (1.9-2.42)
Eating	Ischemic heart disease	Males	4.19 (2.38-7.38)	NA	2.33 (1.32-4.1)	NA	1.07 (0.57-2.01)	NA

© 2022 Momen NC et al. *JAMA Psychiatry*.

Mental disorder	General medical condition	Sex	Compared to those with neither MD nor GMC		Compared to those with GMC only		Compared to those with MD only	
			MRR	LYLs	MRR	LYLs	MRR	LYLs
Personality	Ischemic heart disease	Males	3.23 (3.13-3.34)	8.34 (8.02-8.63)	1.78 (1.72-1.85)	4.14 (3.82-4.42)	1.18 (1.13-1.23)	2.55 (2.17-2.88)
Intellectual	Ischemic heart disease	Males	5.72 (4.98-6.56)	13.34 (11.87-14.49)	3.17 (2.76-3.64)	8.69 (7.35-9.77)	1.54 (1.33-1.79)	4.63 (3.22-5.82)
Developmental	Ischemic heart disease	Males	4.63 (2.49-8.61)	NA	2.57 (1.38-4.78)	NA	1.88 (1-3.53)	NA
Behavioral	Ischemic heart disease	Males	4.05 (3.45-4.75)	12.42 (10.52-14.26)	2.25 (1.91-2.63)	6.4 (4.53-8.18)	1.29 (1.09-1.53)	4.77 (2.81-6.81)
Organic	Ischemic heart disease	Females	3.77 (3.7-3.84)	5.12 (5.03-5.22)	1.95 (1.91-1.98)	3.08 (2.99-3.17)	1.23 (1.2-1.25)	0.84 (0.75-0.93)
Substance Use	Ischemic heart disease	Females	4.32 (4.18-4.47)	10.74 (10.42-11.03)	2.35 (2.27-2.43)	6.14 (5.82-6.43)	1.18 (1.14-1.23)	2.91 (2.61-3.22)
Schizophrenia	Ischemic heart disease	Females	3.44 (3.32-3.57)	8.55 (8.23-8.86)	1.86 (1.79-1.93)	4.65 (4.34-4.95)	1.41 (1.35-1.46)	3.23 (2.91-3.53)
Mood	Ischemic heart disease	Females	2.73 (2.68-2.79)	6.31 (6.14-6.47)	1.46 (1.44-1.5)	2.69 (2.53-2.85)	1.45 (1.41-1.48)	2.7 (2.52-2.89)
Neurotic	Ischemic heart disease	Females	2.79 (2.72-2.86)	7.28 (7.05-7.49)	1.51 (1.47-1.55)	2.73 (2.49-2.96)	1.4 (1.36-1.43)	3.06 (2.8-3.28)
Eating	Ischemic heart disease	Females	3.56 (2.65-4.78)	NA	1.93 (1.44-2.6)	NA	1.38 (1.01-1.88)	NA
Personality	Ischemic heart disease	Females	2.93 (2.84-3.03)	7.79 (7.5-8.07)	1.59 (1.54-1.64)	3.15 (2.87-3.44)	1.39 (1.35-1.45)	3.23 (2.93-3.53)
Intellectual	Ischemic heart disease	Females	5.21 (4.5-6.03)	12.91 (11.43-14.34)	2.83 (2.44-3.27)	7.83 (6.41-9.15)	1.28 (1.1-1.5)	3.75 (2.27-5.1)
Developmental	Ischemic heart disease	Females	5.41 (2.25-13)	NA	2.94 (1.22-7.06)	NA	1.69 (0.69-4.15)	NA
Behavioral	Ischemic heart disease	Females	3.1 (2.58-3.72)	10.64 (8.34-12.67)	1.69 (1.4-2.02)	4.43 (2.21-6.48)	1.29 (1.06-1.58)	3.97 (1.67-6.14)
Organic	Atrial fibrillation	Persons	4.53 (4.45-4.6)	5.12 (5.05-5.19)	1.89 (1.85-1.92)	2.46 (2.4-2.53)	1.41 (1.39-1.44)	1.15 (1.09-1.21)
Substance Use	Atrial fibrillation	Persons	5.87 (5.7-6.03)	10.99 (10.77-11.21)	2.55 (2.48-2.62)	5.44 (5.25-5.64)	1.42 (1.38-1.46)	4 (3.78-4.21)
Schizophrenia	Atrial fibrillation	Persons	4.43 (4.27-4.6)	8.97 (8.66-9.26)	1.93 (1.86-2)	4.18 (3.92-4.44)	1.61 (1.54-1.67)	4.13 (3.86-4.39)
Mood	Atrial fibrillation	Persons	3.41 (3.34-3.48)	6.6 (6.46-6.75)	1.47 (1.44-1.5)	2.37 (2.24-2.5)	1.68 (1.64-1.72)	3.39 (3.25-3.53)
Neurotic	Atrial fibrillation	Persons	3.69 (3.6-3.78)	8.12 (7.92-8.32)	1.61 (1.57-1.65)	2.73 (2.53-2.92)	1.66 (1.61-1.7)	4.23 (4.03-4.43)
Eating	Atrial fibrillation	Persons	5.17 (3.67-7.27)	NA	2.28 (1.62-3.2)	NA	1.94 (1.37-2.76)	NA
Personality	Atrial fibrillation	Persons	3.81 (3.68-3.93)	8.41 (8.13-8.66)	1.66 (1.61-1.72)	3.01 (2.75-3.25)	1.63 (1.58-1.69)	4.43 (4.16-4.69)
Intellectual	Atrial fibrillation	Persons	6.23 (5.48-7.09)	12.51 (11.32-13.73)	2.74 (2.41-3.12)	6.23 (5.16-7.26)	1.63 (1.43-1.86)	4.85 (3.71-5.94)
Developmental	Atrial fibrillation	Persons	5.02 (2.78-9.07)	NA	2.21 (1.23-4)	NA	1.9 (1.04-3.47)	NA
Behavioral	Atrial fibrillation	Persons	5.35 (4.44-6.45)	NA	2.36 (1.95-2.84)	NA	1.89 (1.56-2.3)	NA
Organic	Atrial fibrillation	Males	4.59 (4.47-4.7)	5.44 (5.33-5.57)	2.01 (1.96-2.06)	2.85 (2.75-2.96)	1.32 (1.28-1.35)	0.99 (0.9-1.09)
Substance Use	Atrial fibrillation	Males	6.13 (5.92-6.35)	11.34 (11.05-11.64)	2.75 (2.65-2.85)	5.84 (5.58-6.11)	1.36 (1.31-1.41)	3.79 (3.5-4.06)
Schizophrenia	Atrial fibrillation	Males	4.91 (4.63-5.2)	10.31 (9.81-10.73)	2.23 (2.1-2.36)	5.3 (4.83-5.68)	1.51 (1.42-1.61)	4.51 (4.06-4.92)

© 2022 Momen NC et al. *JAMA Psychiatry*.

Mental disorder	General medical condition	Sex	Compared to those with neither MD nor GMC		Compared to those with GMC only		Compared to those with MD only	
			MRR	LYLs	MRR	LYLs	MRR	LYLs
Mood	Atrial fibrillation	Males	3.55 (3.44-3.66)	7.22 (7.01-7.47)	1.6 (1.55-1.65)	3 (2.79-3.22)	1.57 (1.52-1.62)	3.36 (3.14-3.6)
Neurotic	Atrial fibrillation	Males	3.88 (3.73-4.03)	8.83 (8.52-9.13)	1.76 (1.69-1.83)	3.51 (3.19-3.81)	1.5 (1.44-1.56)	4.15 (3.85-4.46)
Eating	Atrial fibrillation	Males	4.4 (1.83-10.57)	NA	2.02 (0.84-4.85)	NA	1.13 (0.46-2.82)	NA
Personality	Atrial fibrillation	Males	3.88 (3.69-4.07)	8.7 (8.32-9.11)	1.76 (1.68-1.85)	3.55 (3.18-3.91)	1.46 (1.38-1.53)	4.16 (3.77-4.54)
Intellectual	Atrial fibrillation	Males	6.52 (5.49-7.73)	13.88 (12.25-15.57)	2.99 (2.52-3.55)	7.84 (6.35-9.27)	1.79 (1.5-2.14)	6.31 (4.83-7.78)
Developmental	Atrial fibrillation	Males	5.43 (2.92-10.1)	NA	2.49 (1.34-4.64)	NA	2.23 (1.18-4.19)	NA
Behavioral	Atrial fibrillation	Males	5.36 (4.18-6.87)	NA	2.46 (1.92-3.15)	NA	1.71 (1.32-2.21)	NA
Organic	Atrial fibrillation	Females	4.48 (4.38-4.59)	4.86 (4.76-4.95)	1.77 (1.73-1.81)	2.15 (2.06-2.23)	1.47 (1.44-1.51)	1.28 (1.2-1.36)
Substance Use	Atrial fibrillation	Females	5.46 (5.22-5.72)	10.37 (10.04-10.68)	2.3 (2.19-2.4)	4.72 (4.41-5.02)	1.52 (1.45-1.6)	4.38 (4.06-4.69)
Schizophrenia	Atrial fibrillation	Females	4.15 (3.96-4.36)	7.94 (7.58-8.32)	1.74 (1.66-1.82)	3.33 (3.01-3.67)	1.69 (1.61-1.78)	3.84 (3.5-4.19)
Mood	Atrial fibrillation	Females	3.33 (3.24-3.41)	6.16 (5.98-6.33)	1.37 (1.33-1.41)	1.92 (1.76-2.09)	1.74 (1.69-1.79)	3.41 (3.23-3.59)
Neurotic	Atrial fibrillation	Females	3.56 (3.44-3.67)	7.53 (7.29-7.79)	1.48 (1.43-1.53)	2.09 (1.85-2.32)	1.76 (1.7-1.83)	4.29 (4.04-4.55)
Eating	Atrial fibrillation	Females	5.37 (3.71-7.77)	NA	2.27 (1.56-3.28)	NA	2.08 (1.42-3.04)	NA
Personality	Atrial fibrillation	Females	3.75 (3.59-3.92)	8.17 (7.79-8.49)	1.57 (1.51-1.64)	2.57 (2.19-2.87)	1.77 (1.69-1.86)	4.66 (4.29-4.99)
Intellectual	Atrial fibrillation	Females	5.88 (4.84-7.15)	10.86 (9.2-12.45)	2.48 (2.04-3.02)	4.27 (2.68-5.76)	1.45 (1.19-1.78)	3.09 (1.5-4.63)
Developmental	Atrial fibrillation	Females	NA	NA	NA	NA	NA	NA
Behavioral	Atrial fibrillation	Females	5.32 (4.01-7.06)	NA	2.25 (1.69-2.98)	NA	2.22 (1.66-2.98)	NA
Organic	Heart failure	Persons	5.8 (5.69-5.91)	6.31 (6.22-6.41)	1.66 (1.62-1.69)	1.84 (1.77-1.91)	1.78 (1.74-1.81)	1.96 (1.89-2.03)
Substance Use	Heart failure	Persons	7.43 (7.23-7.64)	12.48 (12.27-12.69)	2.26 (2.19-2.32)	3.05 (2.88-3.22)	1.83 (1.78-1.89)	5.47 (5.28-5.65)
Schizophrenia	Heart failure	Persons	6.17 (5.97-6.38)	11.1 (10.83-11.37)	1.88 (1.81-1.94)	2.62 (2.42-2.81)	2.29 (2.21-2.37)	5.98 (5.74-6.21)
Mood	Heart failure	Persons	4.45 (4.37-4.54)	8.16 (8.02-8.29)	1.34 (1.31-1.36)	1.27 (1.16-1.39)	2.23 (2.19-2.28)	4.95 (4.82-5.09)
Neurotic	Heart failure	Persons	5.01 (4.89-5.13)	10.2 (10.02-10.39)	1.53 (1.49-1.56)	1.38 (1.22-1.54)	2.33 (2.27-2.39)	6.41 (6.24-6.6)
Eating	Heart failure	Persons	5.51 (4.01-7.57)	NA	1.69 (1.23-2.32)	NA	2.11 (1.52-2.94)	NA
Personality	Heart failure	Persons	5.25 (5.1-5.42)	10.7 (10.46-10.95)	1.6 (1.55-1.65)	1.53 (1.32-1.75)	2.32 (2.25-2.4)	6.67 (6.44-6.91)
Intellectual	Heart failure	Persons	8.28 (7.42-9.24)	16.64 (15.49-17.9)	2.53 (2.27-2.83)	4.48 (3.55-5.37)	2.23 (1.98-2.5)	8.43 (7.39-9.55)
Developmental	Heart failure	Persons	9.65 (5.71-16.29)	NA	2.96 (1.75-4.99)	NA	3.75 (2.2-6.41)	NA
Behavioral	Heart failure	Persons	7.25 (6.21-8.47)	NA	2.22 (1.9-2.59)	NA	2.65 (2.25-3.12)	NA

© 2022 Momen NC et al. *JAMA Psychiatry*.

Mental disorder	General medical condition	Sex	Compared to those with neither MD nor GMC		Compared to those with GMC only		Compared to those with MD only	
			MRR	LYLs	MRR	LYLs	MRR	LYLs
Organic	Heart failure	Males	6.21 (6.04-6.38)	6.67 (6.53-6.81)	1.78 (1.73-1.83)	2.16 (2.06-2.27)	1.76 (1.71-1.81)	1.86 (1.76-1.97)
Substance Use	Heart failure	Males	7.9 (7.63-8.17)	12.69 (12.43-12.97)	2.36 (2.28-2.44)	3.41 (3.19-3.65)	1.76 (1.7-1.83)	5.24 (5.02-5.48)
Schizophrenia	Heart failure	Males	7.42 (7.04-7.82)	12.47 (12.05-12.89)	2.25 (2.13-2.37)	3.55 (3.23-3.87)	2.33 (2.2-2.46)	6.46 (6.1-6.83)
Mood	Heart failure	Males	4.89 (4.74-5.04)	8.74 (8.52-8.96)	1.47 (1.42-1.51)	1.75 (1.56-1.94)	2.19 (2.12-2.27)	4.93 (4.72-5.15)
Neurotic	Heart failure	Males	5.21 (5.02-5.4)	10.46 (10.16-10.75)	1.57 (1.52-1.63)	1.67 (1.41-1.92)	2.06 (1.98-2.14)	5.96 (5.67-6.25)
Eating	Heart failure	Males	2.69 (1.12-6.47)	NA	0.82 (0.34-1.97)	NA	0.66 (0.27-1.64)	NA
Personality	Heart failure	Males	5.4 (5.16-5.66)	10.76 (10.42-11.12)	1.64 (1.56-1.71)	1.92 (1.62-2.23)	2.06 (1.97-2.17)	6.19 (5.84-6.55)
Intellectual	Heart failure	Males	9.42 (8.1-10.97)	17.74 (16.14-19.46)	2.87 (2.47-3.34)	5.74 (4.53-6.95)	2.63 (2.24-3.09)	9.69 (8.28-11.21)
Developmental	Heart failure	Males	15.44 (8.77-27.18)	NA	4.7 (2.67-8.28)	NA	6.45 (3.61-11.51)	NA
Behavioral	Heart failure	Males	7.27 (5.9-8.98)	NA	2.22 (1.8-2.73)	NA	2.37 (1.9-2.95)	NA
Organic	Heart failure	Females	5.49 (5.35-5.63)	6.01 (5.89-6.15)	1.56 (1.52-1.6)	1.55 (1.46-1.65)	1.77 (1.72-1.82)	2.04 (1.94-2.14)
Substance Use	Heart failure	Females	6.82 (6.53-7.12)	12.13 (11.8-12.48)	2.1 (2.01-2.2)	2.42 (2.15-2.7)	1.95 (1.87-2.05)	5.87 (5.59-6.15)
Schizophrenia	Heart failure	Females	5.49 (5.25-5.74)	10.02 (9.67-10.36)	1.68 (1.61-1.76)	1.89 (1.64-2.13)	2.3 (2.19-2.41)	5.6 (5.29-5.9)
Mood	Heart failure	Females	4.2 (4.09-4.3)	7.73 (7.56-7.92)	1.26 (1.23-1.3)	0.93 (0.8-1.07)	2.25 (2.19-2.31)	4.97 (4.81-5.14)
Neurotic	Heart failure	Females	4.86 (4.71-5.01)	9.99 (9.75-10.25)	1.49 (1.45-1.54)	1.13 (0.9-1.35)	2.52 (2.43-2.6)	6.79 (6.57-7.05)
Eating	Heart failure	Females	6.54 (4.65-9.2)	NA	2.01 (1.43-2.83)	NA	2.62 (1.84-3.73)	NA
Personality	Heart failure	Females	5.14 (4.93-5.35)	10.64 (10.31-10.99)	1.58 (1.52-1.65)	1.2 (0.93-1.49)	2.53 (2.42-2.64)	7.06 (6.73-7.39)
Intellectual	Heart failure	Females	7.3 (6.22-8.56)	15.46 (13.75-17.12)	2.25 (1.91-2.64)	3.13 (1.81-4.37)	1.87 (1.58-2.22)	7.07 (5.58-8.58)
Developmental	Heart failure	Females	NA	NA	NA	NA	NA	NA
Behavioral	Heart failure	Females	7.22 (5.73-9.1)	NA	2.23 (1.77-2.8)	NA	3.17 (2.48-4.05)	NA
Organic	Peripheral artery occlusive disease	Persons	5.01 (4.9-5.12)	6.77 (6.66-6.89)	1.88 (1.83-1.92)	2.91 (2.81-3)	1.66 (1.62-1.7)	1.65 (1.56-1.74)
Substance Use	Peripheral artery occlusive disease	Persons	5.92 (5.75-6.09)	11.78 (11.55-12)	2.29 (2.23-2.36)	4.17 (3.95-4.38)	1.47 (1.42-1.51)	3.75 (3.53-3.96)
Schizophrenia	Peripheral artery occlusive disease	Persons	4.89 (4.67-5.11)	10.92 (10.53-11.31)	1.9 (1.81-1.98)	3.9 (3.54-4.25)	1.82 (1.74-1.91)	4.92 (4.55-5.28)
Mood	Peripheral artery occlusive disease	Persons	3.75 (3.66-3.84)	8.33 (8.13-8.51)	1.45 (1.41-1.48)	2.15 (1.96-2.32)	1.91 (1.87-1.96)	4.33 (4.14-4.51)

© 2022 Momen NC et al. *JAMA Psychiatry*.

Mental disorder	General medical condition	Sex	Compared to those with neither MD nor GMC		Compared to those with GMC only		Compared to those with MD only	
			MRR	LYLs	MRR	LYLs	MRR	LYLs
Neurotic	Peripheral artery occlusive disease	Persons	4.04 (3.93-4.16)	9.69 (9.44-9.91)	1.57 (1.53-1.62)	2.23 (1.98-2.44)	1.87 (1.82-1.93)	5.13 (4.89-5.35)
Eating	Peripheral artery occlusive disease	Persons	6.36 (4.59-8.82)	NA	2.49 (1.8-3.45)	NA	2.43 (1.74-3.41)	NA
Personality	Peripheral artery occlusive disease	Persons	4.19 (4.04-4.34)	9.97 (9.68-10.29)	1.63 (1.57-1.69)	2.46 (2.18-2.76)	1.84 (1.78-1.91)	5.2 (4.91-5.54)
Intellectual	Peripheral artery occlusive disease	Persons	6.76 (5.74-7.96)	NA	2.64 (2.24-3.11)	NA	1.79 (1.51-2.11)	NA
Developmental	Peripheral artery occlusive disease	Persons	NA	NA	NA	NA	NA	NA
Behavioral	Peripheral artery occlusive disease	Persons	4.93 (4.14-5.87)	15.79 (13.1-18.05)	1.93 (1.62-2.3)	4.82 (2.11-7.06)	1.78 (1.49-2.14)	8.55 (5.84-10.92)
Organic	Peripheral artery occlusive disease	Males	5.15 (4.99-5.31)	7.01 (6.85-7.18)	1.96 (1.9-2.03)	3.14 (3-3.27)	1.56 (1.51-1.62)	1.49 (1.35-1.63)
Substance Use	Peripheral artery occlusive disease	Males	5.94 (5.74-6.16)	11.52 (11.24-11.8)	2.32 (2.24-2.4)	4.05 (3.79-4.31)	1.33 (1.28-1.38)	3.37 (3.1-3.63)
Schizophrenia	Peripheral artery occlusive disease	Males	4.92 (4.61-5.25)	10.84 (10.3-11.39)	1.93 (1.81-2.06)	3.73 (3.22-4.24)	1.54 (1.43-1.64)	4.33 (3.8-4.84)
Mood	Peripheral artery occlusive disease	Males	3.83 (3.7-3.97)	8.26 (8.02-8.52)	1.5 (1.44-1.55)	2.32 (2.09-2.55)	1.74 (1.68-1.81)	3.91 (3.65-4.18)
Neurotic	Peripheral artery occlusive disease	Males	4.14 (3.98-4.31)	9.42 (9.09-9.73)	1.63 (1.56-1.7)	2.26 (1.93-2.55)	1.64 (1.57-1.71)	4.43 (4.09-4.74)
Eating	Peripheral artery occlusive disease	Males	13.89 (5.78-33.38)	NA	5.51 (2.29-13.24)	NA	3.8 (1.53-9.45)	NA
Personality	Peripheral artery occlusive disease	Males	4.26 (4.05-4.47)	9.76 (9.37-10.17)	1.68 (1.6-1.76)	2.57 (2.21-2.96)	1.63 (1.55-1.72)	4.68 (4.25-5.11)
Intellectual	Peripheral artery occlusive disease	Males	7.14 (5.84-8.74)	NA	2.83 (2.31-3.46)	NA	1.97 (1.6-2.43)	NA
Developmental	Peripheral artery occlusive disease	Males	NA	NA	NA	NA	NA	NA
Behavioral	Peripheral artery occlusive disease	Males	5.47 (4.33-6.91)	16.95 (13.54-19.38)	2.17 (1.71-2.74)	5.85 (2.49-8.19)	1.77 (1.39-2.26)	9.46 (5.93-12.01)
Organic	Peripheral artery occlusive disease	Females	4.87 (4.72-5.03)	6.51 (6.35-6.68)	1.78 (1.72-1.84)	2.66 (2.52-2.8)	1.71 (1.65-1.76)	1.82 (1.68-1.95)
Substance Use	Peripheral artery occlusive disease	Females	5.89 (5.59-6.19)	12.31 (11.88-12.71)	2.26 (2.15-2.38)	4.43 (4.01-4.81)	1.69 (1.61-1.78)	4.52 (4.09-4.91)

© 2022 Momen NC et al. *JAMA Psychiatry*.

Mental disorder	General medical condition	Sex	Compared to those with neither MD nor GMC		Compared to those with GMC only		Compared to those with MD only	
			MRR	LYLs	MRR	LYLs	MRR	LYLs
Schizophrenia	Peripheral artery occlusive disease	Females	4.85 (4.56-5.16)	10.99 (10.5-11.51)	1.85 (1.74-1.97)	4.07 (3.6-4.54)	2.05 (1.92-2.18)	5.47 (4.99-5.95)
Mood	Peripheral artery occlusive disease	Females	3.68 (3.57-3.8)	8.38 (8.12-8.63)	1.39 (1.35-1.44)	2 (1.74-2.25)	2 (1.94-2.07)	4.67 (4.41-4.93)
Neurotic	Peripheral artery occlusive disease	Females	3.96 (3.81-4.11)	9.94 (9.58-10.26)	1.51 (1.46-1.57)	2.2 (1.85-2.53)	2.03 (1.95-2.11)	5.74 (5.4-6.06)
Eating	Peripheral artery occlusive disease	Females	5.86 (4.12-8.33)	NA	2.26 (1.59-3.21)	NA	2.31 (1.61-3.33)	NA
Personality	Peripheral artery occlusive disease	Females	4.12 (3.92-4.32)	10.16 (9.72-10.61)	1.58 (1.5-1.66)	2.35 (1.95-2.78)	2 (1.9-2.11)	5.68 (5.26-6.11)
Intellectual	Peripheral artery occlusive disease	Females	6.13 (4.65-8.09)	NA	2.36 (1.79-3.11)	NA	1.54 (1.16-2.04)	NA
Developmental	Peripheral artery occlusive disease	Females	NA	NA	NA	NA	NA	NA
Behavioral	Peripheral artery occlusive disease	Females	4.39 (3.38-5.71)	14.38 (10.32-18.23)	1.69 (1.3-2.2)	3.59 (-0.57-7.42)	1.88 (1.43-2.48)	7.46 (3.56-11.5)
Organic	Stroke	Persons	4.82 (4.76-4.89)	6.66 (6.58-6.74)	1.7 (1.67-1.72)	2.38 (2.32-2.45)	1.55 (1.52-1.57)	1.27 (1.2-1.35)
Substance Use	Stroke	Persons	6.39 (6.26-6.53)	12.75 (12.56-12.92)	2.32 (2.27-2.37)	4.37 (4.19-4.53)	1.57 (1.53-1.61)	4.48 (4.29-4.66)
Schizophrenia	Stroke	Persons	4.97 (4.83-5.12)	10.73 (10.48-11)	1.8 (1.74-1.85)	3.41 (3.18-3.64)	1.82 (1.76-1.88)	4.95 (4.7-5.21)
Mood	Stroke	Persons	3.83 (3.77-3.9)	8.11 (7.97-8.24)	1.37 (1.35-1.4)	1.73 (1.6-1.85)	1.93 (1.89-1.97)	4.29 (4.15-4.43)
Neurotic	Stroke	Persons	4.19 (4.11-4.28)	10.01 (9.82-10.2)	1.52 (1.48-1.55)	1.76 (1.58-1.95)	1.93 (1.88-1.97)	5.44 (5.25-5.63)
Eating	Stroke	Persons	7.66 (6.01-9.77)	NA	2.79 (2.19-3.56)	NA	2.99 (2.31-3.87)	NA
Personality	Stroke	Persons	4.48 (4.36-4.6)	10.44 (10.21-10.68)	1.62 (1.58-1.67)	2.31 (2.07-2.55)	1.96 (1.91-2.02)	5.69 (5.45-5.94)
Intellectual	Stroke	Persons	6.72 (6.1-7.39)	15.09 (14.03-16.06)	2.44 (2.22-2.69)	5.08 (4.06-5.96)	1.77 (1.6-1.96)	6.15 (5.09-7.09)
Developmental	Stroke	Persons	8.58 (6-12.28)	NA	3.12 (2.18-4.47)	NA	3.38 (2.32-4.91)	NA
Behavioral	Stroke	Persons	5.9 (5.18-6.72)	17.72 (15.96-19.3)	2.15 (1.88-2.45)	4.01 (2.19-5.57)	2.15 (1.87-2.47)	10.3 (8.35-12.06)
Organic	Stroke	Males	5 (4.91-5.1)	7.1 (6.98-7.22)	1.84 (1.81-1.88)	2.79 (2.7-2.89)	1.5 (1.46-1.53)	1.17 (1.06-1.28)
Substance Use	Stroke	Males	6.74 (6.56-6.93)	12.85 (12.63-13.08)	2.51 (2.44-2.58)	4.59 (4.38-4.81)	1.51 (1.47-1.56)	4.25 (4.02-4.46)
Schizophrenia	Stroke	Males	5.47 (5.22-5.73)	11.78 (11.39-12.18)	2.04 (1.94-2.13)	4.09 (3.74-4.48)	1.7 (1.61-1.78)	5.13 (4.73-5.52)
Mood	Stroke	Males	4.05 (3.94-4.16)	8.54 (8.33-8.75)	1.5 (1.46-1.55)	2.22 (2.02-2.42)	1.84 (1.78-1.89)	4.12 (3.91-4.33)
Neurotic	Stroke	Males	4.5 (4.36-4.65)	10.34 (10.08-10.62)	1.68 (1.62-1.73)	2.26 (2-2.54)	1.77 (1.71-1.84)	5.05 (4.77-5.36)

© 2022 Momen NC et al. *JAMA Psychiatry*.

Mental disorder	General medical condition	Sex	Compared to those with neither MD nor GMC		Compared to those with GMC only		Compared to those with MD only	
			MRR	LYLs	MRR	LYLs	MRR	LYLs
Eating	Stroke	Males	NA	NA	NA	NA	NA	NA
Personality	Stroke	Males	4.76 (4.57-4.95)	10.82 (10.5-11.18)	1.78 (1.71-1.85)	2.92 (2.61-3.25)	1.83 (1.75-1.91)	5.48 (5.13-5.84)
Intellectual	Stroke	Males	6.74 (5.91-7.69)	15.64 (13.93-17.13)	2.53 (2.22-2.89)	5.62 (3.97-6.84)	1.86 (1.62-2.15)	6.92 (5.19-8.26)
Developmental	Stroke	Males	9.45 (6.16-14.49)	NA	3.55 (2.31-5.44)	NA	3.99 (2.56-6.24)	NA
Behavioral	Stroke	Males	7.2 (6.03-8.59)	19.39 (16.94-21.63)	2.7 (2.26-3.23)	5.18 (2.72-7.43)	2.37 (1.96-2.86)	11.44 (8.84-13.87)
Organic	Stroke	Females	4.68 (4.59-4.76)	6.27 (6.17-6.37)	1.57 (1.54-1.6)	2.01 (1.92-2.1)	1.56 (1.53-1.59)	1.37 (1.27-1.46)
Substance Use	Stroke	Females	5.88 (5.68-6.09)	12.58 (12.26-12.89)	2.08 (2.01-2.15)	4 (3.69-4.28)	1.66 (1.6-1.73)	4.86 (4.55-5.16)
Schizophrenia	Stroke	Females	4.68 (4.5-4.86)	9.92 (9.58-10.24)	1.64 (1.58-1.71)	2.87 (2.59-3.15)	1.93 (1.85-2.02)	4.81 (4.49-5.12)
Mood	Stroke	Females	3.7 (3.62-3.78)	7.81 (7.63-8)	1.28 (1.25-1.31)	1.38 (1.2-1.57)	1.98 (1.93-2.03)	4.4 (4.23-4.6)
Neurotic	Stroke	Females	4 (3.89-4.11)	9.77 (9.53-10.01)	1.4 (1.36-1.44)	1.4 (1.15-1.64)	2.03 (1.97-2.09)	5.72 (5.48-5.98)
Eating	Stroke	Females	7.69 (5.99-9.89)	NA	2.71 (2.11-3.49)	NA	3.11 (2.38-4.06)	NA
Personality	Stroke	Females	4.27 (4.12-4.43)	10.15 (9.83-10.45)	1.5 (1.45-1.56)	1.83 (1.51-2.12)	2.06 (1.98-2.15)	5.85 (5.52-6.18)
Intellectual	Stroke	Females	6.68 (5.81-7.68)	14.46 (13.22-15.72)	2.35 (2.05-2.71)	4.46 (3.37-5.69)	1.67 (1.44-1.94)	5.28 (4.04-6.56)
Developmental	Stroke	Females	7.04 (3.66-13.53)	NA	2.48 (1.29-4.77)	NA	2.23 (1.12-4.43)	NA
Behavioral	Stroke	Females	4.88 (4.02-5.91)	15.41 (12.8-17.9)	1.72 (1.42-2.08)	2.38 (-0.24-4.73)	2.1 (1.7-2.59)	8.71 (5.97-11.24)
Organic	Endocrine system	Persons	3.74 (3.69-3.79)	6.22 (6.13-6.31)	2.19 (2.16-2.22)	4.44 (4.36-4.53)	1.21 (1.2-1.23)	0.58 (0.5-0.66)
Substance Use	Endocrine system	Persons	4.91 (4.82-5.01)	12.01 (11.84-12.18)	2.93 (2.87-2.98)	8.24 (8.06-8.42)	1.18 (1.16-1.21)	2.35 (2.17-2.53)
Schizophrenia	Endocrine system	Persons	3.56 (3.47-3.65)	10.22 (9.98-10.47)	2.13 (2.08-2.18)	6.75 (6.5-7)	1.32 (1.28-1.36)	2.57 (2.33-2.85)
Mood	Endocrine system	Persons	2.66 (2.62-2.7)	7.25 (7.11-7.39)	1.58 (1.55-1.6)	4.21 (4.06-4.37)	1.33 (1.31-1.35)	2.07 (1.92-2.24)
Neurotic	Endocrine system	Persons	2.92 (2.87-2.97)	8.14 (7.97-8.33)	1.74 (1.71-1.77)	4.65 (4.48-4.84)	1.32 (1.29-1.34)	2.46 (2.28-2.68)
Eating	Endocrine system	Persons	4.19 (3.52-4.99)	9.65 (4.86-11.95)	2.51 (2.11-2.99)	5.86 (1.08-8.13)	1.58 (1.3-1.93)	1.64 (-3.12-4.28)
Personality	Endocrine system	Persons	3.04 (2.97-3.11)	8.64 (8.4-8.89)	1.82 (1.78-1.86)	5.18 (4.95-5.43)	1.31 (1.28-1.34)	2.6 (2.32-2.86)
Intellectual	Endocrine system	Persons	5.27 (4.92-5.64)	13.79 (13-14.53)	3.16 (2.95-3.38)	9.61 (8.82-10.35)	1.44 (1.33-1.56)	2.94 (2.06-3.76)
Developmental	Endocrine system	Persons	4.47 (3.39-5.88)	11.12 (-0.77-14.11)	2.68 (2.03-3.52)	5.38 (-6.48-8.39)	1.77 (1.32-2.38)	2.75 (-9.11-6.08)
Behavioral	Endocrine system	Persons	4.05 (3.65-4.5)	12.22 (10.89-13.45)	2.43 (2.19-2.69)	7.3 (5.96-8.56)	1.48 (1.32-1.66)	4.15 (2.62-5.46)
Organic	Endocrine system	Males	4.2 (4.1-4.3)	7.03 (6.88-7.18)	2.23 (2.18-2.29)	4.68 (4.55-4.82)	1.26 (1.23-1.29)	0.73 (0.61-0.86)

© 2022 Momen NC et al. *JAMA Psychiatry*.

Mental disorder	General medical condition	Sex	Compared to those with neither MD nor GMC		Compared to those with GMC only		Compared to those with MD only	
			MRR	LYLs	MRR	LYLs	MRR	LYLs
Substance Use	Endocrine system	Males	5.51 (5.37-5.66)	12.4 (12.17-12.61)	2.96 (2.88-3.04)	7.8 (7.55-8.02)	1.21 (1.17-1.24)	2.55 (2.3-2.78)
Schizophrenia	Endocrine system	Males	4.28 (4.11-4.45)	11.44 (11.06-11.85)	2.32 (2.23-2.41)	6.66 (6.29-7.06)	1.34 (1.28-1.4)	3.05 (2.65-3.49)
Mood	Endocrine system	Males	3.19 (3.11-3.27)	8.41 (8.17-8.65)	1.72 (1.68-1.77)	4.34 (4.1-4.57)	1.45 (1.41-1.49)	2.73 (2.47-2.98)
Neurotic	Endocrine system	Males	3.6 (3.49-3.71)	9.56 (9.25-9.84)	1.95 (1.89-2.01)	4.84 (4.54-5.13)	1.41 (1.37-1.46)	3.22 (2.9-3.52)
Eating	Endocrine system	Males	5 (2.96-8.44)	NA	2.72 (1.61-4.59)	NA	1.33 (0.73-2.39)	NA
Personality	Endocrine system	Males	3.61 (3.48-3.74)	9.72 (9.35-10.05)	1.96 (1.89-2.03)	5.1 (4.72-5.44)	1.37 (1.32-1.43)	3.21 (2.81-3.57)
Intellectual	Endocrine system	Males	5.28 (4.76-5.86)	13.57 (12.28-14.56)	2.87 (2.59-3.19)	8.05 (6.8-9.03)	1.48 (1.32-1.67)	3.49 (2.18-4.58)
Developmental	Endocrine system	Males	4.9 (3.53-6.79)	13.41 (-3.71-16.27)	2.66 (1.92-3.69)	6.25 (-10.82-9.22)	2.08 (1.47-2.96)	6.78 (-10.25-9.83)
Behavioral	Endocrine system	Males	4.99 (4.33-5.75)	13.83 (11.73-15.54)	2.71 (2.35-3.13)	7.09 (5.01-8.78)	1.65 (1.41-1.93)	5.3 (3.04-7.3)
Organic	Endocrine system	Females	3.47 (3.41-3.53)	5.74 (5.64-5.85)	2.21 (2.17-2.25)	4.3 (4.2-4.41)	1.2 (1.18-1.23)	0.5 (0.39-0.59)
Substance Use	Endocrine system	Females	4.3 (4.17-4.43)	11.54 (11.24-11.82)	2.79 (2.7-2.87)	8.77 (8.47-9.05)	1.2 (1.16-1.24)	2.12 (1.8-2.43)
Schizophrenia	Endocrine system	Females	3.18 (3.09-3.28)	9.49 (9.19-9.82)	2.07 (2.01-2.13)	6.8 (6.49-7.13)	1.36 (1.31-1.41)	2.3 (1.96-2.63)
Mood	Endocrine system	Females	2.4 (2.36-2.45)	6.74 (6.56-6.92)	1.55 (1.52-1.58)	4.15 (3.95-4.34)	1.3 (1.27-1.32)	1.78 (1.57-1.99)
Neurotic	Endocrine system	Females	2.58 (2.52-2.64)	7.51 (7.3-7.74)	1.67 (1.63-1.71)	4.56 (4.35-4.81)	1.29 (1.26-1.33)	2.12 (1.89-2.39)
Eating	Endocrine system	Females	4.02 (3.34-4.84)	9.59 (4.59-11.94)	2.61 (2.17-3.15)	5.93 (0.84-8.24)	1.61 (1.31-1.99)	1.67 (-3.32-4.57)
Personality	Endocrine system	Females	2.73 (2.65-2.8)	8.16 (7.87-8.45)	1.77 (1.72-1.82)	5.22 (4.94-5.52)	1.31 (1.27-1.36)	2.33 (1.99-2.65)
Intellectual	Endocrine system	Females	5.23 (4.77-5.74)	13.95 (12.93-15.01)	3.4 (3.1-3.73)	10.77 (9.75-11.86)	1.36 (1.22-1.52)	2.53 (1.4-3.69)
Developmental	Endocrine system	Females	3.73 (2.25-6.19)	8.01 (-8.73-14.89)	2.42 (1.46-4.02)	4.2 (-12.46-11.11)	1.13 (0.65-1.95)	-2.7 (-18.76-5.15)
Behavioral	Endocrine system	Females	3.32 (2.85-3.86)	11.05 (9.17-12.76)	2.15 (1.85-2.51)	7.46 (5.58-9.21)	1.43 (1.2-1.71)	3.31 (1.24-5.29)
Organic	Diabetes mellitus	Persons	3.95 (3.88-4.02)	6.73 (6.62-6.84)	2.05 (2.01-2.09)	4.17 (4.07-4.28)	1.32 (1.29-1.35)	0.88 (0.78-0.98)
Substance Use	Diabetes mellitus	Persons	5.55 (5.42-5.68)	12.98 (12.78-13.2)	2.94 (2.87-3.01)	7.75 (7.54-7.95)	1.38 (1.35-1.42)	3.42 (3.2-3.63)
Schizophrenia	Diabetes mellitus	Persons	4.01 (3.89-4.13)	11.43 (11.15-11.72)	2.13 (2.07-2.19)	6.14 (5.87-6.42)	1.53 (1.48-1.58)	3.77 (3.48-4.08)
Mood	Diabetes mellitus	Persons	3.09 (3.03-3.15)	8.83 (8.65-9.01)	1.63 (1.6-1.66)	4.09 (3.91-4.28)	1.59 (1.56-1.62)	3.67 (3.48-3.86)
Neurotic	Diabetes mellitus	Persons	3.42 (3.34-3.5)	9.87 (9.64-10.09)	1.82 (1.78-1.86)	4.37 (4.13-4.6)	1.6 (1.56-1.63)	4.22 (3.98-4.45)
Eating	Diabetes mellitus	Persons	5.07 (4.06-6.33)	11.59 (2.99-14.88)	2.7 (2.16-3.37)	3.95 (-4.56-7.27)	1.97 (1.55-2.5)	3.55 (-5.14-7.03)
Personality	Diabetes mellitus	Persons	3.56 (3.46-3.65)	10.29 (10.01-10.55)	1.89 (1.84-1.94)	4.82 (4.54-5.1)	1.58 (1.54-1.63)	4.32 (4.02-4.59)
Intellectual	Diabetes mellitus	Persons	5.08 (4.66-5.54)	13.27 (12.34-14.12)	2.7 (2.48-2.95)	7.18 (6.26-8.01)	1.37 (1.24-1.5)	2.83 (1.91-3.72)

© 2022 Momen NC et al. *JAMA Psychiatry*.

Mental disorder	General medical condition	Sex	Compared to those with neither MD nor GMC		Compared to those with GMC only		Compared to those with MD only	
			MRR	LYLs	MRR	LYLs	MRR	LYLs
Developmental	Diabetes mellitus	Persons	4.6 (3.25-6.5)	NA	2.44 (1.73-3.45)	NA	1.79 (1.25-2.57)	NA
Behavioral	Diabetes mellitus	Persons	5.01 (4.43-5.66)	15.02 (13.49-16.54)	2.66 (2.36-3.01)	7.52 (5.99-9.01)	1.87 (1.64-2.13)	6.95 (5.17-8.52)
Organic	Diabetes mellitus	Males	4.22 (4.11-4.33)	7.25 (7.1-7.42)	2.16 (2.1-2.22)	4.6 (4.45-4.75)	1.29 (1.25-1.32)	0.85 (0.7-0.98)
Substance Use	Diabetes mellitus	Males	5.78 (5.61-5.94)	12.88 (12.6-13.14)	3 (2.91-3.09)	7.64 (7.36-7.91)	1.29 (1.25-1.33)	3.09 (2.82-3.38)
Schizophrenia	Diabetes mellitus	Males	4.44 (4.25-4.64)	11.8 (11.39-12.18)	2.32 (2.22-2.43)	6.32 (5.93-6.72)	1.41 (1.35-1.48)	3.45 (3.05-3.85)
Mood	Diabetes mellitus	Males	3.4 (3.3-3.5)	9.02 (8.75-9.27)	1.77 (1.72-1.82)	4.39 (4.12-4.64)	1.56 (1.51-1.62)	3.35 (3.08-3.61)
Neurotic	Diabetes mellitus	Males	3.75 (3.63-3.87)	9.98 (9.68-10.29)	1.96 (1.9-2.03)	4.63 (4.32-4.94)	1.49 (1.44-1.55)	3.72 (3.41-4.04)
Eating	Diabetes mellitus	Males	3.92 (2.04-7.54)	NA	2.06 (1.07-3.95)	NA	1 (0.5-2.02)	NA
Personality	Diabetes mellitus	Males	3.85 (3.7-4.01)	10.32 (9.93-10.66)	2.02 (1.94-2.1)	5.08 (4.71-5.43)	1.49 (1.43-1.56)	3.89 (3.49-4.27)
Intellectual	Diabetes mellitus	Males	4.78 (4.2-5.43)	12.57 (11.23-13.86)	2.5 (2.2-2.84)	6.43 (5.08-7.68)	1.32 (1.15-1.51)	2.71 (1.37-4.06)
Developmental	Diabetes mellitus	Males	4.85 (3.22-7.29)	NA	2.54 (1.69-3.82)	NA	2.03 (1.32-3.11)	NA
Behavioral	Diabetes mellitus	Males	5.34 (4.55-6.28)	14.9 (12.77-16.78)	2.8 (2.39-3.29)	7.22 (5.03-9.12)	1.77 (1.49-2.11)	6.46 (4.07-8.49)
Organic	Diabetes mellitus	Females	3.74 (3.65-3.83)	6.26 (6.1-6.42)	1.96 (1.91-2.01)	3.79 (3.65-3.94)	1.32 (1.29-1.36)	0.91 (0.77-1.04)
Substance Use	Diabetes mellitus	Females	5.22 (5.02-5.42)	13.17 (12.77-13.54)	2.83 (2.72-2.94)	7.95 (7.59-8.32)	1.52 (1.46-1.58)	3.97 (3.59-4.33)
Schizophrenia	Diabetes mellitus	Females	3.69 (3.54-3.84)	11.12 (10.71-11.52)	2 (1.92-2.08)	5.98 (5.59-6.37)	1.6 (1.53-1.67)	4.04 (3.63-4.45)
Mood	Diabetes mellitus	Females	2.88 (2.8-2.95)	8.7 (8.44-8.97)	1.54 (1.5-1.59)	3.87 (3.61-4.14)	1.58 (1.54-1.63)	3.89 (3.62-4.16)
Neurotic	Diabetes mellitus	Females	3.17 (3.08-3.27)	9.79 (9.47-10.1)	1.72 (1.66-1.77)	4.18 (3.85-4.52)	1.64 (1.59-1.7)	4.58 (4.24-4.91)
Eating	Diabetes mellitus	Females	5.26 (4.16-6.67)	11.94 (3.21-15.57)	2.84 (2.25-3.6)	4.31 (-4.31-7.96)	2.13 (1.66-2.74)	4.03 (-4.55-7.69)
Personality	Diabetes mellitus	Females	3.32 (3.2-3.45)	10.27 (9.88-10.66)	1.8 (1.73-1.87)	4.65 (4.24-5.04)	1.64 (1.58-1.71)	4.61 (4.19-5.01)
Intellectual	Diabetes mellitus	Females	5.37 (4.78-6.04)	13.9 (12.69-15.07)	2.9 (2.58-3.26)	7.85 (6.64-9.03)	1.4 (1.23-1.59)	2.94 (1.66-4.18)
Developmental	Diabetes mellitus	Females	4.07 (2.12-7.82)	NA	2.2 (1.14-4.23)	NA	1.26 (0.63-2.5)	NA
Behavioral	Diabetes mellitus	Females	4.62 (3.84-5.57)	15.15 (12.65-17.44)	2.5 (2.07-3.01)	7.82 (5.34-10.08)	2.08 (1.69-2.55)	7.45 (4.57-9.86)
Organic	Thyroid disorders	Persons	3.09 (3.03-3.16)	5.61 (5.49-5.73)	2.42 (2.37-2.47)	4.69 (4.57-4.81)	1.07 (1.04-1.09)	0.27 (0.15-0.37)
Substance Use	Thyroid disorders	Persons	3.49 (3.36-3.61)	9.99 (9.67-10.32)	2.72 (2.62-2.82)	8.36 (8.02-8.68)	0.87 (0.84-0.9)	0.47 (0.13-0.82)
Schizophrenia	Thyroid disorders	Persons	2.72 (2.62-2.83)	8.5 (8.12-8.86)	2.14 (2.06-2.23)	6.97 (6.59-7.33)	1.03 (0.99-1.07)	1.04 (0.66-1.4)
Mood	Thyroid disorders	Persons	2.04 (1.99-2.08)	5.55 (5.33-5.76)	1.6 (1.56-1.64)	4.17 (3.94-4.39)	1.04 (1.02-1.07)	0.47 (0.23-0.69)
Neurotic	Thyroid disorders	Persons	2.13 (2.07-2.2)	6.11 (5.83-6.4)	1.67 (1.62-1.72)	4.6 (4.3-4.88)	0.98 (0.95-1.01)	0.52 (0.22-0.82)

© 2022 Momen NC et al. *JAMA Psychiatry*.

Mental disorder	General medical condition	Sex	Compared to those with neither MD nor GMC		Compared to those with GMC only		Compared to those with MD only	
			MRR	LYLs	MRR	LYLs	MRR	LYLs
Eating	Thyroid disorders	Persons	3.2 (2.47-4.14)	6.11 (-1.8-10.24)	2.53 (1.95-3.27)	4.52 (-3.49-8.68)	1.2 (0.91-1.57)	-1.83 (-9.91-2.63)
Personality	Thyroid disorders	Persons	2.17 (2.09-2.25)	6.36 (5.97-6.7)	1.7 (1.64-1.77)	4.85 (4.46-5.22)	0.94 (0.91-0.98)	0.38 (-0.03-0.76)
Intellectual	Thyroid disorders	Persons	5.11 (4.6-5.67)	14.45 (13.05-15.6)	4.03 (3.63-4.48)	12.39 (10.99-13.52)	1.44 (1.29-1.6)	3.2 (1.81-4.36)
Developmental	Thyroid disorders	Persons	3.77 (2.46-5.78)	NA	2.97 (1.94-4.56)	NA	1.5 (0.97-2.33)	NA
Behavioral	Thyroid disorders	Persons	2.65 (2.2-3.2)	8.85 (5.46-10.81)	2.09 (1.74-2.53)	6.58 (3.17-8.52)	0.96 (0.79-1.16)	0.85 (-2.6-2.9)
Organic	Thyroid disorders	Males	3.44 (3.28-3.61)	6.4 (6.1-6.71)	2.5 (2.38-2.62)	4.97 (4.68-5.28)	1.11 (1.06-1.16)	0.35 (0.1-0.6)
Substance Use	Thyroid disorders	Males	4.05 (3.79-4.33)	10.56 (9.98-11.1)	2.91 (2.72-3.12)	8.03 (7.45-8.58)	0.94 (0.88-1)	0.93 (0.36-1.47)
Schizophrenia	Thyroid disorders	Males	3.47 (3.19-3.78)	10.63 (9.73-11.41)	2.54 (2.33-2.77)	7.93 (7.03-8.71)	1.13 (1.04-1.23)	2.21 (1.36-2.99)
Mood	Thyroid disorders	Males	2.32 (2.19-2.45)	6.51 (5.98-6.98)	1.69 (1.6-1.79)	4.2 (3.66-4.68)	1.08 (1.02-1.14)	0.89 (0.36-1.38)
Neurotic	Thyroid disorders	Males	2.75 (2.57-2.95)	8.44 (7.76-9.11)	2.01 (1.87-2.15)	5.74 (5.03-6.41)	1.11 (1.04-1.19)	2 (1.34-2.68)
Eating	Thyroid disorders	Males	NA	NA	NA	NA	NA	NA
Personality	Thyroid disorders	Males	2.49 (2.29-2.71)	7.53 (6.57-8.26)	1.81 (1.66-1.97)	4.94 (3.99-5.66)	0.97 (0.89-1.06)	0.99 (-0.01-1.69)
Intellectual	Thyroid disorders	Males	5.6 (4.69-6.68)	14.67 (11.63-16.83)	4.1 (3.43-4.9)	11.34 (8.28-13.46)	1.65 (1.37-1.98)	4.32 (1.41-6.52)
Developmental	Thyroid disorders	Males	4.43 (2.57-7.62)	NA	3.24 (1.88-5.57)	NA	1.9 (1.09-3.32)	NA
Behavioral	Thyroid disorders	Males	3.75 (2.7-5.2)	11.44 (2.44-15.41)	2.74 (1.98-3.8)	7.27 (-1.73-11.39)	1.24 (0.89-1.73)	2.75 (-6.3-6.87)
Organic	Thyroid disorders	Females	3 (2.93-3.07)	5.44 (5.3-5.56)	2.41 (2.36-2.47)	4.63 (4.48-4.76)	1.09 (1.07-1.12)	0.25 (0.12-0.37)
Substance Use	Thyroid disorders	Females	3.27 (3.14-3.42)	9.77 (9.38-10.15)	2.63 (2.52-2.75)	8.49 (8.09-8.87)	0.93 (0.89-0.97)	0.29 (-0.11-0.71)
Schizophrenia	Thyroid disorders	Females	2.57 (2.47-2.68)	7.99 (7.59-8.39)	2.08 (1.99-2.17)	6.74 (6.34-7.15)	1.1 (1.05-1.15)	0.76 (0.36-1.17)
Mood	Thyroid disorders	Females	1.98 (1.93-2.02)	5.38 (5.13-5.61)	1.59 (1.55-1.64)	4.17 (3.9-4.41)	1.08 (1.05-1.11)	0.4 (0.13-0.64)
Neurotic	Thyroid disorders	Females	2.02 (1.96-2.09)	5.74 (5.41-6.04)	1.63 (1.57-1.68)	4.41 (4.08-4.73)	1.02 (0.99-1.05)	0.29 (-0.05-0.62)
Eating	Thyroid disorders	Females	3.08 (2.36-4.02)	6.6 (-1.43-10.76)	2.49 (1.9-3.25)	5.08 (-2.98-9.24)	1.2 (0.9-1.58)	-1.27 (-9.37-3.11)
Personality	Thyroid disorders	Females	2.1 (2.02-2.18)	6.16 (5.75-6.56)	1.69 (1.62-1.76)	4.84 (4.42-5.25)	1 (0.96-1.05)	0.28 (-0.19-0.71)
Intellectual	Thyroid disorders	Females	4.87 (4.29-5.54)	14.35 (12.87-15.83)	3.94 (3.46-4.48)	12.9 (11.41-14.38)	1.29 (1.12-1.48)	2.65 (1.13-4.18)
Developmental	Thyroid disorders	Females	3.04 (1.52-6.07)	NA	2.45 (1.23-4.9)	NA	0.93 (0.45-1.91)	NA
Behavioral	Thyroid disorders	Females	2.31 (1.84-2.91)	7.89 (4.62-10.25)	1.87 (1.49-2.35)	6.33 (3.08-8.7)	0.97 (0.76-1.23)	0.16 (-3.19-2.7)
Organic	Gout	Persons	3.99 (3.77-4.22)	6.32 (6.03-6.65)	1.9 (1.79-2.01)	3.13 (2.87-3.42)	1.39 (1.31-1.47)	1.05 (0.82-1.31)
Substance Use	Gout	Persons	4.45 (4.16-4.76)	11.01 (10.39-11.6)	2.18 (2.04-2.34)	5.18 (4.59-5.77)	1.13 (1.06-1.21)	1.75 (1.17-2.29)

© 2022 Momen NC et al. *JAMA Psychiatry*.

Mental disorder	General medical condition	Sex	Compared to those with neither MD nor GMC		Compared to those with GMC only		Compared to those with MD only	
			MRR	LYLs	MRR	LYLs	MRR	LYLs
Schizophrenia	Gout	Persons	3.32 (2.93-3.77)	9.25 (7.95-10.4)	1.63 (1.44-1.85)	3.76 (2.5-4.88)	1.27 (1.12-1.45)	2.44 (1.14-3.58)
Mood	Gout	Persons	2.83 (2.67-3.01)	7.75 (7.25-8.24)	1.38 (1.3-1.47)	2.76 (2.26-3.23)	1.47 (1.38-1.56)	3.21 (2.73-3.68)
Neurotic	Gout	Persons	3.2 (3-3.43)	8.94 (8.37-9.53)	1.58 (1.48-1.69)	3.05 (2.45-3.64)	1.5 (1.4-1.61)	3.59 (3-4.16)
Eating	Gout	Persons	5.56 (2.31-13.36)	NA	2.76 (1.15-6.63)	NA	2.1 (0.87-5.07)	NA
Personality	Gout	Persons	3.28 (3-3.59)	9.14 (8.29-9.86)	1.62 (1.48-1.77)	3.37 (2.54-4.08)	1.46 (1.34-1.6)	3.77 (2.94-4.46)
Intellectual	Gout	Persons	7.12 (5.09-9.97)	NA	3.53 (2.52-4.94)	NA	1.96 (1.4-2.75)	NA
Developmental	Gout	Persons	NA	NA	NA	NA	NA	NA
Behavioral	Gout	Persons	3.4 (2.21-5.21)	NA	1.69 (1.1-2.59)	NA	1.24 (0.8-1.9)	NA
Organic	Gout	Males	4.13 (3.85-4.42)	6.81 (6.42-7.27)	2 (1.87-2.15)	3.62 (3.27-4)	1.32 (1.23-1.42)	1.08 (0.77-1.4)
Substance Use	Gout	Males	4.53 (4.2-4.89)	11.02 (10.33-11.68)	2.24 (2.07-2.42)	5.4 (4.71-6.05)	1.05 (0.97-1.13)	1.47 (0.82-2.09)
Schizophrenia	Gout	Males	3.26 (2.75-3.85)	9.45 (7.76-11)	1.62 (1.36-1.91)	4.08 (2.38-5.63)	1.05 (0.89-1.25)	1.85 (0.16-3.38)
Mood	Gout	Males	2.93 (2.71-3.16)	8.15 (7.51-8.79)	1.45 (1.34-1.57)	3.37 (2.76-3.99)	1.36 (1.26-1.48)	3.06 (2.47-3.67)
Neurotic	Gout	Males	3.33 (3.05-3.63)	8.93 (8.18-9.64)	1.66 (1.52-1.81)	3.41 (2.66-4.11)	1.34 (1.23-1.47)	2.97 (2.21-3.67)
Eating	Gout	Males	NA	NA	NA	NA	NA	NA
Personality	Gout	Males	3.36 (3-3.76)	9.07 (7.98-10.04)	1.68 (1.5-1.88)	3.66 (2.59-4.6)	1.32 (1.18-1.48)	3.11 (2.04-4.05)
Intellectual	Gout	Males	6.72 (4.47-10.11)	NA	3.38 (2.24-5.08)	NA	1.93 (1.28-2.91)	NA
Developmental	Gout	Males	NA	NA	NA	NA	NA	NA
Behavioral	Gout	Males	3.6 (2.24-5.8)	NA	1.81 (1.13-2.91)	NA	1.18 (0.73-1.91)	NA
Organic	Gout	Females	3.76 (3.41-4.14)	5.38 (4.95-5.86)	1.7 (1.54-1.88)	2.18 (1.79-2.6)	1.38 (1.25-1.52)	0.99 (0.62-1.39)
Substance Use	Gout	Females	4.21 (3.61-4.91)	11.01 (9.64-12.35)	2.03 (1.73-2.37)	4.19 (2.83-5.48)	1.23 (1.06-1.44)	2.98 (1.68-4.21)
Schizophrenia	Gout	Females	3.42 (2.83-4.13)	8.86 (6.83-10.63)	1.64 (1.35-1.98)	3.17 (1.34-4.92)	1.48 (1.22-1.79)	3.52 (1.63-5.17)
Mood	Gout	Females	2.71 (2.46-2.98)	7.08 (6.21-7.9)	1.28 (1.16-1.41)	1.74 (0.88-2.5)	1.5 (1.36-1.65)	3.47 (2.62-4.25)
Neurotic	Gout	Females	3.03 (2.72-3.38)	8.96 (8-9.95)	1.45 (1.3-1.62)	2.37 (1.34-3.4)	1.56 (1.4-1.74)	4.76 (3.8-5.72)
Eating	Gout	Females	NA	NA	NA	NA	NA	NA
Personality	Gout	Females	3.16 (2.73-3.66)	9.27 (7.74-10.49)	1.52 (1.31-1.77)	2.86 (1.35-4.05)	1.55 (1.34-1.8)	4.93 (3.48-6.07)
Intellectual	Gout	Females	8.13 (4.5-14.67)	NA	3.93 (2.17-7.1)	NA	2.13 (1.18-3.85)	NA
Developmental	Gout	Females	NA	NA	NA	NA	NA	NA

© 2022 Momen NC et al. *JAMA Psychiatry*.

Mental disorder	General medical condition	Sex	Compared to those with neither MD nor GMC		Compared to those with GMC only		Compared to those with MD only	
			MRR	LYLs	MRR	LYLs	MRR	LYLs
Behavioral	Gout	Females	NA	NA	NA	NA	NA	NA
Organic	Pulmonary system and allergy	Persons	4.06 (4.01-4.11)	6.58 (6.5-6.65)	2.41 (2.38-2.44)	4.97 (4.9-5.04)	1.3 (1.28-1.32)	0.62 (0.55-0.7)
Substance Use	Pulmonary system and allergy	Persons	5.57 (5.49-5.64)	13.62 (13.48-13.77)	3.41 (3.37-3.46)	10.72 (10.58-10.86)	1.41 (1.39-1.44)	2.15 (1.99-2.32)
Schizophrenia	Pulmonary system and allergy	Persons	4.13 (4.05-4.21)	12.17 (11.96-12.39)	2.5 (2.45-2.55)	9.35 (9.13-9.57)	1.58 (1.54-1.62)	3.04 (2.79-3.3)
Mood	Pulmonary system and allergy	Persons	2.93 (2.89-2.96)	8.35 (8.23-8.5)	1.78 (1.76-1.8)	5.68 (5.55-5.83)	1.54 (1.51-1.56)	2.19 (2.04-2.37)
Neurotic	Pulmonary system and allergy	Persons	3.16 (3.12-3.2)	9.02 (8.88-9.17)	1.93 (1.91-1.96)	6.12 (5.98-6.27)	1.52 (1.5-1.55)	2.3 (2.13-2.48)
Eating	Pulmonary system and allergy	Persons	4.01 (3.58-4.49)	10.08 (7.01-11.92)	2.42 (2.16-2.72)	7.1 (4.05-8.91)	1.54 (1.31-1.81)	1.65 (-1.6-4.11)
Personality	Pulmonary system and allergy	Persons	3.4 (3.34-3.45)	9.62 (9.46-9.8)	2.07 (2.04-2.11)	6.74 (6.58-6.92)	1.6 (1.56-1.64)	2.55 (2.34-2.77)
Intellectual	Pulmonary system and allergy	Persons	5.52 (5.22-5.84)	14.36 (13.59-15.07)	3.34 (3.15-3.53)	11.23 (10.46-11.94)	1.46 (1.36-1.57)	2.33 (1.49-3.13)
Developmental	Pulmonary system and allergy	Persons	3.41 (2.85-4.09)	8.17 (-1.37-10.5)	2.06 (1.72-2.47)	4.86 (-4.67-7.2)	1.19 (0.96-1.49)	0.44 (-9.08-2.97)
Behavioral	Pulmonary system and allergy	Persons	3.62 (3.37-3.89)	10.78 (9.76-11.66)	2.19 (2.04-2.35)	7.54 (6.51-8.42)	1.21 (1.1-1.33)	2.07 (0.88-3.14)
Organic	Pulmonary system and allergy	Males	4.29 (4.21-4.37)	7.01 (6.89-7.12)	2.52 (2.47-2.56)	5.25 (5.14-5.36)	1.27 (1.24-1.29)	0.64 (0.53-0.76)
Substance Use	Pulmonary system and allergy	Males	5.89 (5.78-5.99)	13.89 (13.71-14.08)	3.54 (3.48-3.61)	10.86 (10.67-11.05)	1.3 (1.27-1.33)	2.17 (1.97-2.39)
Schizophrenia	Pulmonary system and allergy	Males	4.65 (4.51-4.8)	13.31 (12.96-13.65)	2.77 (2.69-2.86)	10.24 (9.89-10.57)	1.45 (1.4-1.51)	3.14 (2.77-3.53)
Mood	Pulmonary system and allergy	Males	3.17 (3.1-3.23)	8.99 (8.77-9.22)	1.89 (1.85-1.93)	6.15 (5.94-6.39)	1.45 (1.41-1.49)	2.03 (1.79-2.3)
Neurotic	Pulmonary system and allergy	Males	3.55 (3.48-3.63)	10.24 (9.98-10.47)	2.13 (2.08-2.18)	7.12 (6.86-7.37)	1.4 (1.36-1.44)	2.32 (2.03-2.61)
Eating	Pulmonary system and allergy	Males	5.39 (3.77-7.71)	14.47 (5.72-19.6)	3.22 (2.25-4.61)	11.08 (2.34-16.21)	1.43 (0.88-2.33)	3.65 (-6.08-9.78)
Personality	Pulmonary system and allergy	Males	3.78 (3.68-3.87)	10.62 (10.36-10.9)	2.27 (2.21-2.33)	7.58 (7.32-7.86)	1.49 (1.44-1.55)	2.73 (2.43-3.08)
Intellectual	Pulmonary system and allergy	Males	5.22 (4.83-5.65)	14.06 (13.01-15.04)	3.12 (2.88-3.38)	10.75 (9.7-11.74)	1.43 (1.3-1.58)	2.56 (1.34-3.68)

© 2022 Momen NC et al. *JAMA Psychiatry*.

Mental disorder	General medical condition	Sex	Compared to those with neither MD nor GMC		Compared to those with GMC only		Compared to those with MD only	
			MRR	LYLs	MRR	LYLs	MRR	LYLs
Developmental	Pulmonary system and allergy	Males	3.38 (2.75-4.15)	7.69 (-0.91-11.27)	2.02 (1.64-2.48)	4.26 (-4.31-7.86)	1.3 (1.01-1.68)	1 (-7.5-4.64)
Behavioral	Pulmonary system and allergy	Males	3.97 (3.61-4.36)	11.51 (10.17-12.79)	2.37 (2.16-2.6)	8.09 (6.73-9.36)	1.18 (1.04-1.33)	2.38 (0.69-3.96)
Organic	Pulmonary system and allergy	Females	3.9 (3.84-3.96)	6.28 (6.19-6.38)	2.34 (2.3-2.37)	4.78 (4.69-4.88)	1.32 (1.29-1.34)	0.61 (0.51-0.71)
Substance Use	Pulmonary system and allergy	Females	5.22 (5.12-5.33)	13.29 (13.08-13.49)	3.26 (3.19-3.32)	10.54 (10.33-10.75)	1.73 (1.67-1.78)	2.12 (1.86-2.38)
Schizophrenia	Pulmonary system and allergy	Females	3.83 (3.73-3.92)	11.34 (11.06-11.62)	2.35 (2.29-2.41)	8.71 (8.43-8.98)	1.73 (1.67-1.79)	2.97 (2.66-3.29)
Mood	Pulmonary system and allergy	Females	2.79 (2.75-2.84)	8.05 (7.89-8.22)	1.73 (1.7-1.75)	5.45 (5.29-5.63)	1.6 (1.57-1.63)	2.26 (2.07-2.48)
Neurotic	Pulmonary system and allergy	Females	2.95 (2.9-3)	8.4 (8.22-8.57)	1.84 (1.8-1.87)	5.6 (5.42-5.8)	1.67 (1.63-1.71)	2.29 (2.08-2.51)
Eating	Pulmonary system and allergy	Females	3.87 (3.44-4.37)	9.78 (6.59-11.76)	2.37 (2.11-2.68)	6.83 (3.61-8.8)	1.56 (1.32-1.85)	1.52 (-1.67-4.14)
Personality	Pulmonary system and allergy	Females	3.17 (3.1-3.23)	9.15 (8.94-9.39)	1.96 (1.92-2.01)	6.36 (6.15-6.59)	1.75 (1.69-1.8)	2.46 (2.21-2.74)
Intellectual	Pulmonary system and allergy	Females	5.86 (5.41-6.36)	14.77 (13.79-15.65)	3.59 (3.31-3.89)	11.91 (10.93-12.79)	1.49 (1.34-1.65)	2.02 (0.8-3.12)
Developmental	Pulmonary system and allergy	Females	3.58 (2.47-5.18)	9.62 (-14.43-13.87)	2.19 (1.51-3.17)	6.68 (-17.37-10.95)	0.92 (0.59-1.43)	-1.25 (-24.28-3.85)
Behavioral	Pulmonary system and allergy	Females	3.24 (2.91-3.62)	9.55 (8.19-10.75)	1.99 (1.78-2.22)	6.61 (5.26-7.81)	1.35 (1.15-1.57)	1.55 (-0.11-3.14)
Organic	Chronic pulmonary disease	Persons	4.5 (4.44-4.57)	7.21 (7.12-7.31)	2.13 (2.1-2.16)	4.4 (4.31-4.5)	1.43 (1.41-1.45)	1.09 (1-1.17)
Substance Use	Chronic pulmonary disease	Persons	6.19 (6.1-6.29)	14.36 (14.21-14.52)	3.07 (3.02-3.11)	9.18 (9.02-9.34)	1.59 (1.56-1.62)	3.19 (3.02-3.36)
Schizophrenia	Chronic pulmonary disease	Persons	4.94 (4.83-5.05)	13.63 (13.39-13.9)	2.41 (2.36-2.47)	8.55 (8.32-8.81)	1.91 (1.86-1.96)	4.68 (4.41-4.97)
Mood	Chronic pulmonary disease	Persons	3.48 (3.44-3.53)	9.83 (9.67-9.97)	1.7 (1.68-1.73)	5.07 (4.91-5.22)	1.83 (1.8-1.86)	3.81 (3.64-4)
Neurotic	Chronic pulmonary disease	Persons	3.77 (3.71-3.82)	10.74 (10.57-10.9)	1.86 (1.83-1.89)	5.48 (5.32-5.65)	1.85 (1.81-1.88)	4.11 (3.92-4.3)
Eating	Chronic pulmonary disease	Persons	4.77 (4.17-5.46)	13.46 (10.69-14.95)	2.33 (2.03-2.67)	7.97 (5.16-9.45)	1.85 (1.57-2.19)	5.01 (2.26-7.1)

© 2022 Momen NC et al. *JAMA Psychiatry*.

Mental disorder	General medical condition	Sex	Compared to those with neither MD nor GMC		Compared to those with GMC only		Compared to those with MD only	
			MRR	LYLs	MRR	LYLs	MRR	LYLs
Personality	Chronic pulmonary disease	Persons	4.02 (3.95-4.09)	11.15 (10.93-11.36)	1.98 (1.94-2.01)	6 (5.79-6.21)	1.91 (1.87-1.96)	4.29 (4.07-4.52)
Intellectual	Chronic pulmonary disease	Persons	6.43 (6-6.9)	16.95 (16.02-18.03)	3.14 (2.92-3.36)	11.15 (10.22-12.21)	1.69 (1.56-1.83)	4.87 (3.87-5.99)
Developmental	Chronic pulmonary disease	Persons	3.63 (2.87-4.58)	8.63 (-1.19-13.18)	1.77 (1.4-2.23)	2.46 (-7.38-7.02)	1.27 (0.98-1.64)	0.94 (-8.97-5.85)
Behavioral	Chronic pulmonary disease	Persons	3.94 (3.63-4.27)	12.63 (11.38-13.85)	1.92 (1.77-2.09)	6.6 (5.37-7.8)	1.35 (1.23-1.49)	3.92 (2.48-5.23)
Organic	Chronic pulmonary disease	Males	4.65 (4.55-4.75)	7.42 (7.28-7.56)	2.18 (2.13-2.23)	4.49 (4.36-4.62)	1.36 (1.32-1.39)	1.07 (0.94-1.19)
Substance Use	Chronic pulmonary disease	Males	6.4 (6.27-6.53)	14.5 (14.3-14.73)	3.12 (3.06-3.19)	9.11 (8.9-9.33)	1.43 (1.4-1.47)	3.15 (2.92-3.39)
Schizophrenia	Chronic pulmonary disease	Males	5.46 (5.27-5.65)	14.72 (14.32-15.09)	2.63 (2.54-2.73)	9.2 (8.81-9.58)	1.73 (1.66-1.8)	4.82 (4.38-5.22)
Mood	Chronic pulmonary disease	Males	3.7 (3.61-3.78)	10.29 (10.03-10.53)	1.78 (1.74-1.83)	5.33 (5.05-5.57)	1.7 (1.66-1.75)	3.59 (3.3-3.85)
Neurotic	Chronic pulmonary disease	Males	4.1 (4-4.2)	11.9 (11.61-12.2)	1.99 (1.94-2.04)	6.27 (5.96-6.57)	1.64 (1.59-1.69)	4.09 (3.76-4.42)
Eating	Chronic pulmonary disease	Males	5.27 (3.44-8.09)	NA	2.55 (1.66-3.91)	NA	1.34 (0.8-2.26)	NA
Personality	Chronic pulmonary disease	Males	4.36 (4.24-4.48)	11.82 (11.52-12.13)	2.12 (2.06-2.18)	6.43 (6.13-6.76)	1.75 (1.69-1.81)	4.28 (3.94-4.64)
Intellectual	Chronic pulmonary disease	Males	6.2 (5.6-6.85)	17.2 (15.66-18.73)	2.99 (2.71-3.31)	11.03 (9.54-12.59)	1.69 (1.51-1.89)	5.59 (4.06-7.22)
Developmental	Chronic pulmonary disease	Males	3.57 (2.73-4.67)	9.6 (-0.71-14.71)	1.73 (1.32-2.26)	3.2 (-7.15-8.36)	1.35 (1-1.82)	2.91 (-7.47-7.96)
Behavioral	Chronic pulmonary disease	Males	4.12 (3.7-4.6)	13.62 (11.92-15.15)	1.99 (1.79-2.22)	7.23 (5.51-8.78)	1.24 (1.09-1.41)	4.49 (2.56-6.36)
Organic	Chronic pulmonary disease	Females	4.39 (4.31-4.47)	7.06 (6.93-7.18)	2.09 (2.05-2.13)	4.34 (4.22-4.46)	1.47 (1.44-1.5)	1.1 (0.98-1.22)
Substance Use	Chronic pulmonary disease	Females	5.97 (5.84-6.11)	14.2 (13.96-14.42)	3 (2.93-3.07)	9.27 (9.03-9.5)	1.95 (1.89-2.01)	3.23 (2.95-3.5)
Schizophrenia	Chronic pulmonary disease	Females	4.63 (4.5-4.76)	12.87 (12.58-13.22)	2.29 (2.22-2.35)	8.1 (7.8-8.45)	2.08 (2.01-2.15)	4.58 (4.25-4.97)
Mood	Chronic pulmonary disease	Females	3.36 (3.3-3.42)	9.61 (9.41-9.79)	1.66 (1.64-1.7)	4.95 (4.76-5.14)	1.91 (1.87-1.95)	3.91 (3.68-4.14)

© 2022 Momen NC et al. *JAMA Psychiatry*.

Mental disorder	General medical condition	Sex	Compared to those with neither MD nor GMC		Compared to those with GMC only		Compared to those with MD only	
			MRR	LYLs	MRR	LYLs	MRR	LYLs
Neurotic	Chronic pulmonary disease	Females	3.58 (3.52-3.65)	10.15 (9.95-10.34)	1.79 (1.76-1.83)	5.09 (4.89-5.29)	2.04 (1.99-2.09)	4.12 (3.88-4.36)
Eating	Chronic pulmonary disease	Females	4.71 (4.08-5.43)	13.27 (10.48-14.81)	2.32 (2.01-2.67)	7.83 (5.02-9.39)	1.92 (1.6-2.29)	5 (2.21-7.31)
Personality	Chronic pulmonary disease	Females	3.81 (3.72-3.9)	10.84 (10.57-11.11)	1.9 (1.85-1.94)	5.8 (5.53-6.06)	2.1 (2.04-2.16)	4.29 (4.01-4.57)
Intellectual	Chronic pulmonary disease	Females	6.67 (6.05-7.36)	16.57 (15.41-17.81)	3.28 (2.98-3.62)	11.32 (10.18-12.56)	1.67 (1.49-1.88)	3.78 (2.43-5.19)
Developmental	Chronic pulmonary disease	Females	3.83 (2.38-6.16)	5.51 (-12.96-15.87)	1.88 (1.17-3.03)	0.08 (-18.34-10.41)	1.03 (0.61-1.74)	-5.4 (-24.58-5.64)
Behavioral	Chronic pulmonary disease	Females	3.73 (3.3-4.22)	10.89 (9.34-12.39)	1.84 (1.63-2.08)	5.47 (3.91-6.97)	1.62 (1.38-1.9)	2.9 (1.12-4.72)
Organic	Allergy	Persons	3.1 (3.05-3.14)	6.02 (5.91-6.12)	2.83 (2.79-2.88)	5.69 (5.59-5.8)	1.09 (1.07-1.11)	0.11 (0.01-0.21)
Substance Use	Allergy	Persons	4.1 (4.02-4.18)	12.34 (12.11-12.54)	3.76 (3.68-3.83)	11.98 (11.75-12.19)	1.04 (1.02-1.07)	0.68 (0.45-0.9)
Schizophrenia	Allergy	Persons	2.82 (2.74-2.9)	10.1 (9.78-10.43)	2.57 (2.49-2.64)	9.73 (9.41-10.06)	1.09 (1.05-1.12)	0.79 (0.44-1.13)
Mood	Allergy	Persons	2.1 (2.07-2.13)	6.74 (6.58-6.94)	1.93 (1.9-1.96)	6.36 (6.19-6.55)	1.1 (1.08-1.13)	0.44 (0.24-0.64)
Neurotic	Allergy	Persons	2.22 (2.18-2.26)	6.98 (6.78-7.19)	2.04 (2-2.07)	6.59 (6.39-6.8)	1.03 (1.01-1.06)	0.25 (0.02-0.48)
Eating	Allergy	Persons	2.85 (2.46-3.3)	5.28 (-1.4-8.12)	2.59 (2.23-3.01)	4.83 (-1.79-7.67)	1.07 (0.9-1.28)	-3.06 (-9.54-0.4)
Personality	Allergy	Persons	2.36 (2.31-2.42)	7.56 (7.31-7.8)	2.16 (2.11-2.21)	7.16 (6.91-7.41)	1.05 (1.03-1.08)	0.4 (0.13-0.66)
Intellectual	Allergy	Persons	4.28 (3.97-4.61)	12.99 (12.13-13.8)	3.89 (3.61-4.2)	12.63 (11.78-13.45)	1.21 (1.11-1.31)	1.04 (0.11-1.93)
Developmental	Allergy	Persons	2.64 (2.09-3.33)	4.35 (-9.23-8.31)	2.4 (1.9-3.03)	4.01 (-9.62-7.97)	1.02 (0.79-1.33)	-3.45 (-17.08-0.77)
Behavioral	Allergy	Persons	2.84 (2.57-3.14)	8.78 (7.35-9.93)	2.58 (2.34-2.86)	8.42 (6.99-9.6)	1.03 (0.92-1.15)	0.11 (-1.43-1.4)
Organic	Allergy	Males	3.31 (3.23-3.39)	6.48 (6.31-6.65)	3.07 (2.99-3.15)	6.17 (5.99-6.35)	1.08 (1.05-1.11)	0.09 (-0.07-0.24)
Substance Use	Allergy	Males	4.34 (4.23-4.46)	12.74 (12.46-13.04)	4.02 (3.91-4.14)	12.49 (12.19-12.8)	1 (0.98-1.04)	0.66 (0.34-0.99)
Schizophrenia	Allergy	Males	3.1 (2.95-3.24)	10.93 (10.39-11.47)	2.85 (2.72-2.99)	10.68 (10.14-11.21)	1 (0.95-1.05)	0.41 (-0.15-0.99)
Mood	Allergy	Males	2.26 (2.2-2.32)	7.38 (7.09-7.68)	2.1 (2.04-2.16)	7.11 (6.82-7.43)	1.06 (1.03-1.1)	0.17 (-0.14-0.5)
Neurotic	Allergy	Males	2.49 (2.41-2.57)	8.09 (7.77-8.42)	2.31 (2.24-2.38)	7.83 (7.51-8.17)	1 (0.97-1.03)	0.1 (-0.24-0.48)
Eating	Allergy	Males	4.46 (2.69-7.4)	NA	4.12 (2.48-6.83)	NA	1.24 (0.7-2.21)	NA
Personality	Allergy	Males	2.58 (2.48-2.67)	8.46 (7.99-8.89)	2.38 (2.29-2.48)	8.2 (7.74-8.63)	1.01 (0.96-1.05)	0.28 (-0.18-0.73)
Intellectual	Allergy	Males	3.98 (3.59-4.42)	12.27 (11.05-13.41)	3.68 (3.31-4.08)	11.99 (10.75-13.11)	1.17 (1.04-1.31)	0.95 (-0.41-2.17)

© 2022 Momen NC et al. *JAMA Psychiatry*.

Mental disorder	General medical condition	Sex	Compared to those with neither MD nor GMC		Compared to those with GMC only		Compared to those with MD only	
			MRR	LYLs	MRR	LYLs	MRR	LYLs
Developmental	Allergy	Males	2.69 (2.07-3.5)	3.92 (-9.56-9.46)	2.48 (1.91-3.23)	3.61 (-9.88-9.19)	1.15 (0.86-1.54)	-2.76 (-16.51-3.1)
Behavioral	Allergy	Males	3.38 (2.96-3.85)	9.52 (7.75-11.21)	3.12 (2.73-3.55)	9.23 (7.43-10.94)	1.12 (0.97-1.3)	0.38 (-1.6-2.23)
Organic	Allergy	Females	2.97 (2.91-3.03)	5.72 (5.59-5.85)	2.69 (2.63-2.74)	5.39 (5.26-5.52)	1.1 (1.08-1.12)	0.12 (0-0.25)
Substance Use	Allergy	Females	3.86 (3.76-3.97)	11.89 (11.59-12.19)	3.52 (3.42-3.62)	11.43 (11.12-11.74)	1.16 (1.12-1.2)	0.7 (0.35-1.03)
Schizophrenia	Allergy	Females	2.68 (2.59-2.78)	9.55 (9.16-9.92)	2.42 (2.34-2.51)	9.1 (8.71-9.48)	1.18 (1.14-1.23)	1.04 (0.58-1.45)
Mood	Allergy	Females	2.02 (1.98-2.06)	6.46 (6.23-6.68)	1.84 (1.8-1.88)	6.02 (5.79-6.24)	1.14 (1.11-1.17)	0.56 (0.32-0.81)
Neurotic	Allergy	Females	2.09 (2.04-2.14)	6.46 (6.2-6.71)	1.9 (1.86-1.95)	6 (5.74-6.26)	1.09 (1.06-1.12)	0.32 (0.05-0.6)
Eating	Allergy	Females	2.76 (2.36-3.22)	4.91 (-1.83-8.09)	2.48 (2.13-2.9)	4.45 (-2.31-7.58)	1.08 (0.89-1.3)	-3.3 (-9.8-0.39)
Personality	Allergy	Females	2.26 (2.19-2.32)	7.2 (6.9-7.52)	2.05 (1.99-2.11)	6.74 (6.44-7.05)	1.13 (1.09-1.16)	0.44 (0.11-0.79)
Intellectual	Allergy	Females	4.64 (4.16-5.16)	13.84 (12.59-15.08)	4.17 (3.74-4.65)	13.39 (12.14-14.64)	1.24 (1.1-1.41)	1.14 (-0.26-2.52)
Developmental	Allergy	Females	2.46 (1.48-4.08)	5.52 (-28.07-10.84)	2.21 (1.33-3.66)	5.07 (-28.51-10.39)	0.71 (0.41-1.23)	-5.26 (-38.53-1.05)
Behavioral	Allergy	Females	2.33 (2-2.72)	7.75 (5.49-9.31)	2.1 (1.8-2.45)	7.3 (5.01-8.84)	0.97 (0.82-1.17)	-0.27 (-2.79-1.58)
Organic	Gastrointestinal system	Persons	3.9 (3.84-3.97)	7.06 (6.94-7.18)	1.8 (1.77-1.83)	3.97 (3.86-4.07)	1.25 (1.23-1.27)	1.23 (1.12-1.32)
Substance Use	Gastrointestinal system	Persons	7.51 (7.39-7.64)	16.33 (16.17-16.48)	3.81 (3.74-3.87)	8.09 (7.92-8.24)	2.17 (2.13-2.21)	5.86 (5.68-6.03)
Schizophrenia	Gastrointestinal system	Persons	4.88 (4.74-5.02)	14 (13.71-14.3)	2.35 (2.28-2.42)	6.5 (6.23-6.77)	1.88 (1.82-1.94)	6.24 (5.97-6.54)
Mood	Gastrointestinal system	Persons	3.08 (3.03-3.13)	9.57 (9.4-9.74)	1.47 (1.45-1.5)	3.53 (3.36-3.7)	1.58 (1.55-1.61)	4.63 (4.46-4.8)
Neurotic	Gastrointestinal system	Persons	3.84 (3.77-3.91)	11.82 (11.63-12.02)	1.87 (1.84-1.91)	4.22 (4.02-4.43)	1.88 (1.84-1.92)	6.16 (5.95-6.36)
Eating	Gastrointestinal system	Persons	8.37 (7.11-9.85)	19 (16.45-20.74)	4.03 (3.43-4.75)	8.94 (6.54-10.73)	3.62 (3-4.36)	11.18 (8.33-13.57)
Personality	Gastrointestinal system	Persons	4.24 (4.15-4.34)	12.6 (12.35-12.88)	2.07 (2.02-2.11)	4.93 (4.67-5.2)	2 (1.95-2.05)	6.48 (6.19-6.75)
Intellectual	Gastrointestinal system	Persons	6.06 (5.51-6.66)	15.28 (14.11-16.29)	2.92 (2.66-3.21)	6.6 (5.42-7.61)	1.63 (1.47-1.81)	5.06 (3.87-6.04)
Developmental	Gastrointestinal system	Persons	8.94 (6.63-12.06)	NA	4.31 (3.19-5.81)	NA	3.65 (2.66-5.02)	NA
Behavioral	Gastrointestinal system	Persons	5.81 (5.25-6.44)	16.41 (14.92-17.65)	2.8 (2.53-3.1)	5.09 (3.58-6.39)	2.27 (2.02-2.54)	8.19 (6.62-9.56)
Organic	Gastrointestinal system	Males	4.33 (4.21-4.44)	8.3 (8.07-8.49)	1.86 (1.81-1.91)	4.44 (4.27-4.6)	1.28 (1.24-1.32)	1.53 (1.37-1.69)
Substance Use	Gastrointestinal system	Males	8.63 (8.45-8.81)	16.73 (16.53-16.93)	4.09 (4-4.18)	7.66 (7.45-7.86)	2.28 (2.22-2.34)	5.88 (5.66-6.09)
Schizophrenia	Gastrointestinal system	Males	6.21 (5.96-6.48)	15.64 (15.21-16.07)	2.77 (2.66-2.89)	6.6 (6.22-7)	2.05 (1.95-2.15)	6.84 (6.4-7.26)
Mood	Gastrointestinal system	Males	3.68 (3.58-3.78)	10.74 (10.5-11.01)	1.64 (1.59-1.68)	3.62 (3.36-3.86)	1.71 (1.66-1.77)	4.99 (4.72-5.25)

© 2022 Momen NC et al. *JAMA Psychiatry*.

Mental disorder	General medical condition	Sex	Compared to those with neither MD nor GMC		Compared to those with GMC only		Compared to those with MD only	
			MRR	LYLs	MRR	LYLs	MRR	LYLs
Neurotic	Gastrointestinal system	Males	4.84 (4.7-4.97)	13.16 (12.87-13.46)	2.19 (2.13-2.25)	4.3 (4.01-4.59)	2.07 (2-2.14)	6.54 (6.24-6.84)
Eating	Gastrointestinal system	Males	11.62 (6.88-19.61)	NA	5.18 (3.07-8.75)	NA	3.45 (1.91-6.22)	NA
Personality	Gastrointestinal system	Males	5.22 (5.04-5.39)	13.87 (13.52-14.21)	2.35 (2.27-2.43)	4.99 (4.66-5.33)	2.19 (2.11-2.27)	6.82 (6.46-7.19)
Intellectual	Gastrointestinal system	Males	6 (5.26-6.84)	14.95 (13.27-16.37)	2.67 (2.34-3.05)	5.19 (3.5-6.57)	1.67 (1.45-1.92)	5.04 (3.34-6.47)
Developmental	Gastrointestinal system	Males	8.54 (6.04-12.08)	NA	3.81 (2.69-5.39)	NA	3.71 (2.57-5.37)	NA
Behavioral	Gastrointestinal system	Males	7.59 (6.67-8.63)	18.33 (16.53-19.77)	3.38 (2.98-3.85)	5.79 (3.99-7.16)	2.7 (2.34-3.12)	9.69 (7.63-11.34)
Organic	Gastrointestinal system	Females	3.62 (3.54-3.7)	6.15 (6-6.28)	1.79 (1.75-1.83)	3.61 (3.48-3.74)	1.23 (1.2-1.26)	1 (0.87-1.11)
Substance Use	Gastrointestinal system	Females	6.21 (6.04-6.37)	15.7 (15.42-15.98)	3.34 (3.25-3.43)	8.78 (8.49-9.05)	2.05 (1.98-2.11)	5.82 (5.52-6.1)
Schizophrenia	Gastrointestinal system	Females	4.08 (3.93-4.25)	12.51 (12.11-12.93)	2.12 (2.03-2.2)	6.41 (6.01-6.8)	1.76 (1.69-1.84)	5.71 (5.3-6.12)
Mood	Gastrointestinal system	Females	2.77 (2.71-2.83)	8.85 (8.62-9.07)	1.43 (1.4-1.46)	3.47 (3.26-3.69)	1.52 (1.48-1.56)	4.41 (4.18-4.63)
Neurotic	Gastrointestinal system	Females	3.29 (3.21-3.37)	10.89 (10.59-11.16)	1.73 (1.68-1.77)	4.17 (3.86-4.45)	1.76 (1.71-1.81)	5.9 (5.59-6.18)
Eating	Gastrointestinal system	Females	8.02 (6.76-9.52)	18.98 (16.43-20.83)	4.16 (3.5-4.94)	9.03 (6.56-10.93)	3.65 (3-4.45)	11.27 (8.49-13.86)
Personality	Gastrointestinal system	Females	3.64 (3.52-3.75)	11.7 (11.33-12.05)	1.91 (1.85-1.97)	4.89 (4.53-5.25)	1.88 (1.81-1.94)	6.23 (5.85-6.61)
Intellectual	Gastrointestinal system	Females	6.14 (5.36-7.04)	15.67 (14.17-17.11)	3.18 (2.78-3.65)	8.27 (6.79-9.64)	1.58 (1.36-1.83)	5.09 (3.53-6.59)
Developmental	Gastrointestinal system	Females	10.69 (5.92-19.3)	NA	5.54 (3.07-10)	NA	3.51 (1.87-6.57)	NA
Behavioral	Gastrointestinal system	Females	4.16 (3.52-4.92)	13.66 (11.22-15.9)	2.16 (1.82-2.55)	4.09 (1.53-6.28)	1.87 (1.54-2.25)	6.04 (3.38-8.43)
Organic	Ulcer/chronic gastritis	Persons	3.94 (3.86-4.03)	6.86 (6.73-7.01)	1.67 (1.63-1.7)	2.98 (2.87-3.11)	1.31 (1.28-1.34)	1.35 (1.23-1.46)
Substance Use	Ulcer/chronic gastritis	Persons	6.5 (6.35-6.66)	14.86 (14.64-15.08)	2.95 (2.88-3.03)	5.9 (5.68-6.11)	1.71 (1.67-1.75)	5.2 (4.99-5.42)
Schizophrenia	Ulcer/chronic gastritis	Persons	4.68 (4.5-4.86)	12.94 (12.54-13.35)	2.07 (1.99-2.15)	4.72 (4.36-5.1)	1.79 (1.72-1.87)	5.83 (5.47-6.22)
Mood	Ulcer/chronic gastritis	Persons	3.19 (3.12-3.26)	9.38 (9.16-9.58)	1.4 (1.37-1.43)	2.36 (2.15-2.58)	1.65 (1.61-1.69)	4.79 (4.57-5)
Neurotic	Ulcer/chronic gastritis	Persons	3.82 (3.72-3.91)	11.56 (11.31-11.8)	1.7 (1.66-1.74)	2.8 (2.55-3.04)	1.82 (1.77-1.87)	6.19 (5.94-6.44)
Eating	Ulcer/chronic gastritis	Persons	7.03 (5.61-8.82)	NA	3.11 (2.48-3.9)	NA	2.82 (2.21-3.59)	NA
Personality	Ulcer/chronic gastritis	Persons	4.18 (4.05-4.31)	12.23 (11.92-12.55)	1.86 (1.8-1.92)	3.38 (3.07-3.7)	1.91 (1.85-1.97)	6.46 (6.15-6.78)
Intellectual	Ulcer/chronic gastritis	Persons	5.64 (5.01-6.35)	14.05 (12.53-15.28)	2.49 (2.21-2.81)	4.22 (2.78-5.33)	1.54 (1.36-1.74)	4.12 (2.69-5.32)
Developmental	Ulcer/chronic gastritis	Persons	7.53 (4.86-11.67)	NA	3.33 (2.15-5.16)	NA	3 (1.91-4.71)	NA
Behavioral	Ulcer/chronic gastritis	Persons	5.1 (4.36-5.98)	14.57 (12.03-16.16)	2.26 (1.93-2.65)	2.12 (-0.45-3.66)	1.89 (1.6-2.23)	6.57 (3.88-8.29)

© 2022 Momen NC et al. *JAMA Psychiatry*.

Mental disorder	General medical condition	Sex	Compared to those with neither MD nor GMC		Compared to those with GMC only		Compared to those with MD only	
			MRR	LYLs	MRR	LYLs	MRR	LYLs
Organic	Ulcer/chronic gastritis	Males	4.21 (4.07-4.35)	7.68 (7.45-7.91)	1.76 (1.7-1.82)	3.43 (3.25-3.61)	1.3 (1.25-1.34)	1.43 (1.24-1.6)
Substance Use	Ulcer/chronic gastritis	Males	7.2 (6.99-7.42)	15.17 (14.89-15.46)	3.22 (3.12-3.32)	5.96 (5.68-6.24)	1.72 (1.67-1.78)	5.1 (4.82-5.38)
Schizophrenia	Ulcer/chronic gastritis	Males	5.41 (5.1-5.74)	14.02 (13.42-14.61)	2.34 (2.21-2.49)	5.07 (4.56-5.61)	1.76 (1.65-1.87)	5.95 (5.42-6.53)
Mood	Ulcer/chronic gastritis	Males	3.54 (3.42-3.68)	10.14 (9.82-10.47)	1.53 (1.47-1.59)	2.82 (2.51-3.12)	1.65 (1.59-1.71)	4.82 (4.5-5.14)
Neurotic	Ulcer/chronic gastritis	Males	4.4 (4.24-4.58)	12.34 (11.97-12.72)	1.92 (1.85-2)	3.21 (2.83-3.58)	1.81 (1.74-1.89)	6.08 (5.71-6.44)
Eating	Ulcer/chronic gastritis	Males	9.91 (4.72-20.78)	NA	4.3 (2.05-9.02)	NA	2.79 (1.27-6.1)	NA
Personality	Ulcer/chronic gastritis	Males	4.79 (4.57-5.02)	12.96 (12.51-13.42)	2.09 (2-2.19)	3.82 (3.37-4.27)	1.93 (1.83-2.03)	6.36 (5.89-6.83)
Intellectual	Ulcer/chronic gastritis	Males	5.4 (4.59-6.36)	13.86 (11.66-15.57)	2.34 (1.99-2.76)	3.95 (1.84-5.58)	1.54 (1.3-1.82)	4.4 (2.23-6.15)
Developmental	Ulcer/chronic gastritis	Males	7.29 (4.46-11.89)	NA	3.16 (1.94-5.16)	NA	3.12 (1.88-5.17)	NA
Behavioral	Ulcer/chronic gastritis	Males	6.01 (4.87-7.41)	16.33 (12.45-18.19)	2.61 (2.11-3.22)	3.45 (-0.33-5.37)	2 (1.61-2.49)	7.86 (3.87-9.97)
Organic	Ulcer/chronic gastritis	Females	3.76 (3.65-3.87)	6.26 (6.09-6.44)	1.61 (1.56-1.66)	2.66 (2.51-2.81)	1.32 (1.28-1.36)	1.29 (1.15-1.44)
Substance Use	Ulcer/chronic gastritis	Females	5.69 (5.49-5.91)	14.41 (14.04-14.78)	2.63 (2.53-2.73)	5.82 (5.47-6.17)	1.72 (1.66-1.79)	5.35 (4.99-5.73)
Schizophrenia	Ulcer/chronic gastritis	Females	4.24 (4.03-4.46)	12.08 (11.54-12.61)	1.91 (1.81-2.01)	4.45 (3.94-4.94)	1.83 (1.73-1.93)	5.73 (5.23-6.24)
Mood	Ulcer/chronic gastritis	Females	3 (2.92-3.09)	8.92 (8.63-9.2)	1.34 (1.3-1.38)	2.09 (1.8-2.38)	1.66 (1.61-1.71)	4.76 (4.48-5.05)
Neurotic	Ulcer/chronic gastritis	Females	3.47 (3.36-3.59)	11.01 (10.66-11.35)	1.57 (1.52-1.63)	2.52 (2.17-2.84)	1.82 (1.76-1.89)	6.28 (5.91-6.6)
Eating	Ulcer/chronic gastritis	Females	6.82 (5.38-8.65)	NA	3.07 (2.42-3.9)	NA	2.84 (2.2-3.66)	NA
Personality	Ulcer/chronic gastritis	Females	3.79 (3.64-3.95)	11.73 (11.31-12.17)	1.72 (1.65-1.79)	3.07 (2.63-3.51)	1.91 (1.82-1.99)	6.54 (6.11-6.99)
Intellectual	Ulcer/chronic gastritis	Females	5.93 (4.99-7.05)	14.26 (12.45-16.17)	2.67 (2.25-3.18)	4.53 (2.78-6.34)	1.54 (1.28-1.84)	3.79 (2.05-5.69)
Developmental	Ulcer/chronic gastritis	Females	NA	NA	NA	NA	NA	NA
Behavioral	Ulcer/chronic gastritis	Females	4.26 (3.35-5.42)	12.45 (9.43-15.11)	1.92 (1.51-2.44)	0.53 (-2.5-3.09)	1.85 (1.44-2.39)	5.01 (1.82-7.97)
Organic	Chronic liver disease	Persons	10.27 (9.8-10.76)	16.44 (16.08-16.84)	1.22 (1.16-1.28)	2.39 (2.1-2.68)	3.59 (3.43-3.77)	5.42 (5.12-5.74)
Substance Use	Chronic liver disease	Persons	14.71 (14.4-15.03)	20.53 (20.35-20.74)	2 (1.95-2.05)	1.44 (1.24-1.66)	4.24 (4.15-4.35)	9.05 (8.83-9.27)
Schizophrenia	Chronic liver disease	Persons	14.08 (13.39-14.81)	21.21 (20.68-21.68)	1.73 (1.64-1.82)	1.28 (0.75-1.77)	5.53 (5.25-5.83)	11.78 (11.23-12.27)
Mood	Chronic liver disease	Persons	10.33 (9.96-10.71)	18.33 (17.99-18.66)	1.25 (1.21-1.3)	0.89 (0.57-1.21)	5.47 (5.27-5.67)	11.97 (11.65-12.3)
Neurotic	Chronic liver disease	Persons	12.83 (12.43-13.24)	20.02 (19.72-20.32)	1.6 (1.55-1.66)	0.88 (0.56-1.19)	6.29 (6.08-6.5)	13.44 (13.12-13.76)

© 2022 Momen NC et al. *JAMA Psychiatry*.

Mental disorder	General medical condition	Sex	Compared to those with neither MD nor GMC		Compared to those with GMC only		Compared to those with MD only	
			MRR	LYLs	MRR	LYLs	MRR	LYLs
Eating	Chronic liver disease	Persons	23.81 (18.67-30.36)	NA	2.92 (2.29-3.72)	NA	9.6 (7.42-12.42)	NA
Personality	Chronic liver disease	Persons	12.17 (11.73-12.63)	20.16 (19.78-20.54)	1.51 (1.45-1.57)	0.68 (0.3-1.04)	5.7 (5.49-5.93)	12.94 (12.52-13.32)
Intellectual	Chronic liver disease	Persons	14.77 (11.96-18.25)	NA	1.81 (1.46-2.23)	NA	4 (3.23-4.95)	NA
Developmental	Chronic liver disease	Persons	17.67 (10.47-29.84)	NA	2.16 (1.28-3.66)	NA	6.91 (4.05-11.8)	NA
Behavioral	Chronic liver disease	Persons	15.39 (13.36-17.73)	NA	1.89 (1.64-2.18)	NA	5.99 (5.16-6.96)	NA
Organic	Chronic liver disease	Males	11.33 (10.68-12.01)	16.89 (16.43-17.39)	1.25 (1.17-1.32)	2.15 (1.8-2.5)	3.66 (3.44-3.88)	5.45 (5.06-5.83)
Substance Use	Chronic liver disease	Males	14.97 (14.59-15.37)	20.14 (19.92-20.37)	1.84 (1.79-1.9)	1.04 (0.81-1.29)	3.95 (3.84-4.07)	8.57 (8.34-8.81)
Schizophrenia	Chronic liver disease	Males	14.74 (13.84-15.7)	20.94 (20.22-21.51)	1.66 (1.56-1.77)	0.71 (0.04-1.25)	4.93 (4.62-5.27)	11.05 (10.36-11.62)
Mood	Chronic liver disease	Males	10.97 (10.45-11.52)	17.85 (17.45-18.25)	1.22 (1.16-1.29)	0.31 (-0.04-0.68)	5.25 (4.99-5.52)	10.96 (10.57-11.35)
Neurotic	Chronic liver disease	Males	13.1 (12.56-13.66)	19.58 (19.15-19.97)	1.49 (1.42-1.55)	0.35 (-0.04-0.73)	5.61 (5.37-5.87)	12.34 (11.94-12.73)
Eating	Chronic liver disease	Males	25.96 (10.8-62.36)	NA	2.93 (1.22-7.03)	NA	7.16 (2.88-17.82)	NA
Personality	Chronic liver disease	Males	12.71 (12.12-13.34)	19.75 (19.32-20.17)	1.44 (1.37-1.51)	0.16 (-0.25-0.6)	5.32 (5.06-5.6)	11.87 (11.41-12.3)
Intellectual	Chronic liver disease	Males	14.13 (10.93-18.28)	NA	1.59 (1.23-2.06)	NA	3.98 (3.06-5.17)	NA
Developmental	Chronic liver disease	Males	18.26 (9.82-33.93)	NA	2.06 (1.11-3.83)	NA	7.63 (4.05-14.35)	NA
Behavioral	Chronic liver disease	Males	15.88 (13.45-18.74)	NA	1.79 (1.52-2.12)	NA	5.61 (4.7-6.69)	NA
Organic	Chronic liver disease	Females	8.87 (8.21-9.58)	15.67 (14.97-16.33)	1.21 (1.12-1.31)	2.78 (2.28-3.25)	3.28 (3.03-3.54)	5.36 (4.82-5.88)
Substance Use	Chronic liver disease	Females	14.32 (13.78-14.88)	21.34 (20.95-21.7)	2.31 (2.21-2.42)	2.26 (1.85-2.67)	4.7 (4.51-4.89)	10.04 (9.66-10.41)
Schizophrenia	Chronic liver disease	Females	13.13 (12.07-14.28)	21.68 (20.72-22.49)	1.87 (1.72-2.04)	2.28 (1.38-3.09)	5.77 (5.3-6.29)	13.07 (12.15-13.86)
Mood	Chronic liver disease	Females	9.63 (9.12-10.17)	18.9 (18.34-19.47)	1.36 (1.29-1.45)	1.59 (1.03-2.12)	5.42 (5.13-5.73)	13.18 (12.66-13.73)
Neurotic	Chronic liver disease	Females	12.54 (11.96-13.15)	20.57 (20.09-21.03)	1.87 (1.78-1.97)	1.53 (1.04-2)	6.71 (6.39-7.05)	14.79 (14.29-15.26)
Eating	Chronic liver disease	Females	23.57 (18.3-30.36)	NA	3.36 (2.6-4.33)	NA	9.93 (7.59-12.98)	NA
Personality	Chronic liver disease	Females	11.49 (10.84-12.17)	20.71 (20.04-21.32)	1.67 (1.57-1.78)	1.36 (0.67-1.98)	5.85 (5.51-6.21)	14.37 (13.68-14.99)

© 2022 Momen NC et al. *JAMA Psychiatry*.

Mental disorder	General medical condition	Sex	Compared to those with neither MD nor GMC		Compared to those with GMC only		Compared to those with MD only	
			MRR	LYLs	MRR	LYLs	MRR	LYLs
Intellectual	Chronic liver disease	Females	16.38 (11.31-23.73)	NA	2.32 (1.6-3.36)	NA	4.22 (2.91-6.14)	NA
Developmental	Chronic liver disease	Females	NA	NA	NA	NA	NA	NA
Behavioral	Chronic liver disease	Females	14.31 (10.9-18.77)	NA	2.03 (1.55-2.67)	NA	6.44 (4.85-8.55)	NA
Organic	Inflammatory bowel disease	Persons	3.7 (3.46-3.96)	7.65 (7.18-8.17)	2.48 (2.31-2.66)	5.77 (5.31-6.27)	1.3 (1.21-1.39)	1.07 (0.68-1.48)
Substance Use	Inflammatory bowel disease	Persons	4.6 (4.27-4.97)	12.84 (11.75-13.65)	3.12 (2.89-3.38)	9.69 (8.57-10.5)	1.18 (1.09-1.27)	1.57 (0.48-2.38)
Schizophrenia	Inflammatory bowel disease	Persons	3.72 (3.32-4.16)	11.63 (10.29-12.86)	2.52 (2.25-2.82)	8.43 (7.06-9.63)	1.43 (1.28-1.6)	2.56 (1.21-3.77)
Mood	Inflammatory bowel disease	Persons	2.61 (2.45-2.78)	8.57 (7.87-9.23)	1.78 (1.67-1.9)	5.33 (4.6-6.03)	1.36 (1.28-1.45)	2.24 (1.54-2.9)
Neurotic	Inflammatory bowel disease	Persons	2.88 (2.69-3.09)	9.09 (8.24-9.83)	1.97 (1.84-2.12)	5.63 (4.75-6.46)	1.36 (1.26-1.45)	2.4 (1.54-3.17)
Eating	Inflammatory bowel disease	Persons	5.88 (3.79-9.11)	NA	4 (2.58-6.2)	NA	2.25 (1.44-3.51)	NA
Personality	Inflammatory bowel disease	Persons	3.01 (2.75-3.29)	9.48 (8.26-10.39)	2.05 (1.87-2.24)	6 (4.8-6.93)	1.35 (1.23-1.47)	2.35 (1.13-3.27)
Intellectual	Inflammatory bowel disease	Persons	4.34 (3.18-5.92)	NA	2.95 (2.16-4.02)	NA	1.2 (0.88-1.63)	NA
Developmental	Inflammatory bowel disease	Persons	11.03 (5.93-20.5)	NA	7.5 (4.03-13.94)	NA	4.41 (2.35-8.27)	NA
Behavioral	Inflammatory bowel disease	Persons	2.77 (1.84-4.16)	NA	1.88 (1.25-2.83)	NA	1.01 (0.67-1.52)	NA
Organic	Inflammatory bowel disease	Males	3.65 (3.29-4.05)	8.12 (7.28-8.95)	2.58 (2.31-2.87)	6.49 (5.69-7.33)	1.18 (1.07-1.32)	0.97 (0.3-1.59)
Substance Use	Inflammatory bowel disease	Males	4.62 (4.14-5.15)	12.73 (11.14-13.94)	3.26 (2.92-3.65)	10.11 (8.57-11.29)	1.08 (0.97-1.2)	0.98 (-0.6-2.15)
Schizophrenia	Inflammatory bowel disease	Males	3.63 (3.01-4.38)	12.17 (9.8-14.21)	2.58 (2.13-3.12)	9.47 (7.13-11.49)	1.19 (0.98-1.43)	1.93 (-0.34-3.98)
Mood	Inflammatory bowel disease	Males	2.98 (2.67-3.32)	9.21 (8.18-10.24)	2.13 (1.91-2.39)	6.66 (5.56-7.7)	1.4 (1.26-1.56)	1.87 (0.78-2.89)
Neurotic	Inflammatory bowel disease	Males	3.19 (2.84-3.57)	9.64 (8.36-10.77)	2.28 (2.02-2.57)	6.92 (5.6-8.05)	1.3 (1.15-1.46)	1.77 (0.48-2.86)
Eating	Inflammatory bowel disease	Males	NA	NA	NA	NA	NA	NA

© 2022 Momen NC et al. *JAMA Psychiatry*.

Mental disorder	General medical condition	Sex	Compared to those with neither MD nor GMC		Compared to those with GMC only		Compared to those with MD only	
			MRR	LYLs	MRR	LYLs	MRR	LYLs
Personality	Inflammatory bowel disease	Males	3.11 (2.67-3.62)	9.51 (6.85-11.11)	2.21 (1.9-2.58)	6.83 (4.15-8.44)	1.23 (1.05-1.43)	1.24 (-1.46-2.87)
Intellectual	Inflammatory bowel disease	Males	4.39 (2.76-6.96)	NA	3.13 (1.97-4.97)	NA	1.26 (0.79-2.01)	NA
Developmental	Inflammatory bowel disease	Males	9.71 (4.63-20.36)	NA	6.93 (3.3-14.54)	NA	4.16 (1.96-8.82)	NA
Behavioral	Inflammatory bowel disease	Males	2.74 (1.55-4.82)	NA	1.95 (1.11-3.44)	NA	0.9 (0.51-1.59)	NA
Organic	Inflammatory bowel disease	Females	3.73 (3.42-4.07)	7.33 (6.69-7.98)	2.38 (2.18-2.61)	5.27 (4.66-5.87)	1.38 (1.26-1.5)	1.15 (0.63-1.64)
Substance Use	Inflammatory bowel disease	Females	4.59 (4.12-5.11)	12.94 (11.66-14.02)	3.01 (2.69-3.35)	9.26 (7.96-10.32)	1.35 (1.21-1.5)	2.17 (0.91-3.24)
Schizophrenia	Inflammatory bowel disease	Females	3.76 (3.27-4.32)	11.24 (9.83-12.66)	2.45 (2.13-2.82)	7.67 (6.23-9.07)	1.64 (1.42-1.88)	3.02 (1.57-4.42)
Mood	Inflammatory bowel disease	Females	2.45 (2.27-2.65)	8.27 (7.32-9.12)	1.6 (1.47-1.73)	4.7 (3.78-5.56)	1.36 (1.26-1.47)	2.42 (1.5-3.29)
Neurotic	Inflammatory bowel disease	Females	2.73 (2.51-2.98)	8.8 (7.77-9.79)	1.79 (1.63-1.96)	4.97 (3.89-6.01)	1.41 (1.3-1.54)	2.73 (1.66-3.71)
Eating	Inflammatory bowel disease	Females	5.8 (3.65-9.2)	NA	3.77 (2.38-5.99)	NA	2.29 (1.43-3.67)	NA
Personality	Inflammatory bowel disease	Females	2.95 (2.65-3.29)	9.47 (8.21-10.59)	1.93 (1.72-2.16)	5.64 (4.34-6.82)	1.45 (1.3-1.62)	2.83 (1.57-4.01)
Intellectual	Inflammatory bowel disease	Females	4.3 (2.83-6.54)	NA	2.8 (1.84-4.25)	NA	1.12 (0.74-1.71)	NA
Developmental	Inflammatory bowel disease	Females	NA	NA	NA	NA	NA	NA
Behavioral	Inflammatory bowel disease	Females	2.8 (1.55-5.06)	NA	1.82 (1.01-3.29)	NA	1.2 (0.66-2.18)	NA
Organic	Diverticular disease of the intestine	Persons	2.68 (2.6-2.76)	4.29 (4.14-4.44)	2.09 (2.02-2.15)	3.59 (3.43-3.74)	0.92 (0.9-0.95)	-0.16 (-0.31--0.03)
Substance Use	Diverticular disease of the intestine	Persons	3.18 (3.04-3.34)	8.74 (8.36-9.12)	2.53 (2.42-2.66)	7.11 (6.73-7.5)	0.8 (0.76-0.84)	0.82 (0.43-1.21)
Schizophrenia	Diverticular disease of the intestine	Persons	2.39 (2.24-2.55)	6.66 (6.1-7.19)	1.9 (1.78-2.03)	5.24 (4.69-5.76)	0.91 (0.85-0.97)	0.92 (0.4-1.45)
Mood	Diverticular disease of the intestine	Persons	1.85 (1.79-1.91)	4.38 (4.14-4.64)	1.47 (1.43-1.52)	3.1 (2.84-3.37)	0.95 (0.92-0.98)	0.52 (0.27-0.78)

© 2022 Momen NC et al. *JAMA Psychiatry*.

Mental disorder	General medical condition	Sex	Compared to those with neither MD nor GMC		Compared to those with GMC only		Compared to those with MD only	
			MRR	LYLs	MRR	LYLs	MRR	LYLs
Neurotic	Diverticular disease of the intestine	Persons	1.95 (1.88-2.03)	5.04 (4.73-5.37)	1.56 (1.5-1.62)	3.49 (3.17-3.82)	0.9 (0.87-0.94)	0.6 (0.27-0.94)
Eating	Diverticular disease of the intestine	Persons	3.5 (2.03-6.03)	NA	2.81 (1.63-4.83)	NA	1.32 (0.76-2.28)	NA
Personality	Diverticular disease of the intestine	Persons	2 (1.9-2.1)	5.1 (4.66-5.51)	1.6 (1.52-1.68)	3.55 (3.1-3.94)	0.88 (0.84-0.93)	0.53 (0.09-0.94)
Intellectual	Diverticular disease of the intestine	Persons	4.05 (3.23-5.07)	NA	3.24 (2.59-4.06)	NA	1.11 (0.89-1.4)	NA
Developmental	Diverticular disease of the intestine	Persons	NA	NA	NA	NA	NA	NA
Behavioral	Diverticular disease of the intestine	Persons	1.99 (1.5-2.64)	NA	1.59 (1.2-2.11)	NA	0.71 (0.54-0.95)	NA
Organic	Diverticular disease of the intestine	Males	2.72 (2.59-2.85)	4.56 (4.3-4.82)	2.12 (2.02-2.23)	3.8 (3.55-4.05)	0.86 (0.82-0.91)	-0.28 (-0.49--0.05)
Substance Use	Diverticular disease of the intestine	Males	3.64 (3.41-3.89)	9.4 (8.84-9.99)	2.89 (2.7-3.09)	7.68 (7.11-8.28)	0.84 (0.79-0.9)	0.95 (0.36-1.52)
Schizophrenia	Diverticular disease of the intestine	Males	2.6 (2.32-2.91)	7.43 (6.4-8.38)	2.07 (1.85-2.32)	5.84 (4.83-6.78)	0.84 (0.75-0.94)	0.84 (-0.16-1.71)
Mood	Diverticular disease of the intestine	Males	1.98 (1.88-2.1)	5.02 (4.59-5.46)	1.58 (1.5-1.68)	3.64 (3.2-4.09)	0.92 (0.87-0.97)	0.51 (0.07-0.95)
Neurotic	Diverticular disease of the intestine	Males	2.25 (2.1-2.4)	6.01 (5.45-6.57)	1.8 (1.68-1.93)	4.33 (3.77-4.92)	0.9 (0.84-0.97)	0.71 (0.14-1.26)
Eating	Diverticular disease of the intestine	Males	NA	NA	NA	NA	NA	NA
Personality	Diverticular disease of the intestine	Males	2.04 (1.87-2.24)	5.25 (4.45-5.99)	1.63 (1.49-1.79)	3.64 (2.85-4.38)	0.79 (0.73-0.87)	0.12 (-0.7-0.84)
Intellectual	Diverticular disease of the intestine	Males	3.72 (2.59-5.35)	NA	2.99 (2.08-4.31)	NA	1.07 (0.74-1.54)	NA
Developmental	Diverticular disease of the intestine	Males	NA	NA	NA	NA	NA	NA
Behavioral	Diverticular disease of the intestine	Males	2.52 (1.63-3.91)	NA	2.03 (1.31-3.15)	NA	0.83 (0.53-1.28)	NA
Organic	Diverticular disease of the intestine	Females	2.66 (2.56-2.75)	4.14 (3.95-4.33)	2.06 (1.98-2.14)	3.47 (3.27-3.66)	0.96 (0.93-1)	-0.1 (-0.28-0.07)
Substance Use	Diverticular disease of the intestine	Females	2.83 (2.65-3.02)	8.02 (7.48-8.55)	2.26 (2.11-2.41)	6.49 (5.97-7.02)	0.81 (0.76-0.87)	0.68 (0.14-1.19)

© 2022 Momen NC et al. *JAMA Psychiatry*.

Mental disorder	General medical condition	Sex	Compared to those with neither MD nor GMC		Compared to those with GMC only		Compared to those with MD only	
			MRR	LYLs	MRR	LYLs	MRR	LYLs
Schizophrenia	Diverticular disease of the intestine	Females	2.29 (2.12-2.48)	6.25 (5.61-6.91)	1.82 (1.68-1.97)	4.91 (4.26-5.55)	0.98 (0.91-1.07)	0.96 (0.35-1.61)
Mood	Diverticular disease of the intestine	Females	1.79 (1.73-1.86)	4.06 (3.78-4.35)	1.42 (1.37-1.48)	2.84 (2.54-3.15)	0.98 (0.95-1.02)	0.53 (0.24-0.84)
Neurotic	Diverticular disease of the intestine	Females	1.83 (1.75-1.92)	4.53 (4.13-4.95)	1.46 (1.39-1.53)	3.04 (2.62-3.47)	0.93 (0.89-0.98)	0.54 (0.11-0.95)
Eating	Diverticular disease of the intestine	Females	3.1 (1.72-5.6)	NA	2.48 (1.37-4.48)	NA	1.21 (0.66-2.19)	NA
Personality	Diverticular disease of the intestine	Females	1.98 (1.86-2.1)	5.03 (4.49-5.51)	1.58 (1.48-1.68)	3.5 (2.95-3.99)	0.96 (0.9-1.02)	0.73 (0.16-1.22)
Intellectual	Diverticular disease of the intestine	Females	4.28 (3.21-5.69)	NA	3.42 (2.57-4.55)	NA	1.11 (0.83-1.49)	NA
Developmental	Diverticular disease of the intestine	Females	NA	NA	NA	NA	NA	NA
Behavioral	Diverticular disease of the intestine	Females	1.72 (1.19-2.5)	NA	1.38 (0.95-2)	NA	0.72 (0.49-1.05)	NA
Organic	Urogenital system	Persons	3.79 (3.72-3.85)	5.31 (5.22-5.4)	2.32 (2.28-2.36)	3.79 (3.71-3.88)	1.27 (1.25-1.29)	0.59 (0.51-0.67)
Substance Use	Urogenital system	Persons	4.54 (4.43-4.67)	10.19 (9.96-10.43)	2.79 (2.72-2.87)	6.41 (6.19-6.62)	1.1 (1.07-1.14)	2.52 (2.3-2.74)
Schizophrenia	Urogenital system	Persons	3.69 (3.56-3.83)	9.38 (9.06-9.71)	2.3 (2.22-2.39)	5.02 (4.72-5.32)	1.4 (1.35-1.46)	3.31 (2.99-3.62)
Mood	Urogenital system	Persons	2.72 (2.66-2.77)	6.48 (6.32-6.65)	1.7 (1.66-1.73)	2.95 (2.8-3.11)	1.41 (1.38-1.44)	2.51 (2.34-2.68)
Neurotic	Urogenital system	Persons	3.07 (2.99-3.15)	8.17 (7.93-8.38)	1.92 (1.87-1.97)	3.53 (3.31-3.75)	1.44 (1.4-1.48)	3.53 (3.28-3.76)
Eating	Urogenital system	Persons	9.78 (7.28-13.14)	NA	6.11 (4.55-8.22)	NA	4.08 (3-5.54)	NA
Personality	Urogenital system	Persons	3.04 (2.94-3.14)	8.15 (7.86-8.44)	1.9 (1.84-1.96)	3.53 (3.26-3.79)	1.35 (1.3-1.39)	3.34 (3.02-3.63)
Intellectual	Urogenital system	Persons	4.28 (3.87-4.73)	13.07 (11.74-14.28)	2.67 (2.42-2.96)	7.77 (6.53-8.84)	1.13 (1.02-1.26)	4.67 (3.37-5.81)
Developmental	Urogenital system	Persons	4.05 (2.78-5.9)	NA	2.53 (1.73-3.69)	NA	1.54 (1.04-2.29)	NA
Behavioral	Urogenital system	Persons	4.26 (3.67-4.95)	17.05 (15.02-19.08)	2.66 (2.29-3.09)	7.41 (5.33-9.42)	1.55 (1.33-1.82)	9.61 (7.49-11.75)
Organic	Urogenital system	Males	3.48 (3.41-3.54)	5.02 (4.93-5.11)	2.44 (2.39-2.48)	4.06 (3.97-4.15)	1 (0.97-1.02)	0.32 (0.23-0.4)
Substance Use	Urogenital system	Males	4.08 (3.96-4.2)	9.49 (9.26-9.74)	2.85 (2.77-2.94)	6.89 (6.67-7.13)	0.85 (0.83-0.88)	1.83 (1.6-2.08)
Schizophrenia	Urogenital system	Males	3.15 (3.02-3.28)	8.36 (7.98-8.7)	2.24 (2.15-2.34)	5.79 (5.43-6.12)	0.93 (0.89-0.98)	2.19 (1.82-2.55)
Mood	Urogenital system	Males	2.33 (2.28-2.39)	5.45 (5.29-5.63)	1.66 (1.62-1.7)	3.52 (3.36-3.7)	0.99 (0.96-1.02)	1.4 (1.23-1.6)
Neurotic	Urogenital system	Males	2.61 (2.53-2.68)	6.75 (6.5-7.01)	1.86 (1.81-1.91)	4.13 (3.87-4.37)	0.96 (0.93-1)	2 (1.73-2.28)

© 2022 Momen NC et al. *JAMA Psychiatry*.

Mental disorder	General medical condition	Sex	Compared to those with neither MD nor GMC		Compared to those with GMC only		Compared to those with MD only	
			MRR	LYLs	MRR	LYLs	MRR	LYLs
Eating	Urogenital system	Males	5.56 (2.9-10.7)	NA	4 (2.08-7.69)	NA	1.49 (0.74-3)	NA
Personality	Urogenital system	Males	2.57 (2.48-2.66)	6.74 (6.43-7.05)	1.83 (1.77-1.9)	4.15 (3.84-4.45)	0.92 (0.88-0.95)	1.82 (1.5-2.16)
Intellectual	Urogenital system	Males	3.59 (3.21-4.01)	11.53 (10.09-12.8)	2.58 (2.31-2.88)	8.03 (6.66-9.19)	0.95 (0.84-1.07)	3.35 (1.95-4.54)
Developmental	Urogenital system	Males	3.57 (2.37-5.37)	NA	2.56 (1.7-3.86)	NA	1.46 (0.95-2.24)	NA
Behavioral	Urogenital system	Males	3.38 (2.85-4.01)	14.05 (11.67-16.46)	2.43 (2.05-2.88)	7.61 (5.22-10.04)	1.06 (0.88-1.27)	6.57 (4.03-9.17)
Organic	Urogenital system	Females	6.35 (6.04-6.67)	7.69 (7.38-7.99)	1.39 (1.32-1.46)	1.56 (1.35-1.76)	2.24 (2.13-2.36)	2.84 (2.62-3.05)
Substance Use	Urogenital system	Females	9.41 (8.75-10.12)	15.38 (14.77-16.01)	2.21 (2.05-2.38)	2.76 (2.26-3.28)	2.78 (2.58-2.99)	7.63 (7.12-8.15)
Schizophrenia	Urogenital system	Females	7.02 (6.49-7.6)	13.49 (12.78-14.19)	1.65 (1.52-1.78)	1.94 (1.35-2.51)	3.04 (2.8-3.29)	7.79 (7.18-8.42)
Mood	Urogenital system	Females	5.23 (5.01-5.47)	10.73 (10.32-11.13)	1.2 (1.15-1.26)	0.6 (0.25-0.95)	2.89 (2.76-3.02)	7.09 (6.72-7.46)
Neurotic	Urogenital system	Females	6.19 (5.86-6.53)	13.88 (13.37-14.43)	1.44 (1.37-1.53)	1.12 (0.62-1.63)	3.21 (3.04-3.4)	9.71 (9.22-10.21)
Eating	Urogenital system	Females	12.38 (8.89-17.24)	NA	2.92 (2.09-4.06)	NA	5.15 (3.65-7.25)	NA
Personality	Urogenital system	Females	6.32 (5.9-6.77)	13.62 (12.98-14.26)	1.48 (1.38-1.59)	1.12 (0.57-1.7)	3.13 (2.92-3.36)	9.21 (8.62-9.84)
Intellectual	Urogenital system	Females	13.71 (10.73-17.52)	NA	3.23 (2.53-4.13)	NA	3.64 (2.83-4.67)	NA
Developmental	Urogenital system	Females	NA	NA	NA	NA	NA	NA
Behavioral	Urogenital system	Females	14.8 (10.81-20.26)	NA	3.49 (2.55-4.77)	NA	6.62 (4.79-9.16)	NA
Organic	Chronic kidney disease	Persons	6.82 (6.6-7.05)	7.65 (7.46-7.83)	1.51 (1.46-1.57)	1.61 (1.49-1.72)	2.29 (2.21-2.37)	2.64 (2.5-2.76)
Substance Use	Chronic kidney disease	Persons	10.16 (9.73-10.61)	15.33 (14.97-15.73)	2.37 (2.27-2.48)	3.07 (2.79-3.37)	2.57 (2.46-2.69)	7.4 (7.12-7.72)
Schizophrenia	Chronic kidney disease	Persons	7.21 (6.82-7.63)	13.76 (13.23-14.3)	1.69 (1.6-1.79)	2.03 (1.61-2.43)	2.75 (2.6-2.91)	7.63 (7.18-8.08)
Mood	Chronic kidney disease	Persons	5.55 (5.38-5.73)	10.68 (10.38-10.97)	1.29 (1.24-1.33)	0.9 (0.67-1.15)	2.87 (2.78-2.97)	6.83 (6.58-7.1)
Neurotic	Chronic kidney disease	Persons	6.42 (6.18-6.68)	13.66 (13.29-14.03)	1.5 (1.45-1.57)	1.3 (0.95-1.61)	3 (2.88-3.13)	9.11 (8.76-9.44)
Eating	Chronic kidney disease	Persons	12.34 (8.94-17.03)	NA	2.91 (2.11-4.02)	NA	4.83 (3.47-6.74)	NA
Personality	Chronic kidney disease	Persons	6.64 (6.32-6.98)	13.36 (12.9-13.81)	1.56 (1.48-1.64)	1.34 (0.94-1.71)	2.96 (2.81-3.12)	8.76 (8.34-9.16)
Intellectual	Chronic kidney disease	Persons	10.2 (8.64-12.04)	NA	2.41 (2.04-2.84)	NA	2.78 (2.35-3.3)	NA
Developmental	Chronic kidney disease	Persons	11.61 (6.74-19.99)	NA	2.74 (1.59-4.72)	NA	4.52 (2.6-7.86)	NA
Behavioral	Chronic kidney disease	Persons	13.36 (10.89-16.39)	NA	3.16 (2.57-3.87)	NA	4.93 (3.99-6.08)	NA
Organic	Chronic kidney disease	Males	7.14 (6.83-7.47)	7.61 (7.36-7.87)	1.63 (1.56-1.71)	1.73 (1.57-1.89)	2.2 (2.1-2.3)	2.43 (2.26-2.6)
Substance Use	Chronic kidney disease	Males	10.49 (9.94-11.07)	15.27 (14.78-15.72)	2.49 (2.36-2.64)	3.34 (3-3.69)	2.4 (2.27-2.53)	7.23 (6.85-7.61)

© 2022 Momen NC et al. *JAMA Psychiatry*.

Mental disorder	General medical condition	Sex	Compared to those with neither MD nor GMC		Compared to those with GMC only		Compared to those with MD only	
			MRR	LYLs	MRR	LYLs	MRR	LYLs
Schizophrenia	Chronic kidney disease	Males	7.32 (6.76-7.93)	14.01 (13.3-14.81)	1.76 (1.62-1.91)	2.3 (1.72-2.87)	2.34 (2.15-2.54)	7.4 (6.79-8.07)
Mood	Chronic kidney disease	Males	5.75 (5.49-6.03)	10.49 (10.12-10.87)	1.37 (1.3-1.44)	1.37 (1.07-1.66)	2.65 (2.52-2.78)	6.37 (6.04-6.72)
Neurotic	Chronic kidney disease	Males	6.49 (6.13-6.87)	13.29 (12.74-13.78)	1.56 (1.47-1.65)	1.6 (1.16-2)	2.59 (2.44-2.75)	8.29 (7.81-8.72)
Eating	Chronic kidney disease	Males	NA	NA	NA	NA	NA	NA
Personality	Chronic kidney disease	Males	6.7 (6.23-7.2)	12.89 (12.28-13.54)	1.61 (1.5-1.74)	1.73 (1.21-2.19)	2.6 (2.41-2.8)	8.01 (7.44-8.56)
Intellectual	Chronic kidney disease	Males	8.33 (6.65-10.43)	NA	2.02 (1.61-2.53)	NA	2.34 (1.86-2.95)	NA
Developmental	Chronic kidney disease	Males	13.51 (7.03-25.96)	NA	3.28 (1.71-6.3)	NA	5.61 (2.89-10.91)	NA
Behavioral	Chronic kidney disease	Males	12.39 (9.47-16.22)	NA	3.01 (2.3-3.94)	NA	4.06 (3.08-5.35)	NA
Organic	Chronic kidney disease	Females	6.45 (6.14-6.78)	7.71 (7.4-7.99)	1.36 (1.29-1.43)	1.47 (1.26-1.66)	2.29 (2.18-2.41)	2.88 (2.65-3.08)
Substance Use	Chronic kidney disease	Females	9.64 (8.96-10.37)	15.43 (14.78-16.04)	2.18 (2.02-2.34)	2.6 (2.12-3.1)	2.83 (2.63-3.05)	7.7 (7.19-8.22)
Schizophrenia	Chronic kidney disease	Females	7.11 (6.57-7.69)	13.53 (12.78-14.22)	1.6 (1.48-1.73)	1.78 (1.2-2.32)	3.07 (2.84-3.33)	7.84 (7.2-8.48)
Mood	Chronic kidney disease	Females	5.39 (5.16-5.63)	10.83 (10.44-11.22)	1.19 (1.13-1.24)	0.53 (0.18-0.88)	2.98 (2.85-3.11)	7.2 (6.84-7.57)
Neurotic	Chronic kidney disease	Females	6.36 (6.02-6.72)	13.98 (13.43-14.52)	1.43 (1.35-1.51)	1.04 (0.52-1.53)	3.29 (3.11-3.48)	9.82 (9.27-10.35)
Eating	Chronic kidney disease	Females	12.62 (9.06-17.58)	NA	2.86 (2.05-3.98)	NA	5.14 (3.65-7.24)	NA
Personality	Chronic kidney disease	Females	6.58 (6.14-7.05)	13.73 (13.09-14.37)	1.48 (1.38-1.59)	1.04 (0.45-1.56)	3.25 (3.03-3.49)	9.33 (8.75-9.93)
Intellectual	Chronic kidney disease	Females	13.88 (10.86-17.73)	NA	3.14 (2.46-4.01)	NA	3.66 (2.85-4.7)	NA
Developmental	Chronic kidney disease	Females	NA	NA	NA	NA	NA	NA
Behavioral	Chronic kidney disease	Females	14.94 (10.91-20.45)	NA	3.38 (2.47-4.63)	NA	6.61 (4.78-9.14)	NA
Organic	Prostate disorders	Persons	3.2 (3.14-3.26)	4.68 (4.59-4.77)	2.63 (2.58-2.68)	4.22 (4.13-4.31)	0.92 (0.9-0.95)	0.09 (0-0.17)
Substance Use	Prostate disorders	Persons	3.32 (3.22-3.43)	7.98 (7.74-8.23)	2.72 (2.64-2.81)	6.86 (6.61-7.12)	0.72 (0.7-0.74)	0.56 (0.3-0.81)
Schizophrenia	Prostate disorders	Persons	2.65 (2.53-2.77)	6.85 (6.5-7.2)	2.19 (2.1-2.3)	5.77 (5.42-6.11)	0.81 (0.77-0.85)	0.92 (0.55-1.27)
Mood	Prostate disorders	Persons	2.05 (2-2.1)	4.52 (4.35-4.69)	1.7 (1.66-1.74)	3.67 (3.49-3.85)	0.88 (0.85-0.9)	0.57 (0.39-0.77)
Neurotic	Prostate disorders	Persons	2.21 (2.14-2.28)	5.42 (5.18-5.66)	1.83 (1.77-1.89)	4.3 (4.06-4.55)	0.84 (0.81-0.87)	0.82 (0.56-1.1)
Eating	Prostate disorders	Persons	4.71 (2.25-9.89)	NA	3.94 (1.88-8.26)	NA	1.36 (0.62-2.98)	NA
Personality	Prostate disorders	Persons	2.15 (2.07-2.24)	5.48 (5.14-5.82)	1.79 (1.72-1.86)	4.35 (4-4.69)	0.79 (0.76-0.83)	0.66 (0.32-1.01)
Intellectual	Prostate disorders	Persons	3.04 (2.69-3.43)	9.3 (8.06-10.46)	2.53 (2.24-2.86)	7.95 (6.71-9.02)	0.84 (0.74-0.96)	1.46 (0.19-2.62)
Developmental	Prostate disorders	Persons	2.49 (1.52-4.06)	NA	2.08 (1.27-3.39)	NA	1.11 (0.67-1.84)	NA

© 2022 Momen NC et al. *JAMA Psychiatry*.

Mental disorder	General medical condition	Sex	Compared to those with neither MD nor GMC		Compared to those with GMC only		Compared to those with MD only	
			MRR	LYLs	MRR	LYLs	MRR	LYLs
Behavioral	Prostate disorders	Persons	2.55 (2.09-3.11)	9.94 (7.56-12.46)	2.13 (1.75-2.6)	7.7 (5.35-10.18)	0.86 (0.7-1.06)	3.06 (0.58-5.61)
Organic	Prostate disorders	Males	3.2 (3.14-3.26)	4.68 (4.59-4.77)	2.63 (2.58-2.68)	4.22 (4.13-4.31)	0.92 (0.9-0.95)	0.09 (0-0.17)
Substance Use	Prostate disorders	Males	3.32 (3.22-3.43)	7.98 (7.74-8.23)	2.72 (2.64-2.81)	6.86 (6.61-7.12)	0.72 (0.7-0.74)	0.56 (0.3-0.81)
Schizophrenia	Prostate disorders	Males	2.65 (2.53-2.77)	6.85 (6.5-7.2)	2.19 (2.1-2.3)	5.77 (5.42-6.11)	0.81 (0.77-0.85)	0.92 (0.55-1.27)
Mood	Prostate disorders	Males	2.05 (2-2.1)	4.52 (4.35-4.69)	1.7 (1.66-1.74)	3.67 (3.49-3.85)	0.88 (0.85-0.9)	0.57 (0.39-0.77)
Neurotic	Prostate disorders	Males	2.21 (2.14-2.28)	5.42 (5.18-5.66)	1.83 (1.77-1.89)	4.3 (4.06-4.55)	0.84 (0.81-0.87)	0.82 (0.56-1.1)
Eating	Prostate disorders	Males	4.71 (2.25-9.89)	NA	3.94 (1.88-8.26)	NA	1.36 (0.62-2.98)	NA
Personality	Prostate disorders	Males	2.15 (2.07-2.24)	5.48 (5.14-5.82)	1.79 (1.72-1.86)	4.35 (4-4.69)	0.79 (0.76-0.83)	0.66 (0.32-1.01)
Intellectual	Prostate disorders	Males	3.04 (2.69-3.43)	9.3 (8.06-10.46)	2.53 (2.24-2.86)	7.95 (6.71-9.02)	0.84 (0.74-0.96)	1.46 (0.19-2.62)
Developmental	Prostate disorders	Males	2.49 (1.52-4.06)	NA	2.08 (1.27-3.39)	NA	1.11 (0.67-1.84)	NA
Behavioral	Prostate disorders	Males	2.55 (2.09-3.11)	9.94 (7.56-12.46)	2.13 (1.75-2.6)	7.7 (5.35-10.18)	0.86 (0.7-1.06)	3.06 (0.58-5.61)
Organic	Musculoskeletal system	Persons	6.92 (6.85-6.98)	5.93 (5.88-5.97)	2.3 (2.29-2.32)	4.3 (4.26-4.35)	1.79 (1.76-1.82)	0.69 (0.63-0.74)
Substance Use	Musculoskeletal system	Persons	7.92 (7.83-8.01)	13 (12.89-13.12)	2.68 (2.65-2.71)	8.49 (8.37-8.6)	1.6 (1.57-1.63)	2.11 (1.98-2.25)
Schizophrenia	Musculoskeletal system	Persons	6.37 (6.27-6.47)	11.09 (10.93-11.25)	2.12 (2.09-2.15)	7.17 (7.01-7.33)	1.91 (1.86-1.96)	3.25 (3.06-3.46)
Mood	Musculoskeletal system	Persons	4.65 (4.61-4.7)	7.61 (7.52-7.71)	1.55 (1.54-1.56)	4.07 (3.97-4.17)	2.03 (1.99-2.07)	2.22 (2.09-2.34)
Neurotic	Musculoskeletal system	Persons	4.97 (4.91-5.03)	8.76 (8.64-8.87)	1.67 (1.65-1.69)	4.46 (4.34-4.58)	2.15 (2.1-2.2)	2.63 (2.5-2.78)
Eating	Musculoskeletal system	Persons	6.94 (6.28-7.66)	10.66 (9.08-11.97)	2.32 (2.11-2.57)	5.77 (4.17-7.07)	2.92 (2.47-3.46)	2.64 (0.69-4.66)
Personality	Musculoskeletal system	Persons	5.11 (5.05-5.18)	9.35 (9.2-9.49)	1.72 (1.7-1.74)	5.05 (4.9-5.2)	2.13 (2.07-2.19)	2.71 (2.53-2.89)
Intellectual	Musculoskeletal system	Persons	8.26 (7.91-8.62)	14.05 (13.55-14.56)	2.77 (2.65-2.89)	9.24 (8.74-9.75)	1.96 (1.82-2.11)	3.26 (2.69-3.84)
Developmental	Musculoskeletal system	Persons	7.35 (6.27-8.6)	13.12 (10.91-14.89)	2.46 (2.1-2.88)	6.74 (4.53-8.53)	2.99 (2.43-3.69)	4.74 (2.3-7.03)
Behavioral	Musculoskeletal system	Persons	6.18 (5.83-6.56)	11.71 (11.01-12.48)	2.07 (1.95-2.2)	5.8 (5.1-6.55)	2.39 (2.17-2.63)	3.31 (2.25-4.37)
Organic	Musculoskeletal system	Males	6.99 (6.9-7.09)	6.8 (6.72-6.88)	2.36 (2.33-2.39)	4.55 (4.47-4.62)	1.76 (1.72-1.8)	0.86 (0.77-0.95)
Substance Use	Musculoskeletal system	Males	8.21 (8.09-8.34)	13.62 (13.48-13.76)	2.81 (2.77-2.85)	8.38 (8.23-8.53)	1.62 (1.58-1.66)	2.38 (2.21-2.57)
Schizophrenia	Musculoskeletal system	Males	7.02 (6.85-7.2)	12.91 (12.65-13.16)	2.37 (2.31-2.43)	7.81 (7.55-8.07)	1.92 (1.85-1.99)	4.01 (3.7-4.33)
Mood	Musculoskeletal system	Males	4.82 (4.74-4.9)	8.8 (8.65-8.96)	1.63 (1.6-1.65)	4.33 (4.17-4.49)	1.95 (1.9-2.01)	2.63 (2.46-2.83)
Neurotic	Musculoskeletal system	Males	5.3 (5.2-5.39)	10.17 (9.99-10.36)	1.81 (1.78-1.84)	4.85 (4.66-5.04)	2.03 (1.97-2.09)	3.08 (2.87-3.3)
Eating	Musculoskeletal system	Males	8.72 (6.26-12.15)	NA	2.96 (2.13-4.13)	NA	2.48 (1.53-4.02)	NA

© 2022 Momen NC et al. *JAMA Psychiatry*.

Mental disorder	General medical condition	Sex	Compared to those with neither MD nor GMC		Compared to those with GMC only		Compared to those with MD only	
			MRR	LYLs	MRR	LYLs	MRR	LYLs
Personality	Musculoskeletal system	Males	5.42 (5.31-5.53)	10.77 (10.55-10.98)	1.85 (1.81-1.89)	5.52 (5.3-5.73)	2.05 (1.98-2.13)	3.29 (2.99-3.54)
Intellectual	Musculoskeletal system	Males	7.69 (7.24-8.17)	13.93 (13.21-14.63)	2.61 (2.46-2.78)	8.21 (7.49-8.91)	2.06 (1.87-2.27)	3.84 (3.05-4.68)
Developmental	Musculoskeletal system	Males	6.74 (5.57-8.16)	12.33 (9.91-14.55)	2.29 (1.89-2.77)	4.96 (2.59-7.2)	3.01 (2.35-3.85)	5.7 (2.73-8.08)
Behavioral	Musculoskeletal system	Males	6.71 (6.2-7.26)	12.99 (11.96-14.02)	2.28 (2.11-2.47)	6 (4.95-7)	2.47 (2.19-2.77)	4.1 (2.74-5.48)
Organic	Musculoskeletal system	Females	6.93 (6.85-7.01)	5.42 (5.36-5.48)	2.27 (2.25-2.29)	4.16 (4.11-4.22)	1.85 (1.8-1.9)	0.58 (0.52-0.65)
Substance Use	Musculoskeletal system	Females	7.56 (7.43-7.7)	12.19 (12.01-12.36)	2.52 (2.48-2.57)	8.64 (8.46-8.81)	1.65 (1.59-1.72)	1.75 (1.53-1.96)
Schizophrenia	Musculoskeletal system	Females	6.07 (5.95-6.19)	9.89 (9.7-10.11)	1.99 (1.95-2.02)	6.74 (6.55-6.95)	2.06 (1.98-2.14)	2.76 (2.52-3.03)
Mood	Musculoskeletal system	Females	4.61 (4.55-4.66)	7.03 (6.91-7.15)	1.51 (1.49-1.53)	3.94 (3.82-4.07)	2.16 (2.1-2.22)	2.01 (1.87-2.17)
Neurotic	Musculoskeletal system	Females	4.81 (4.74-4.89)	8.01 (7.87-8.16)	1.59 (1.57-1.62)	4.25 (4.09-4.41)	2.4 (2.32-2.48)	2.4 (2.21-2.59)
Eating	Musculoskeletal system	Females	6.89 (6.22-7.65)	10.56 (8.99-11.97)	2.27 (2.04-2.52)	5.76 (4.18-7.15)	3.03 (2.53-3.63)	2.64 (0.66-4.8)
Personality	Musculoskeletal system	Females	4.95 (4.87-5.04)	8.6 (8.41-8.79)	1.64 (1.61-1.67)	4.81 (4.62-5.01)	2.34 (2.24-2.44)	2.4 (2.17-2.65)
Intellectual	Musculoskeletal system	Females	8.97 (8.44-9.53)	14.16 (13.41-14.86)	2.95 (2.77-3.13)	10.18 (9.46-10.86)	1.72 (1.53-1.92)	2.73 (1.83-3.59)
Developmental	Musculoskeletal system	Females	9.05 (6.82-12.02)	14.18 (10.52-17.07)	2.98 (2.24-3.95)	9.14 (5.49-12.01)	2.6 (1.74-3.88)	3.44 (-0.66-8.11)
Behavioral	Musculoskeletal system	Females	5.66 (5.18-6.18)	10.41 (9.33-11.53)	1.86 (1.7-2.03)	5.6 (4.54-6.71)	2.57 (2.13-3.09)	2.5 (1.09-3.96)
Organic	Connective tissue disorders	Persons	3.22 (3.11-3.33)	5.59 (5.37-5.8)	2.11 (2.04-2.19)	4.03 (3.83-4.24)	1.11 (1.07-1.15)	0.4 (0.23-0.58)
Substance Use	Connective tissue disorders	Persons	3.78 (3.58-4)	11.33 (10.77-11.86)	2.53 (2.4-2.68)	7.94 (7.37-8.44)	0.96 (0.9-1.01)	1.49 (0.91-1.99)
Schizophrenia	Connective tissue disorders	Persons	2.81 (2.62-3.02)	9.46 (8.61-10.22)	1.89 (1.76-2.04)	6.17 (5.33-6.95)	1.07 (0.99-1.15)	1.68 (0.88-2.44)
Mood	Connective tissue disorders	Persons	2.28 (2.2-2.36)	7.11 (6.73-7.51)	1.53 (1.48-1.59)	4.11 (3.71-4.51)	1.18 (1.14-1.22)	1.95 (1.56-2.34)
Neurotic	Connective tissue disorders	Persons	2.4 (2.3-2.51)	7.68 (7.2-8.13)	1.62 (1.55-1.69)	4.17 (3.69-4.62)	1.12 (1.07-1.16)	1.85 (1.38-2.29)
Eating	Connective tissue disorders	Persons	1.98 (1.21-3.23)	NA	1.34 (0.82-2.2)	NA	0.73 (0.44-1.2)	NA
Personality	Connective tissue disorders	Persons	2.44 (2.31-2.58)	7.47 (6.86-8.01)	1.65 (1.56-1.74)	4.05 (3.4-4.61)	1.08 (1.02-1.14)	1.35 (0.72-1.9)
Intellectual	Connective tissue disorders	Persons	5.4 (4.15-7.04)	NA	3.67 (2.82-4.78)	NA	1.48 (1.13-1.93)	NA
Developmental	Connective tissue disorders	Persons	NA	NA	NA	NA	NA	NA

© 2022 Momen NC et al. *JAMA Psychiatry*.

Mental disorder	General medical condition	Sex	Compared to those with neither MD nor GMC		Compared to those with GMC only		Compared to those with MD only	
			MRR	LYLs	MRR	LYLs	MRR	LYLs
Behavioral	Connective tissue disorders	Persons	3.24 (2.51-4.2)	11.53 (4.02-15.33)	2.21 (1.71-2.86)	6.71 (-0.81-10.48)	1.18 (0.91-1.53)	3.08 (-4.13-6.99)
Organic	Connective tissue disorders	Males	3.44 (3.23-3.67)	6.1 (5.73-6.53)	2.29 (2.14-2.45)	4.57 (4.23-4.96)	1.11 (1.04-1.18)	0.64 (0.35-0.98)
Substance Use	Connective tissue disorders	Males	4.39 (4.04-4.78)	12.3 (11.45-13.13)	2.95 (2.71-3.21)	8.56 (7.7-9.41)	1.02 (0.94-1.11)	2.11 (1.25-2.9)
Schizophrenia	Connective tissue disorders	Males	3.74 (3.24-4.32)	12.49 (10.89-14.07)	2.54 (2.2-2.94)	8.51 (6.91-10.08)	1.22 (1.05-1.41)	3.53 (1.98-5.1)
Mood	Connective tissue disorders	Males	2.47 (2.3-2.66)	8.4 (7.62-9.18)	1.68 (1.56-1.81)	5.15 (4.37-5.95)	1.15 (1.07-1.24)	2.63 (1.89-3.41)
Neurotic	Connective tissue disorders	Males	2.88 (2.65-3.13)	9.66 (8.63-10.55)	1.96 (1.8-2.14)	5.58 (4.57-6.53)	1.16 (1.07-1.27)	2.71 (1.71-3.58)
Eating	Connective tissue disorders	Males	NA	NA	NA	NA	NA	NA
Personality	Connective tissue disorders	Males	2.87 (2.58-3.2)	9.27 (8-10.3)	1.96 (1.75-2.18)	5.48 (4.19-6.51)	1.13 (1.01-1.26)	2.45 (1.17-3.45)
Intellectual	Connective tissue disorders	Males	5.17 (3.41-7.86)	NA	3.54 (2.33-5.38)	NA	1.49 (0.98-2.26)	NA
Developmental	Connective tissue disorders	Males	NA	NA	NA	NA	NA	NA
Behavioral	Connective tissue disorders	Males	3.42 (2.23-5.24)	NA	2.34 (1.53-3.59)	NA	1.12 (0.73-1.73)	NA
Organic	Connective tissue disorders	Females	3.12 (3-3.25)	5.39 (5.14-5.64)	2.04 (1.96-2.13)	3.83 (3.58-4.08)	1.13 (1.09-1.18)	0.31 (0.1-0.53)
Substance Use	Connective tissue disorders	Females	3.41 (3.17-3.67)	10.65 (9.87-11.37)	2.29 (2.13-2.46)	7.5 (6.74-8.22)	0.98 (0.91-1.06)	1.05 (0.29-1.76)
Schizophrenia	Connective tissue disorders	Females	2.59 (2.38-2.82)	8.46 (7.55-9.28)	1.74 (1.6-1.89)	5.4 (4.5-6.24)	1.11 (1.02-1.2)	1.07 (0.15-1.9)
Mood	Connective tissue disorders	Females	2.22 (2.13-2.31)	6.75 (6.3-7.18)	1.49 (1.43-1.55)	3.83 (3.37-4.29)	1.22 (1.17-1.27)	1.76 (1.32-2.2)
Neurotic	Connective tissue disorders	Females	2.27 (2.16-2.38)	7.13 (6.59-7.68)	1.52 (1.45-1.6)	3.77 (3.2-4.32)	1.16 (1.1-1.22)	1.61 (1.06-2.16)
Eating	Connective tissue disorders	Females	1.81 (1.07-3.05)	NA	1.23 (0.73-2.07)	NA	0.69 (0.4-1.17)	NA
Personality	Connective tissue disorders	Females	2.31 (2.16-2.46)	7.03 (6.3-7.68)	1.55 (1.45-1.66)	3.7 (2.93-4.37)	1.12 (1.05-1.2)	1.08 (0.38-1.75)

© 2022 Momen NC et al. *JAMA Psychiatry*.

Mental disorder	General medical condition	Sex	Compared to those with neither MD nor GMC		Compared to those with GMC only		Compared to those with MD only	
			MRR	LYLs	MRR	LYLs	MRR	LYLs
Intellectual	Connective tissue disorders	Females	5.57 (3.96-7.83)	NA	3.77 (2.68-5.3)	NA	1.44 (1.02-2.03)	NA
Developmental	Connective tissue disorders	Females	NA	NA	NA	NA	NA	NA
Behavioral	Connective tissue disorders	Females	3.16 (2.29-4.36)	11.56 (4.95-16.12)	2.14 (1.55-2.95)	7.59 (1.07-12.16)	1.35 (0.97-1.88)	3.66 (-3.03-8.31)
Organic	Osteoporosis	Persons	3.47 (3.4-3.53)	5.03 (4.94-5.13)	2.03 (1.99-2.07)	3.46 (3.37-3.55)	1.15 (1.12-1.17)	0.53 (0.45-0.62)
Substance Use	Osteoporosis	Persons	4.62 (4.49-4.76)	10.39 (10.16-10.63)	2.82 (2.73-2.9)	6.21 (5.98-6.44)	1.16 (1.12-1.19)	3.01 (2.77-3.26)
Schizophrenia	Osteoporosis	Persons	3.12 (2.99-3.25)	7.83 (7.48-8.15)	1.88 (1.8-1.96)	4.5 (4.17-4.82)	1.15 (1.1-1.2)	2.41 (2.06-2.75)
Mood	Osteoporosis	Persons	2.56 (2.51-2.61)	5.87 (5.72-6.02)	1.54 (1.51-1.57)	3.2 (3.05-3.35)	1.3 (1.27-1.33)	2.36 (2.2-2.51)
Neurotic	Osteoporosis	Persons	2.89 (2.82-2.96)	7.26 (7.06-7.46)	1.75 (1.71-1.8)	3.85 (3.64-4.04)	1.33 (1.29-1.36)	3.29 (3.08-3.49)
Eating	Osteoporosis	Persons	4.08 (3.34-4.98)	10.8 (6.13-13.18)	2.48 (2.03-3.03)	4.02 (-0.69-6.34)	1.54 (1.24-1.92)	3.62 (-0.8-6.31)
Personality	Osteoporosis	Persons	2.94 (2.85-3.03)	7.43 (7.15-7.68)	1.78 (1.73-1.84)	3.92 (3.66-4.15)	1.29 (1.25-1.33)	3.14 (2.86-3.4)
Intellectual	Osteoporosis	Persons	4.72 (4.12-5.41)	11.52 (10.08-12.79)	2.87 (2.51-3.29)	5.61 (4.19-6.79)	1.27 (1.11-1.47)	2.49 (1.08-3.71)
Developmental	Osteoporosis	Persons	5.21 (2.8-9.68)	NA	3.17 (1.71-5.9)	NA	2.05 (1.09-3.84)	NA
Behavioral	Osteoporosis	Persons	3.43 (2.85-4.14)	11.34 (8.54-13.42)	2.09 (1.73-2.52)	4.55 (1.73-6.53)	1.23 (1.02-1.5)	4.64 (1.83-6.8)
Organic	Osteoporosis	Males	4.07 (3.89-4.25)	6.18 (5.97-6.42)	1.8 (1.72-1.88)	2.82 (2.63-3.02)	1.28 (1.22-1.33)	0.97 (0.78-1.15)
Substance Use	Osteoporosis	Males	6.14 (5.84-6.45)	11.93 (11.57-12.3)	2.83 (2.69-2.98)	4.71 (4.36-5.08)	1.43 (1.36-1.51)	3.98 (3.64-4.32)
Schizophrenia	Osteoporosis	Males	4.27 (3.9-4.68)	10.71 (9.96-11.43)	1.95 (1.78-2.14)	3.7 (3-4.38)	1.38 (1.26-1.52)	4.22 (3.49-4.92)
Mood	Osteoporosis	Males	3.42 (3.25-3.6)	8.07 (7.69-8.44)	1.56 (1.48-1.64)	2.53 (2.17-2.9)	1.59 (1.51-1.68)	3.89 (3.53-4.25)
Neurotic	Osteoporosis	Males	4.18 (3.95-4.42)	10.33 (9.88-10.77)	1.93 (1.82-2.04)	3.39 (2.96-3.81)	1.7 (1.6-1.8)	5.45 (5.02-5.88)
Eating	Osteoporosis	Males	NA	NA	NA	NA	NA	NA
Personality	Osteoporosis	Males	3.86 (3.6-4.14)	9.68 (9.07-10.24)	1.78 (1.66-1.91)	2.88 (2.32-3.39)	1.52 (1.42-1.63)	4.81 (4.23-5.35)
Intellectual	Osteoporosis	Males	5.01 (4.01-6.26)	12.46 (9.86-14.67)	2.31 (1.85-2.88)	2.98 (0.55-5.05)	1.43 (1.14-1.8)	3.51 (1.04-5.65)
Developmental	Osteoporosis	Males	4.46 (2-9.92)	NA	2.05 (0.92-4.57)	NA	1.87 (0.83-4.21)	NA
Behavioral	Osteoporosis	Males	7.38 (5.3-10.28)	NA	3.4 (2.44-4.74)	NA	2.45 (1.75-3.43)	NA
Organic	Osteoporosis	Females	3.3 (3.23-3.37)	4.78 (4.68-4.89)	2.1 (2.06-2.15)	3.6 (3.5-3.71)	1.15 (1.12-1.17)	0.44 (0.34-0.53)
Substance Use	Osteoporosis	Females	4.05 (3.91-4.19)	9.67 (9.37-9.97)	2.68 (2.59-2.78)	6.92 (6.62-7.2)	1.15 (1.1-1.19)	2.56 (2.26-2.88)

© 2022 Momen NC et al. *JAMA Psychiatry*.

Mental disorder	General medical condition	Sex	Compared to those with neither MD nor GMC		Compared to those with GMC only		Compared to those with MD only	
			MRR	LYLs	MRR	LYLs	MRR	LYLs
Schizophrenia	Osteoporosis	Females	2.88 (2.75-3.01)	7.14 (6.75-7.5)	1.88 (1.8-1.97)	4.69 (4.31-5.03)	1.19 (1.14-1.25)	1.98 (1.6-2.34)
Mood	Osteoporosis	Females	2.4 (2.34-2.45)	5.47 (5.3-5.63)	1.57 (1.54-1.61)	3.32 (3.16-3.48)	1.3 (1.27-1.34)	2.08 (1.9-2.25)
Neurotic	Osteoporosis	Females	2.64 (2.57-2.72)	6.64 (6.42-6.86)	1.74 (1.7-1.79)	3.95 (3.73-4.17)	1.35 (1.31-1.39)	2.84 (2.6-3.09)
Eating	Osteoporosis	Females	3.92 (3.19-4.81)	10.43 (5.88-12.99)	2.59 (2.11-3.18)	3.94 (-0.66-6.52)	1.56 (1.25-1.95)	3.38 (-1.07-6.26)
Personality	Osteoporosis	Females	2.72 (2.63-2.82)	6.95 (6.64-7.24)	1.8 (1.73-1.86)	4.14 (3.83-4.41)	1.32 (1.27-1.37)	2.79 (2.46-3.1)
Intellectual	Osteoporosis	Females	4.52 (3.8-5.38)	10.95 (9.28-12.43)	2.98 (2.51-3.55)	7.23 (5.54-8.7)	1.13 (0.94-1.35)	1.86 (0.13-3.4)
Developmental	Osteoporosis	Females	NA	NA	NA	NA	NA	NA
Behavioral	Osteoporosis	Females	2.71 (2.16-3.4)	8.48 (4.94-10.95)	1.79 (1.43-2.24)	3.86 (0.27-6.29)	1.11 (0.88-1.42)	2.15 (-1.27-4.66)
Organic	Painful conditions	Persons	6.82 (6.75-6.88)	5.96 (5.91-6.01)	2.26 (2.25-2.28)	4.19 (4.14-4.24)	1.8 (1.77-1.83)	0.73 (0.68-0.78)
Substance Use	Painful conditions	Persons	7.79 (7.71-7.88)	13.06 (12.95-13.18)	2.63 (2.6-2.66)	8.27 (8.15-8.39)	1.6 (1.57-1.63)	2.17 (2.03-2.32)
Schizophrenia	Painful conditions	Persons	6.32 (6.22-6.42)	11.24 (11.07-11.4)	2.09 (2.06-2.13)	7.02 (6.85-7.19)	1.94 (1.89-1.99)	3.42 (3.22-3.61)
Mood	Painful conditions	Persons	4.59 (4.55-4.64)	7.74 (7.64-7.84)	1.52 (1.51-1.54)	3.89 (3.78-3.98)	2.04 (2-2.07)	2.36 (2.24-2.47)
Neurotic	Painful conditions	Persons	4.91 (4.86-4.97)	8.92 (8.8-9.04)	1.65 (1.63-1.66)	4.26 (4.14-4.38)	2.16 (2.11-2.21)	2.8 (2.66-2.95)
Eating	Painful conditions	Persons	7.07 (6.38-7.83)	11.26 (9.69-12.5)	2.36 (2.13-2.61)	5.85 (4.27-7.09)	2.93 (2.49-3.46)	3.26 (1.41-5.27)
Personality	Painful conditions	Persons	5.06 (4.99-5.13)	9.51 (9.37-9.66)	1.7 (1.67-1.72)	4.86 (4.72-5.01)	2.15 (2.09-2.21)	2.87 (2.69-3.06)
Intellectual	Painful conditions	Persons	8.11 (7.76-8.47)	13.94 (13.46-14.44)	2.71 (2.59-2.82)	8.88 (8.42-9.38)	1.94 (1.8-2.08)	3.23 (2.64-3.85)
Developmental	Painful conditions	Persons	7.46 (6.37-8.75)	13.1 (10.94-14.81)	2.49 (2.12-2.92)	6.59 (4.41-8.3)	3.08 (2.49-3.79)	4.7 (2.32-7)
Behavioral	Painful conditions	Persons	6.12 (5.77-6.49)	11.76 (11.04-12.52)	2.04 (1.93-2.17)	5.56 (4.85-6.31)	2.37 (2.16-2.61)	3.37 (2.47-4.45)
Organic	Painful conditions	Males	6.91 (6.82-7.01)	6.82 (6.73-6.9)	2.35 (2.32-2.38)	4.5 (4.42-4.58)	1.76 (1.72-1.81)	0.89 (0.8-0.97)
Substance Use	Painful conditions	Males	8.08 (7.96-8.2)	13.64 (13.5-13.79)	2.79 (2.75-2.83)	8.29 (8.14-8.43)	1.62 (1.58-1.66)	2.41 (2.24-2.59)
Schizophrenia	Painful conditions	Males	6.95 (6.78-7.12)	12.95 (12.71-13.21)	2.36 (2.3-2.42)	7.75 (7.52-8.02)	1.92 (1.85-1.99)	4.06 (3.76-4.37)
Mood	Painful conditions	Males	4.75 (4.68-4.83)	8.83 (8.67-8.98)	1.62 (1.59-1.64)	4.26 (4.1-4.41)	1.95 (1.9-2.01)	2.67 (2.48-2.86)
Neurotic	Painful conditions	Males	5.21 (5.12-5.31)	10.19 (10.01-10.37)	1.79 (1.76-1.82)	4.77 (4.58-4.95)	2.01 (1.95-2.07)	3.11 (2.9-3.34)
Eating	Painful conditions	Males	9.01 (6.44-12.61)	NA	3.08 (2.2-4.31)	NA	2.6 (1.6-4.21)	NA
Personality	Painful conditions	Males	5.35 (5.24-5.47)	10.83 (10.6-11.05)	1.84 (1.8-1.88)	5.47 (5.24-5.7)	2.06 (1.98-2.13)	3.35 (3.06-3.64)
Intellectual	Painful conditions	Males	7.55 (7.1-8.02)	13.69 (13.06-14.33)	2.58 (2.43-2.74)	7.96 (7.33-8.57)	2.03 (1.84-2.23)	3.71 (2.88-4.49)
Developmental	Painful conditions	Males	6.87 (5.67-8.32)	12.3 (10.03-14.51)	2.35 (1.94-2.84)	5 (2.69-7.17)	3.11 (2.43-3.98)	5.69 (2.75-8.32)

© 2022 Momen NC et al. *JAMA Psychiatry*.

Mental disorder	General medical condition	Sex	Compared to those with neither MD nor GMC		Compared to those with GMC only		Compared to those with MD only	
			MRR	LYLs	MRR	LYLs	MRR	LYLs
Behavioral	Painful conditions	Males	6.64 (6.14-7.19)	12.97 (11.89-14.04)	2.27 (2.1-2.46)	5.91 (4.82-6.95)	2.45 (2.18-2.76)	4.11 (2.67-5.49)
Organic	Painful conditions	Females	6.85 (6.77-6.93)	5.45 (5.39-5.51)	2.21 (2.19-2.23)	4.01 (3.95-4.06)	1.87 (1.83-1.92)	0.64 (0.58-0.7)
Substance Use	Painful conditions	Females	7.44 (7.31-7.58)	12.28 (12.1-12.47)	2.44 (2.4-2.48)	8.25 (8.06-8.44)	1.67 (1.61-1.74)	1.85 (1.62-2.08)
Schizophrenia	Painful conditions	Females	6.06 (5.94-6.19)	10.1 (9.88-10.29)	1.95 (1.92-1.99)	6.53 (6.32-6.73)	2.13 (2.05-2.21)	2.99 (2.75-3.23)
Mood	Painful conditions	Females	4.58 (4.52-4.64)	7.2 (7.07-7.32)	1.47 (1.46-1.49)	3.7 (3.57-3.82)	2.18 (2.12-2.23)	2.2 (2.05-2.35)
Neurotic	Painful conditions	Females	4.8 (4.73-4.87)	8.23 (8.08-8.39)	1.56 (1.54-1.58)	3.99 (3.83-4.15)	2.43 (2.35-2.51)	2.63 (2.43-2.81)
Eating	Painful conditions	Females	7.07 (6.36-7.87)	11.17 (9.62-12.52)	2.29 (2.06-2.55)	5.82 (4.22-7.16)	3.01 (2.53-3.59)	3.26 (1.39-5.41)
Personality	Painful conditions	Females	4.93 (4.84-5.02)	8.81 (8.63-8.99)	1.6 (1.58-1.63)	4.53 (4.35-4.71)	2.38 (2.29-2.48)	2.62 (2.38-2.86)
Intellectual	Painful conditions	Females	8.81 (8.29-9.37)	14.16 (13.45-14.94)	2.85 (2.68-3.03)	9.72 (9-10.49)	1.73 (1.54-1.93)	2.8 (1.92-3.74)
Developmental	Painful conditions	Females	9.06 (6.81-12.06)	14.13 (9.66-16.81)	2.93 (2.2-3.9)	8.63 (4.16-11.31)	2.64 (1.77-3.94)	3.44 (-1.09-7.66)
Behavioral	Painful conditions	Females	5.61 (5.13-6.13)	10.54 (9.5-11.63)	1.82 (1.66-1.98)	5.21 (4.16-6.31)	2.57 (2.14-3.08)	2.64 (1.25-4.08)
Organic	Hematological system	Persons	5.31 (5.22-5.41)	6.56 (6.45-6.67)	1.27 (1.24-1.29)	1.14 (1.07-1.22)	1.6 (1.57-1.63)	1.82 (1.75-1.9)
Substance Use	Hematological system	Persons	9.29 (9.08-9.51)	15.76 (15.54-15.96)	2.42 (2.36-2.48)	2.04 (1.88-2.21)	2.4 (2.34-2.46)	7.23 (7.03-7.42)
Schizophrenia	Hematological system	Persons	6.3 (6.1-6.51)	13.59 (13.27-13.89)	1.62 (1.57-1.68)	1.54 (1.27-1.79)	2.34 (2.26-2.42)	7.41 (7.13-7.7)
Mood	Hematological system	Persons	4.54 (4.45-4.63)	10.29 (10.1-10.47)	1.14 (1.12-1.17)	0.35 (0.19-0.51)	2.26 (2.21-2.31)	6.4 (6.24-6.57)
Neurotic	Hematological system	Persons	5.65 (5.52-5.78)	13.7 (13.49-13.94)	1.46 (1.42-1.49)	0.54 (0.33-0.74)	2.64 (2.57-2.71)	9.02 (8.8-9.26)
Eating	Hematological system	Persons	10.26 (8.43-12.48)	22.2 (19.62-24.37)	2.66 (2.19-3.24)	1.23 (-1.33-3.42)	4.18 (3.37-5.18)	14.46 (11.95-17.22)
Personality	Hematological system	Persons	6.01 (5.84-6.18)	13.84 (13.56-14.12)	1.55 (1.51-1.6)	0.67 (0.42-0.93)	2.7 (2.61-2.78)	8.85 (8.59-9.14)
Intellectual	Hematological system	Persons	7.4 (6.72-8.16)	16.46 (15.38-17.63)	1.92 (1.74-2.11)	0.23 (-0.81-1.26)	2 (1.8-2.22)	6.92 (5.81-8.03)
Developmental	Hematological system	Persons	8.22 (5.78-11.69)	NA	2.13 (1.5-3.03)	NA	3.26 (2.25-4.71)	NA
Behavioral	Hematological system	Persons	8.92 (7.79-10.23)	22.23 (20.38-24.04)	2.31 (2.02-2.65)	0.26 (-1.53-1.95)	3.3 (2.86-3.82)	14.33 (12.46-16.17)
Organic	Hematological system	Males	6.12 (5.95-6.3)	7.31 (7.13-7.48)	1.3 (1.27-1.34)	1.09 (0.99-1.2)	1.73 (1.68-1.78)	2.05 (1.93-2.17)
Substance Use	Hematological system	Males	11.42 (11.08-11.77)	16.01 (15.77-16.25)	2.62 (2.54-2.7)	1.69 (1.49-1.89)	2.68 (2.6-2.77)	7.3 (7.08-7.51)
Schizophrenia	Hematological system	Males	8.82 (8.38-9.28)	15.14 (14.69-15.6)	2.01 (1.91-2.12)	1.36 (0.99-1.71)	2.82 (2.67-2.97)	8.14 (7.75-8.57)
Mood	Hematological system	Males	5.43 (5.26-5.6)	10.57 (10.29-10.83)	1.22 (1.18-1.26)	0.33 (0.13-0.54)	2.45 (2.36-2.53)	6.33 (6.09-6.58)
Neurotic	Hematological system	Males	7.11 (6.86-7.37)	13.88 (13.57-14.22)	1.63 (1.57-1.69)	0.44 (0.17-0.7)	2.87 (2.76-2.99)	8.69 (8.39-9.01)

© 2022 Momen NC et al. *JAMA Psychiatry*.

Mental disorder	General medical condition	Sex	Compared to those with neither MD nor GMC		Compared to those with GMC only		Compared to those with MD only	
			MRR	LYLs	MRR	LYLs	MRR	LYLs
Eating	Hematological system	Males	14.16 (7.08-28.31)	NA	3.25 (1.63-6.5)	NA	3.89 (1.86-8.15)	NA
Personality	Hematological system	Males	7.55 (7.22-7.88)	13.95 (13.61-14.35)	1.73 (1.65-1.81)	0.57 (0.29-0.87)	2.99 (2.85-3.13)	8.62 (8.26-9)
Intellectual	Hematological system	Males	7.91 (6.88-9.1)	17.12 (15.44-18.77)	1.82 (1.58-2.09)	0.06 (-1.42-1.54)	2.21 (1.9-2.56)	8.32 (6.73-9.89)
Developmental	Hematological system	Males	7.9 (5.04-12.39)	NA	1.81 (1.16-2.84)	NA	3.28 (2.05-5.22)	NA
Behavioral	Hematological system	Males	12.04 (10.02-14.47)	25.26 (22.84-27.53)	2.77 (2.3-3.32)	1.23 (-1.19-3.25)	3.99 (3.29-4.84)	16.91 (14.25-19.28)
Organic	Hematological system	Females	4.83 (4.71-4.94)	6.06 (5.92-6.2)	1.27 (1.24-1.3)	1.18 (1.08-1.29)	1.53 (1.5-1.57)	1.67 (1.57-1.78)
Substance Use	Hematological system	Females	7.33 (7.07-7.59)	15.44 (15.09-15.81)	2.13 (2.05-2.2)	2.49 (2.2-2.77)	2.17 (2.09-2.26)	7.14 (6.83-7.47)
Schizophrenia	Hematological system	Females	5.22 (5-5.44)	12.6 (12.17-13.02)	1.49 (1.43-1.56)	1.65 (1.3-2)	2.19 (2.09-2.29)	6.94 (6.56-7.34)
Mood	Hematological system	Females	4.1 (4-4.2)	10.14 (9.91-10.37)	1.14 (1.12-1.17)	0.36 (0.14-0.57)	2.18 (2.12-2.24)	6.44 (6.22-6.66)
Neurotic	Hematological system	Females	4.92 (4.78-5.07)	13.61 (13.32-13.92)	1.41 (1.37-1.45)	0.59 (0.31-0.87)	2.56 (2.48-2.65)	9.19 (8.9-9.51)
Eating	Hematological system	Females	9.83 (8.01-12.06)	22.11 (19.59-24.4)	2.83 (2.31-3.48)	1.28 (-1.24-3.55)	4.26 (3.4-5.33)	14.47 (11.89-17.51)
Personality	Hematological system	Females	5.17 (4.98-5.37)	13.78 (13.39-14.16)	1.49 (1.43-1.54)	0.73 (0.36-1.07)	2.57 (2.47-2.68)	8.99 (8.59-9.35)
Intellectual	Hematological system	Females	6.96 (6.08-7.97)	15.91 (14.34-17.46)	2 (1.75-2.29)	0.37 (-1.14-1.84)	1.8 (1.56-2.09)	5.73 (4.21-7.27)
Developmental	Hematological system	Females	8.89 (5.05-15.66)	NA	2.56 (1.45-4.51)	NA	2.94 (1.61-5.38)	NA
Behavioral	Hematological system	Females	6.71 (5.47-8.24)	19.88 (17.14-22.36)	1.93 (1.58-2.37)	-0.49 (-3.35-1.92)	2.96 (2.37-3.69)	12.33 (9.55-15.01)
Organic	HIV/AIDS	Persons	14.04 (11.01-17.91)	NA	3.57 (2.78-4.6)	NA	4.93 (3.87-6.29)	NA
Substance Use	HIV/AIDS	Persons	16.43 (14.67-18.4)	NA	5.05 (4.4-5.79)	NA	4.23 (3.78-4.74)	NA
Schizophrenia	HIV/AIDS	Persons	16.2 (13.39-19.59)	NA	4.28 (3.5-5.24)	NA	6.27 (5.18-7.59)	NA
Mood	HIV/AIDS	Persons	6.78 (5.66-8.13)	NA	1.72 (1.42-2.09)	NA	3.54 (2.96-4.25)	NA
Neurotic	HIV/AIDS	Persons	8.7 (7.51-10.07)	NA	2.32 (1.97-2.73)	NA	4.1 (3.54-4.74)	NA
Eating	HIV/AIDS	Persons	NA	NA	NA	NA	NA	NA
Personality	HIV/AIDS	Persons	11.67 (9.89-13.77)	NA	3.12 (2.61-3.73)	NA	5.24 (4.44-6.19)	NA
Intellectual	HIV/AIDS	Persons	10.44 (4.98-21.91)	NA	2.57 (1.22-5.41)	NA	2.89 (1.37-6.06)	NA
Developmental	HIV/AIDS	Persons	NA	NA	NA	NA	NA	NA
Behavioral	HIV/AIDS	Persons	7.69 (4.37-13.54)	NA	1.9 (1.08-3.36)	NA	2.82 (1.6-4.98)	NA

© 2022 Momen NC et al. *JAMA Psychiatry*.

Mental disorder	General medical condition	Sex	Compared to those with neither MD nor GMC		Compared to those with GMC only		Compared to those with MD only	
			MRR	LYLs	MRR	LYLs	MRR	LYLs
Organic	HIV/AIDS	Males	10.68 (7.92-14.41)	NA	2.91 (2.14-3.96)	NA	3.47 (2.58-4.68)	NA
Substance Use	HIV/AIDS	Males	14.26 (12.46-16.32)	NA	4.5 (3.84-5.27)	NA	3.35 (2.93-3.84)	NA
Schizophrenia	HIV/AIDS	Males	14.73 (11.83-18.35)	NA	4.2 (3.33-5.29)	NA	4.85 (3.89-6.04)	NA
Mood	HIV/AIDS	Males	5.93 (4.82-7.3)	NA	1.62 (1.3-2.02)	NA	2.79 (2.27-3.44)	NA
Neurotic	HIV/AIDS	Males	7.69 (6.46-9.16)	NA	2.18 (1.8-2.64)	NA	3.14 (2.63-3.74)	NA
Eating	HIV/AIDS	Males	NA	NA	NA	NA	NA	NA
Personality	HIV/AIDS	Males	10.4 (8.59-12.58)	NA	2.99 (2.44-3.67)	NA	4.13 (3.41-5)	NA
Intellectual	HIV/AIDS	Males	12.88 (5.78-28.66)	NA	3.46 (1.55-7.73)	NA	3.72 (1.67-8.29)	NA
Developmental	HIV/AIDS	Males	NA	NA	NA	NA	NA	NA
Behavioral	HIV/AIDS	Males	7.22 (3.61-14.44)	NA	1.94 (0.97-3.9)	NA	2.39 (1.19-4.79)	NA
Organic	HIV/AIDS	Females	37.4 (24.63-56.81)	NA	6.05 (3.87-9.45)	NA	13.85 (9.12-21.04)	NA
Substance Use	HIV/AIDS	Females	26.87 (21.77-33.15)	NA	6.53 (4.86-8.78)	NA	7.94 (6.43-9.8)	NA
Schizophrenia	HIV/AIDS	Females	23.87 (16.25-35.06)	NA	3.91 (2.58-5.93)	NA	10.39 (7.07-15.26)	NA
Mood	HIV/AIDS	Females	12.89 (8.9-18.67)	NA	2.02 (1.35-3.03)	NA	7.17 (4.95-10.39)	NA
Neurotic	HIV/AIDS	Females	12.92 (9.87-16.92)	NA	2.21 (1.6-3.06)	NA	6.69 (5.11-8.76)	NA
Eating	HIV/AIDS	Females	NA	NA	NA	NA	NA	NA
Personality	HIV/AIDS	Females	19.26 (13.76-26.96)	NA	3.23 (2.22-4.69)	NA	9.49 (6.78-13.28)	NA
Intellectual	HIV/AIDS	Females	NA	NA	NA	NA	NA	NA
Developmental	HIV/AIDS	Females	NA	NA	NA	NA	NA	NA
Behavioral	HIV/AIDS	Females	NA	NA	NA	NA	NA	NA
Organic	Anemias	Persons	5.3 (5.2-5.4)	6.47 (6.37-6.57)	1.26 (1.24-1.29)	1.1 (1.03-1.18)	1.6 (1.57-1.63)	1.8 (1.73-1.88)
Substance Use	Anemias	Persons	9.16 (8.95-9.38)	15.35 (15.14-15.55)	2.38 (2.33-2.44)	1.77 (1.61-1.95)	2.36 (2.3-2.42)	7.1 (6.91-7.28)
Schizophrenia	Anemias	Persons	6.21 (6.01-6.41)	13.18 (12.9-13.47)	1.6 (1.55-1.65)	1.36 (1.11-1.61)	2.3 (2.22-2.38)	7.21 (6.92-7.49)
Mood	Anemias	Persons	4.53 (4.44-4.62)	10.1 (9.91-10.28)	1.14 (1.12-1.16)	0.31 (0.14-0.47)	2.25 (2.2-2.3)	6.31 (6.14-6.49)
Neurotic	Anemias	Persons	5.62 (5.49-5.75)	13.5 (13.25-13.75)	1.45 (1.41-1.48)	0.5 (0.27-0.72)	2.62 (2.55-2.68)	8.94 (8.69-9.19)
Eating	Anemias	Persons	10.21 (8.37-12.44)	NA	2.65 (2.17-3.23)	NA	4.15 (3.34-5.15)	NA

© 2022 Momen NC et al. *JAMA Psychiatry*.

Mental disorder	General medical condition	Sex	Compared to those with neither MD nor GMC		Compared to those with GMC only		Compared to those with MD only	
			MRR	LYLs	MRR	LYLs	MRR	LYLs
Personality	Anemias	Persons	5.94 (5.77-6.11)	13.56 (13.29-13.87)	1.53 (1.49-1.58)	0.55 (0.3-0.83)	2.66 (2.58-2.74)	8.72 (8.45-9.01)
Intellectual	Anemias	Persons	7.34 (6.65-8.09)	16.27 (15.17-17.33)	1.9 (1.72-2.1)	-0.22 (-1.3-0.8)	1.98 (1.79-2.2)	6.77 (5.69-7.83)
Developmental	Anemias	Persons	8.14 (5.69-11.64)	NA	2.11 (1.48-3.02)	NA	3.22 (2.22-4.69)	NA
Behavioral	Anemias	Persons	9.04 (7.86-10.4)	22.68 (20.69-24.4)	2.34 (2.04-2.7)	0.17 (-1.7-2)	3.34 (2.88-3.87)	14.84 (12.79-16.63)
Organic	Anemias	Males	6.1 (5.93-6.28)	7.17 (7-7.33)	1.3 (1.26-1.34)	1.01 (0.91-1.12)	1.72 (1.67-1.77)	2.03 (1.91-2.14)
Substance Use	Anemias	Males	11.31 (10.97-11.67)	15.53 (15.28-15.78)	2.59 (2.51-2.67)	1.29 (1.1-1.48)	2.65 (2.56-2.74)	7.19 (6.97-7.41)
Schizophrenia	Anemias	Males	8.63 (8.2-9.09)	14.44 (14.01-14.89)	1.97 (1.87-2.07)	0.99 (0.67-1.31)	2.75 (2.6-2.9)	7.84 (7.46-8.24)
Mood	Anemias	Males	5.41 (5.24-5.59)	10.15 (9.89-10.41)	1.21 (1.17-1.25)	0.23 (0.01-0.42)	2.44 (2.35-2.52)	6.13 (5.91-6.37)
Neurotic	Anemias	Males	7.09 (6.83-7.35)	13.44 (13.14-13.77)	1.62 (1.56-1.68)	0.33 (0.08-0.57)	2.86 (2.74-2.97)	8.55 (8.27-8.86)
Eating	Anemias	Males	13.03 (6.21-27.33)	NA	2.99 (1.42-6.27)	NA	3.54 (1.62-7.74)	NA
Personality	Anemias	Males	7.45 (7.13-7.79)	13.42 (13.03-13.81)	1.7 (1.63-1.78)	0.36 (0.08-0.67)	2.94 (2.8-3.08)	8.43 (8.07-8.78)
Intellectual	Anemias	Males	7.76 (6.74-8.95)	16.71 (15.14-18.36)	1.78 (1.54-2.05)	-0.93 (-2.43-0.54)	2.16 (1.86-2.52)	8.04 (6.53-9.57)
Developmental	Anemias	Males	8.11 (5.17-12.71)	NA	1.86 (1.19-2.92)	NA	3.37 (2.11-5.38)	NA
Behavioral	Anemias	Males	12.71 (10.52-15.35)	26.83 (24.4-28.94)	2.91 (2.41-3.52)	1.23 (-1.11-3.35)	4.21 (3.45-5.13)	18.57 (16.11-20.97)
Organic	Anemias	Females	4.82 (4.71-4.93)	6.02 (5.89-6.15)	1.27 (1.24-1.3)	1.16 (1.07-1.26)	1.53 (1.49-1.57)	1.65 (1.55-1.76)
Substance Use	Anemias	Females	7.22 (6.97-7.49)	15.13 (14.81-15.44)	2.09 (2.02-2.17)	2.36 (2.08-2.62)	2.13 (2.05-2.22)	6.98 (6.69-7.31)
Schizophrenia	Anemias	Females	5.18 (4.97-5.4)	12.43 (12.01-12.87)	1.48 (1.42-1.55)	1.59 (1.24-1.93)	2.17 (2.08-2.27)	6.83 (6.45-7.25)
Mood	Anemias	Females	4.09 (3.99-4.19)	10.08 (9.83-10.32)	1.14 (1.11-1.17)	0.34 (0.13-0.57)	2.18 (2.12-2.23)	6.4 (6.17-6.64)
Neurotic	Anemias	Females	4.9 (4.76-5.05)	13.52 (13.19-13.82)	1.4 (1.36-1.45)	0.57 (0.28-0.89)	2.55 (2.47-2.63)	9.13 (8.81-9.45)
Eating	Anemias	Females	9.85 (8.02-12.1)	NA	2.84 (2.31-3.49)	NA	4.27 (3.41-5.35)	NA
Personality	Anemias	Females	5.14 (4.95-5.33)	13.63 (13.22-14.05)	1.48 (1.42-1.53)	0.65 (0.28-1.02)	2.55 (2.45-2.66)	8.87 (8.49-9.27)
Intellectual	Anemias	Females	6.97 (6.09-7.98)	15.91 (14.29-17.43)	2.01 (1.75-2.3)	0.36 (-1.15-1.79)	1.81 (1.56-2.09)	5.73 (4.18-7.26)
Developmental	Anemias	Females	8.29 (4.59-14.98)	NA	2.39 (1.32-4.32)	NA	2.72 (1.45-5.09)	NA
Behavioral	Anemias	Females	6.62 (5.37-8.16)	19.79 (16.94-22.41)	1.91 (1.55-2.35)	-0.58 (-3.4-2.1)	2.9 (2.32-3.64)	12.24 (9.3-15.13)
Organic	Cancers	Persons	7.51 (7.38-7.65)	7.72 (7.61-7.83)	1.34 (1.32-1.37)	2.35 (2.27-2.43)	1.87 (1.84-1.91)	2.39 (2.3-2.47)
Substance Use	Cancers	Persons	15.17 (14.89-15.46)	15.71 (15.56-15.85)	2.95 (2.89-3)	3.89 (3.77-4.01)	3.51 (3.44-3.59)	7.76 (7.61-7.9)

© 2022 Momen NC et al. *JAMA Psychiatry*.

Mental disorder	General medical condition	Sex	Compared to those with neither MD nor GMC		Compared to those with GMC only		Compared to those with MD only	
			MRR	LYLs	MRR	LYLs	MRR	LYLs
Schizophrenia	Cancers	Persons	10.06 (9.79-10.33)	14.5 (14.27-14.74)	1.96 (1.91-2.01)	3.28 (3.1-3.48)	3.34 (3.24-3.44)	8.26 (8.03-8.49)
Mood	Cancers	Persons	6.76 (6.66-6.87)	11.33 (11.18-11.48)	1.29 (1.27-1.31)	1.65 (1.52-1.77)	2.99 (2.94-3.04)	7.17 (7.02-7.3)
Neurotic	Cancers	Persons	8.83 (8.67-8.98)	14 (13.84-14.16)	1.72 (1.69-1.75)	2 (1.86-2.15)	3.84 (3.77-3.92)	9.35 (9.17-9.51)
Eating	Cancers	Persons	16.68 (14.07-19.77)	NA	3.29 (2.78-3.9)	NA	6.6 (5.44-8)	NA
Personality	Cancers	Persons	9.68 (9.47-9.89)	14.12 (13.92-14.31)	1.9 (1.86-1.94)	2.51 (2.32-2.69)	4.04 (3.94-4.14)	9.36 (9.15-9.57)
Intellectual	Cancers	Persons	14.5 (13.27-15.84)	18.94 (17.92-19.95)	2.86 (2.61-3.12)	4.57 (3.8-5.29)	3.39 (3.08-3.73)	9.85 (8.93-10.75)
Developmental	Cancers	Persons	18.55 (14.14-24.35)	NA	3.66 (2.79-4.81)	NA	7.01 (5.22-9.4)	NA
Behavioral	Cancers	Persons	11.69 (10.5-13.02)	23.53 (22.25-24.94)	2.31 (2.07-2.57)	3.27 (2.06-4.6)	4.06 (3.6-4.58)	16.13 (14.7-17.6)
Organic	Cancers	Males	8.53 (8.31-8.75)	7.91 (7.76-8.08)	1.46 (1.42-1.49)	2.28 (2.18-2.38)	1.93 (1.88-1.99)	2.41 (2.29-2.52)
Substance Use	Cancers	Males	17.56 (17.14-17.99)	15.63 (15.45-15.82)	3.2 (3.12-3.28)	3.48 (3.33-3.62)	3.62 (3.52-3.72)	7.68 (7.49-7.85)
Schizophrenia	Cancers	Males	13.27 (12.73-13.83)	15.42 (15.05-15.78)	2.45 (2.35-2.55)	3.26 (2.99-3.52)	3.68 (3.51-3.85)	8.75 (8.43-9.1)
Mood	Cancers	Males	7.92 (7.72-8.13)	11.11 (10.9-11.33)	1.44 (1.4-1.47)	1.53 (1.36-1.7)	3.09 (3-3.18)	6.74 (6.54-6.95)
Neurotic	Cancers	Males	10.29 (10-10.58)	13.87 (13.61-14.12)	1.9 (1.84-1.95)	1.78 (1.58-1.98)	3.69 (3.58-3.82)	8.83 (8.57-9.06)
Eating	Cancers	Males	42.43 (25.99-69.26)	NA	7.94 (4.86-12.96)	NA	11.99 (6.83-21.06)	NA
Personality	Cancers	Males	11.08 (10.71-11.46)	13.69 (13.43-13.96)	2.05 (1.98-2.12)	2.24 (2.01-2.45)	3.92 (3.77-4.08)	8.78 (8.51-9.06)
Intellectual	Cancers	Males	17.46 (15.34-19.88)	19.77 (18.37-21.14)	3.26 (2.86-3.71)	4.23 (3.16-5.26)	4.16 (3.62-4.78)	11.16 (9.9-12.43)
Developmental	Cancers	Males	20.95 (14.73-29.79)	NA	3.92 (2.75-5.57)	NA	8.14 (5.6-11.84)	NA
Behavioral	Cancers	Males	16.48 (14.18-19.14)	25.84 (24.16-27.49)	3.08 (2.65-3.58)	3.54 (1.94-5.13)	5.07 (4.31-5.97)	17.91 (16.02-19.75)
Organic	Cancers	Females	6.8 (6.64-6.96)	7.56 (7.41-7.71)	1.27 (1.24-1.3)	2.41 (2.3-2.52)	1.81 (1.76-1.86)	2.37 (2.26-2.49)
Substance Use	Cancers	Females	12.7 (12.34-13.08)	15.81 (15.58-16.06)	2.62 (2.55-2.7)	4.44 (4.24-4.66)	3.48 (3.37-3.6)	7.88 (7.64-8.14)
Schizophrenia	Cancers	Females	8.53 (8.24-8.83)	13.93 (13.64-14.23)	1.75 (1.69-1.81)	3.3 (3.04-3.56)	3.26 (3.13-3.39)	7.96 (7.65-8.26)
Mood	Cancers	Females	6.14 (6.01-6.26)	11.46 (11.25-11.64)	1.23 (1.2-1.25)	1.72 (1.54-1.88)	2.94 (2.87-3.01)	7.41 (7.2-7.6)
Neurotic	Cancers	Females	8.03 (7.85-8.21)	14.07 (13.86-14.29)	1.66 (1.62-1.69)	2.11 (1.91-2.32)	4.03 (3.92-4.14)	9.62 (9.4-9.86)
Eating	Cancers	Females	15.11 (12.61-18.11)	NA	3.14 (2.62-3.77)	NA	6.31 (5.14-7.74)	NA
Personality	Cancers	Females	8.81 (8.57-9.07)	14.37 (14.08-14.64)	1.83 (1.77-1.88)	2.68 (2.4-2.91)	4.2 (4.06-4.35)	9.71 (9.41-9.99)
Intellectual	Cancers	Females	12.56 (11.14-14.18)	18.3 (16.99-19.63)	2.61 (2.31-2.94)	4.83 (3.7-5.85)	2.85 (2.5-3.26)	8.84 (7.59-10.07)

© 2022 Momen NC et al. *JAMA Psychiatry*.

Mental disorder	General medical condition	Sex	Compared to those with neither MD nor GMC		Compared to those with GMC only		Compared to those with MD only	
			MRR	LYLs	MRR	LYLs	MRR	LYLs
Developmental	Cancers	Females	15.89 (10.36-24.37)	NA	3.31 (2.15-5.07)	NA	5.26 (3.24-8.53)	NA
Behavioral	Cancers	Females	8.86 (7.59-10.34)	21.04 (19.04-23.12)	1.84 (1.58-2.15)	2.98 (0.88-4.88)	3.76 (3.14-4.5)	14.21 (12.12-16.47)
Organic	Neurological system	Persons	3.04 (3.01-3.07)	5.27 (5.21-5.34)	2.5 (2.47-2.52)	5.02 (4.95-5.08)	0.93 (0.91-0.94)	0.12 (0.06-0.18)
Substance Use	Neurological system	Persons	4.05 (3.99-4.12)	11.98 (11.83-12.16)	3.42 (3.37-3.48)	10.17 (10.02-10.36)	0.98 (0.96-1)	1.85 (1.69-2.04)
Schizophrenia	Neurological system	Persons	2.72 (2.67-2.78)	9.48 (9.24-9.7)	2.28 (2.23-2.33)	7.91 (7.68-8.13)	0.97 (0.95-1)	1.91 (1.66-2.16)
Mood	Neurological system	Persons	2.05 (2.03-2.08)	6.18 (6.05-6.3)	1.72 (1.7-1.74)	4.97 (4.84-5.1)	0.99 (0.97-1)	1.13 (0.98-1.27)
Neurotic	Neurological system	Persons	2.34 (2.3-2.37)	7.52 (7.37-7.68)	1.97 (1.95-2)	5.82 (5.65-5.97)	1.05 (1.03-1.07)	1.69 (1.51-1.87)
Eating	Neurological system	Persons	3.76 (3.28-4.32)	9.82 (7.14-11.6)	3.17 (2.76-3.64)	7.74 (5.04-9.53)	1.56 (1.31-1.84)	1.79 (-1.09-4.14)
Personality	Neurological system	Persons	2.42 (2.37-2.46)	7.93 (7.73-8.12)	2.04 (2-2.08)	6.32 (6.11-6.52)	1.03 (1.01-1.06)	1.74 (1.52-1.96)
Intellectual	Neurological system	Persons	4.69 (4.45-4.95)	14.62 (13.96-15.28)	3.96 (3.76-4.18)	11.74 (11.06-12.38)	1.41 (1.31-1.51)	3.23 (2.46-4.01)
Developmental	Neurological system	Persons	4.51 (3.74-5.44)	12.35 (8.83-14.42)	3.8 (3.15-4.59)	8.22 (4.72-10.29)	2.03 (1.62-2.55)	4.27 (0.76-6.87)
Behavioral	Neurological system	Persons	3.23 (2.98-3.5)	11.06 (9.97-12.14)	2.72 (2.51-2.95)	7.61 (6.54-8.7)	1.2 (1.09-1.33)	2.62 (1.3-3.86)
Organic	Neurological system	Males	3.32 (3.27-3.37)	5.98 (5.89-6.08)	2.66 (2.62-2.71)	5.46 (5.37-5.55)	0.94 (0.92-0.96)	0.25 (0.15-0.34)
Substance Use	Neurological system	Males	4.62 (4.52-4.72)	12.65 (12.45-12.87)	3.76 (3.69-3.84)	10.27 (10.06-10.5)	1.03 (1-1.05)	2.17 (1.95-2.39)
Schizophrenia	Neurological system	Males	3.34 (3.22-3.45)	11.23 (10.87-11.6)	2.71 (2.62-2.8)	8.78 (8.43-9.13)	1.03 (0.99-1.07)	2.63 (2.26-3)
Mood	Neurological system	Males	2.27 (2.23-2.32)	7.09 (6.89-7.3)	1.85 (1.81-1.88)	5.29 (5.09-5.5)	0.98 (0.95-1)	1.45 (1.24-1.68)
Neurotic	Neurological system	Males	2.75 (2.69-2.82)	9.01 (8.75-9.25)	2.25 (2.2-2.31)	6.47 (6.22-6.73)	1.08 (1.05-1.11)	2.31 (2.05-2.58)
Eating	Neurological system	Males	5.94 (3.83-9.21)	NA	4.86 (3.13-7.53)	NA	1.77 (1.05-2.99)	NA
Personality	Neurological system	Males	2.8 (2.72-2.88)	9.03 (8.73-9.34)	2.28 (2.22-2.35)	6.7 (6.39-6.99)	1.06 (1.02-1.1)	2.21 (1.87-2.52)
Intellectual	Neurological system	Males	4.58 (4.25-4.93)	14.18 (13.25-15.04)	3.75 (3.48-4.04)	10.35 (9.41-11.22)	1.42 (1.29-1.56)	3.3 (2.24-4.36)
Developmental	Neurological system	Males	4.49 (3.59-5.61)	11.74 (7.55-14.3)	3.66 (2.93-4.58)	6.79 (2.6-9.36)	2.15 (1.65-2.81)	5.06 (0.85-8.13)
Behavioral	Neurological system	Males	4.06 (3.64-4.53)	12.92 (11.31-14.35)	3.32 (2.98-3.7)	8.35 (6.73-9.79)	1.4 (1.23-1.59)	3.98 (2.09-5.59)
Organic	Neurological system	Females	2.86 (2.82-2.89)	4.79 (4.72-4.87)	2.4 (2.37-2.44)	4.72 (4.64-4.79)	0.91 (0.9-0.93)	0.04 (-0.03-0.11)
Substance Use	Neurological system	Females	3.49 (3.4-3.57)	11.16 (10.92-11.42)	3.05 (2.98-3.12)	10.05 (9.82-10.31)	0.96 (0.93-0.99)	1.47 (1.19-1.75)
Schizophrenia	Neurological system	Females	2.42 (2.36-2.49)	8.35 (8.01-8.64)	2.09 (2.04-2.15)	7.36 (7.02-7.64)	0.98 (0.95-1.01)	1.45 (1.1-1.76)
Mood	Neurological system	Females	1.93 (1.9-1.96)	5.75 (5.59-5.9)	1.67 (1.64-1.7)	4.82 (4.65-4.98)	1.01 (0.99-1.03)	0.98 (0.79-1.16)

© 2022 Momen NC et al. *JAMA Psychiatry*.

Mental disorder	General medical condition	Sex	Compared to those with neither MD nor GMC		Compared to those with GMC only		Compared to those with MD only	
			MRR	LYLs	MRR	LYLs	MRR	LYLs
Neurotic	Neurological system	Females	2.12 (2.08-2.16)	6.8 (6.61-7)	1.84 (1.81-1.88)	5.5 (5.29-5.7)	1.07 (1.05-1.1)	1.38 (1.15-1.61)
Eating	Neurological system	Females	3.57 (3.08-4.12)	9.57 (6.87-11.41)	3.09 (2.68-3.58)	7.57 (4.83-9.42)	1.55 (1.3-1.85)	1.62 (-1.27-4.03)
Personality	Neurological system	Females	2.21 (2.15-2.26)	7.41 (7.14-7.66)	1.92 (1.88-1.97)	6.14 (5.87-6.4)	1.06 (1.03-1.09)	1.52 (1.22-1.82)
Intellectual	Neurological system	Females	4.81 (4.46-5.19)	15.15 (14.16-16.11)	4.18 (3.88-4.51)	13.36 (12.37-14.31)	1.39 (1.25-1.54)	3.14 (2.06-4.29)
Developmental	Neurological system	Females	4.64 (3.28-6.56)	13.56 (8.42-16.96)	4.02 (2.84-5.69)	11.04 (5.95-14.52)	1.64 (1.07-2.51)	2.69 (-2.45-7.39)
Behavioral	Neurological system	Females	2.58 (2.3-2.91)	8.89 (7.25-10.46)	2.24 (1.99-2.53)	6.76 (5.15-8.31)	1.12 (0.96-1.31)	1.04 (-0.65-2.82)
Organic	Vision problems	Persons	2.65 (2.61-2.69)	3.82 (3.76-3.89)	2.32 (2.29-2.35)	3.78 (3.72-3.85)	0.86 (0.84-0.87)	-0.1 (-0.16--0.04)
Substance Use	Vision problems	Persons	2.96 (2.88-3.04)	7.65 (7.42-7.85)	2.67 (2.6-2.74)	6.1 (5.87-6.3)	0.71 (0.69-0.73)	1.06 (0.83-1.26)
Schizophrenia	Vision problems	Persons	2.13 (2.07-2.21)	5.43 (5.16-5.7)	1.93 (1.86-1.99)	4.32 (4.06-4.57)	0.78 (0.75-0.8)	0.67 (0.41-0.93)
Mood	Vision problems	Persons	1.76 (1.73-1.79)	3.64 (3.51-3.76)	1.58 (1.56-1.61)	2.97 (2.84-3.1)	0.87 (0.85-0.89)	0.57 (0.43-0.7)
Neurotic	Vision problems	Persons	1.91 (1.87-1.96)	4.69 (4.5-4.87)	1.73 (1.69-1.77)	3.43 (3.25-3.62)	0.86 (0.84-0.88)	1.11 (0.91-1.3)
Eating	Vision problems	Persons	3.35 (2.54-4.4)	NA	3.05 (2.32-4.02)	NA	1.27 (0.96-1.7)	NA
Personality	Vision problems	Persons	1.95 (1.89-2)	4.91 (4.68-5.13)	1.77 (1.72-1.82)	3.66 (3.44-3.88)	0.83 (0.81-0.86)	1.13 (0.89-1.37)
Intellectual	Vision problems	Persons	3.67 (3.34-4.03)	11.74 (10.57-12.83)	3.35 (3.05-3.67)	7.8 (6.65-8.86)	1 (0.91-1.11)	2.54 (1.4-3.62)
Developmental	Vision problems	Persons	4.53 (3.13-6.56)	NA	4.13 (2.85-5.99)	NA	1.83 (1.25-2.7)	NA
Behavioral	Vision problems	Persons	2.31 (1.97-2.71)	11.21 (8.46-13.5)	2.1 (1.79-2.47)	5.85 (3.18-8.04)	0.82 (0.69-0.97)	4.44 (1.77-6.74)
Organic	Vision problems	Males	2.83 (2.76-2.89)	4.24 (4.12-4.36)	2.4 (2.35-2.46)	3.91 (3.8-4.02)	0.85 (0.83-0.87)	0.02 (-0.09-0.11)
Substance Use	Vision problems	Males	3.37 (3.24-3.5)	8.31 (8.01-8.64)	2.92 (2.81-3.04)	6.2 (5.91-6.51)	0.75 (0.72-0.78)	1.3 (1.01-1.62)
Schizophrenia	Vision problems	Males	2.46 (2.31-2.61)	6.6 (6.1-7.07)	2.15 (2.02-2.29)	4.65 (4.19-5.08)	0.77 (0.72-0.82)	1.06 (0.61-1.5)
Mood	Vision problems	Males	1.91 (1.85-1.97)	4.34 (4.11-4.57)	1.67 (1.61-1.72)	3.14 (2.92-3.37)	0.85 (0.82-0.88)	0.83 (0.6-1.05)
Neurotic	Vision problems	Males	2.2 (2.11-2.29)	5.78 (5.46-6.11)	1.93 (1.86-2.01)	3.84 (3.54-4.16)	0.86 (0.83-0.9)	1.58 (1.26-1.92)
Eating	Vision problems	Males	5.25 (2.19-12.62)	NA	4.65 (1.93-11.17)	NA	1.44 (0.58-3.58)	NA
Personality	Vision problems	Males	2.24 (2.12-2.35)	5.72 (5.33-6.12)	1.97 (1.87-2.07)	3.85 (3.46-4.23)	0.85 (0.81-0.9)	1.51 (1.09-1.91)
Intellectual	Vision problems	Males	3.49 (3.03-4.03)	10.91 (9.27-12.55)	3.09 (2.67-3.56)	5.95 (4.34-7.55)	0.99 (0.85-1.15)	1.9 (0.21-3.54)
Developmental	Vision problems	Males	3.82 (2.26-6.45)	NA	3.38 (2-5.71)	NA	1.63 (0.95-2.79)	NA
Behavioral	Vision problems	Males	2.45 (1.9-3.17)	12.21 (8.19-15.57)	2.17 (1.68-2.8)	5.62 (1.48-8.93)	0.79 (0.61-1.03)	4.75 (0.7-8.23)
Organic	Vision problems	Females	2.55 (2.51-2.6)	3.61 (3.54-3.69)	2.29 (2.25-2.33)	3.72 (3.64-3.8)	0.87 (0.85-0.89)	-0.15 (-0.23--0.08)

© 2022 Momen NC et al. *JAMA Psychiatry*.

Mental disorder	General medical condition	Sex	Compared to those with neither MD nor GMC		Compared to those with GMC only		Compared to those with MD only	
			MRR	LYLs	MRR	LYLs	MRR	LYLs
Substance Use	Vision problems	Females	2.64 (2.54-2.74)	7.04 (6.72-7.31)	2.46 (2.36-2.55)	6.02 (5.71-6.29)	0.72 (0.69-0.75)	0.84 (0.52-1.11)
Schizophrenia	Vision problems	Females	2.01 (1.94-2.09)	4.93 (4.64-5.25)	1.86 (1.79-1.94)	4.17 (3.89-4.48)	0.83 (0.79-0.86)	0.51 (0.21-0.82)
Mood	Vision problems	Females	1.7 (1.67-1.73)	3.36 (3.21-3.5)	1.56 (1.53-1.6)	2.9 (2.76-3.05)	0.9 (0.88-0.92)	0.46 (0.3-0.61)
Neurotic	Vision problems	Females	1.8 (1.75-1.84)	4.22 (4.01-4.47)	1.66 (1.62-1.71)	3.26 (3.04-3.48)	0.89 (0.86-0.92)	0.91 (0.68-1.15)
Eating	Vision problems	Females	3.2 (2.4-4.27)	NA	2.98 (2.23-3.98)	NA	1.27 (0.94-1.72)	NA
Personality	Vision problems	Females	1.83 (1.76-1.89)	4.56 (4.28-4.84)	1.7 (1.64-1.76)	3.58 (3.29-3.86)	0.86 (0.83-0.89)	0.97 (0.67-1.27)
Intellectual	Vision problems	Females	3.81 (3.37-4.3)	12.49 (10.96-13.99)	3.55 (3.14-4.01)	9.47 (8.06-10.85)	0.98 (0.86-1.13)	3.12 (1.61-4.64)
Developmental	Vision problems	Females	5.57 (3.3-9.41)	NA	5.2 (3.08-8.78)	NA	1.87 (1.06-3.3)	NA
Behavioral	Vision problems	Females	2.22 (1.81-2.72)	10.26 (6.6-13.59)	2.07 (1.68-2.53)	6.08 (2.45-9.29)	0.93 (0.75-1.16)	4.14 (0.49-7.54)
Organic	Hearing problems	Persons	2.38 (2.34-2.41)	3.72 (3.65-3.79)	2.49 (2.46-2.53)	3.98 (3.91-4.06)	0.8 (0.79-0.81)	-0.26 (-0.32--0.19)
Substance Use	Hearing problems	Persons	2.52 (2.44-2.6)	8.08 (7.77-8.35)	2.67 (2.58-2.75)	8.23 (7.93-8.52)	0.62 (0.6-0.64)	-0.29 (-0.59--0.01)
Schizophrenia	Hearing problems	Persons	1.87 (1.81-1.94)	5.73 (5.35-6.07)	1.99 (1.92-2.06)	5.78 (5.41-6.13)	0.69 (0.67-0.72)	-0.32 (-0.71-0.02)
Mood	Hearing problems	Persons	1.52 (1.49-1.55)	3.44 (3.28-3.62)	1.61 (1.58-1.64)	3.58 (3.42-3.76)	0.76 (0.75-0.78)	-0.47 (-0.63--0.28)
Neurotic	Hearing problems	Persons	1.62 (1.58-1.66)	4.55 (4.29-4.81)	1.73 (1.68-1.77)	4.5 (4.24-4.77)	0.73 (0.71-0.75)	-0.39 (-0.66--0.13)
Eating	Hearing problems	Persons	2.17 (1.49-3.17)	NA	2.33 (1.6-3.4)	NA	0.81 (0.55-1.2)	NA
Personality	Hearing problems	Persons	1.59 (1.54-1.65)	4.47 (4.14-4.78)	1.7 (1.64-1.76)	4.48 (4.14-4.79)	0.68 (0.66-0.71)	-0.68 (-1.01--0.34)
Intellectual	Hearing problems	Persons	3.69 (3.34-4.07)	13.47 (12.18-14.63)	3.96 (3.59-4.37)	12.87 (11.55-14.01)	1.03 (0.93-1.15)	2.35 (1.06-3.61)
Developmental	Hearing problems	Persons	2.48 (1.56-3.94)	NA	2.66 (1.68-4.23)	NA	0.98 (0.61-1.57)	NA
Behavioral	Hearing problems	Persons	2.01 (1.72-2.36)	8.25 (5.94-10.37)	2.16 (1.85-2.53)	7.36 (4.96-9.54)	0.72 (0.61-0.85)	-0.06 (-2.49-2.28)
Organic	Hearing problems	Males	2.52 (2.46-2.57)	4.13 (4.03-4.24)	2.74 (2.68-2.8)	4.43 (4.32-4.55)	0.78 (0.76-0.8)	-0.29 (-0.38--0.19)
Substance Use	Hearing problems	Males	2.73 (2.63-2.85)	8.42 (8.02-8.79)	2.95 (2.83-3.07)	8.71 (8.31-9.08)	0.62 (0.59-0.64)	-0.44 (-0.86--0.07)
Schizophrenia	Hearing problems	Males	2.09 (1.96-2.22)	6.78 (6.13-7.4)	2.28 (2.15-2.42)	6.97 (6.31-7.58)	0.66 (0.62-0.71)	-0.53 (-1.17-0.08)
Mood	Hearing problems	Males	1.58 (1.53-1.63)	3.74 (3.47-4.01)	1.72 (1.67-1.78)	4.05 (3.78-4.32)	0.71 (0.68-0.73)	-0.8 (-1.08--0.51)
Neurotic	Hearing problems	Males	1.75 (1.69-1.83)	5.01 (4.6-5.43)	1.92 (1.85-2)	5.22 (4.8-5.62)	0.68 (0.66-0.71)	-0.69 (-1.1--0.3)
Eating	Hearing problems	Males	2.5 (1.04-6.01)	NA	2.76 (1.15-6.64)	NA	0.66 (0.26-1.64)	NA
Personality	Hearing problems	Males	1.65 (1.57-1.74)	4.59 (4.07-5.04)	1.81 (1.72-1.91)	4.87 (4.34-5.35)	0.62 (0.59-0.66)	-1.15 (-1.68--0.66)
Intellectual	Hearing problems	Males	3.21 (2.79-3.69)	12.89 (11.18-14.64)	3.54 (3.08-4.07)	12.53 (10.85-14.25)	0.93 (0.8-1.08)	2.19 (0.39-3.99)

© 2022 Momen NC et al. *JAMA Psychiatry*.

Mental disorder	General medical condition	Sex	Compared to those with neither MD nor GMC		Compared to those with GMC only		Compared to those with MD only	
			MRR	LYLs	MRR	LYLs	MRR	LYLs
Developmental	Hearing problems	Males	2.53 (1.5-4.28)	NA	2.8 (1.66-4.73)	NA	1.08 (0.63-1.85)	NA
Behavioral	Hearing problems	Males	2.21 (1.77-2.75)	8.87 (5.73-11.67)	2.44 (1.95-3.04)	8.3 (5.23-11.16)	0.72 (0.57-0.9)	0.1 (-3.17-3.18)
Organic	Hearing problems	Females	2.28 (2.24-2.33)	3.41 (3.31-3.5)	2.31 (2.27-2.36)	3.65 (3.55-3.74)	0.81 (0.79-0.82)	-0.23 (-0.32--0.15)
Substance Use	Hearing problems	Females	2.28 (2.18-2.39)	7.59 (7.16-8.07)	2.37 (2.26-2.48)	7.57 (7.11-8.03)	0.64 (0.61-0.67)	-0.06 (-0.51-0.38)
Schizophrenia	Hearing problems	Females	1.78 (1.71-1.86)	5.05 (4.62-5.49)	1.85 (1.77-1.93)	5.02 (4.59-5.48)	0.74 (0.71-0.78)	-0.19 (-0.61-0.25)
Mood	Hearing problems	Females	1.49 (1.46-1.53)	3.27 (3.03-3.48)	1.54 (1.5-1.58)	3.3 (3.07-3.52)	0.8 (0.78-0.82)	-0.26 (-0.49--0.03)
Neurotic	Hearing problems	Females	1.54 (1.49-1.59)	4.24 (3.9-4.6)	1.6 (1.55-1.65)	4.03 (3.68-4.39)	0.77 (0.74-0.79)	-0.2 (-0.55-0.17)
Eating	Hearing problems	Females	2.12 (1.4-3.22)	NA	2.21 (1.46-3.36)	NA	0.82 (0.53-1.25)	NA
Personality	Hearing problems	Females	1.56 (1.49-1.62)	4.4 (3.96-4.84)	1.62 (1.55-1.69)	4.25 (3.8-4.68)	0.73 (0.7-0.77)	-0.38 (-0.83-0.08)
Intellectual	Hearing problems	Females	4.34 (3.77-4.99)	14.16 (12.21-15.87)	4.53 (3.93-5.21)	13.29 (11.32-14.96)	1.16 (1-1.35)	2.54 (0.6-4.34)
Developmental	Hearing problems	Females	NA	NA	NA	NA	NA	NA
Behavioral	Hearing problems	Females	1.85 (1.47-2.32)	7.28 (4.01-10.47)	1.93 (1.54-2.42)	5.9 (2.65-9.13)	0.77 (0.6-0.98)	-0.31 (-3.67-3.19)
Organic	Migraine	Persons	3.16 (3.02-3.3)	7.92 (7.56-8.29)	3.18 (3.03-3.33)	8.29 (7.9-8.65)	1.11 (1.06-1.16)	-0.56 (-0.9--0.23)
Substance Use	Migraine	Persons	4.48 (4.31-4.67)	12.58 (11.93-13.03)	4.62 (4.43-4.82)	12.82 (12.14-13.31)	1.16 (1.11-1.21)	0.27 (-0.4-0.72)
Schizophrenia	Migraine	Persons	3.22 (3.02-3.45)	10.35 (9.32-11.12)	3.21 (3-3.44)	10.61 (9.54-11.39)	1.25 (1.17-1.34)	0.52 (-0.52-1.27)
Mood	Migraine	Persons	2.17 (2.09-2.25)	6.43 (6-6.86)	2.23 (2.14-2.32)	6.78 (6.32-7.21)	1.14 (1.1-1.18)	-0.31 (-0.76-0.14)
Neurotic	Migraine	Persons	2.41 (2.33-2.5)	6.9 (6.47-7.29)	2.51 (2.41-2.61)	7.21 (6.75-7.64)	1.14 (1.1-1.19)	0.17 (-0.3-0.59)
Eating	Migraine	Persons	3.03 (2.38-3.85)	6.04 (-3.43-10.61)	3 (2.36-3.82)	6.42 (-3.04-10.97)	1.16 (0.9-1.5)	-2.13 (-11.62-2.82)
Personality	Migraine	Persons	2.64 (2.53-2.75)	7.85 (7.04-8.37)	2.71 (2.59-2.83)	8.2 (7.4-8.71)	1.2 (1.14-1.25)	0.49 (-0.36-1.06)
Intellectual	Migraine	Persons	3.12 (2.44-4)	8.12 (-3.95-10.2)	3.09 (2.41-3.96)	8.22 (-3.86-10.28)	0.86 (0.67-1.1)	-4.36 (-16.54--2.3)
Developmental	Migraine	Persons	2.48 (1.29-4.77)	NA	2.46 (1.28-4.73)	NA	0.97 (0.5-1.89)	NA
Behavioral	Migraine	Persons	2.79 (2.34-3.34)	5.78 (-2.8-8.94)	2.77 (2.32-3.32)	5.77 (-2.9-8.92)	1.02 (0.85-1.23)	-2.71 (-11.42-0.49)
Organic	Migraine	Males	3.48 (3.18-3.82)	8.93 (8.11-9.73)	3.26 (2.96-3.58)	8.91 (8.09-9.75)	1.13 (1.03-1.24)	-0.44 (-1.19-0.32)
Substance Use	Migraine	Males	4.63 (4.32-4.96)	13.22 (12.38-13.92)	4.48 (4.16-4.83)	12.95 (12.08-13.71)	1.09 (1.01-1.17)	0.17 (-0.69-0.86)
Schizophrenia	Migraine	Males	4.15 (3.65-4.72)	12.45 (10-14.09)	3.84 (3.37-4.38)	12.09 (9.61-13.72)	1.36 (1.2-1.55)	1.24 (-1.12-2.89)
Mood	Migraine	Males	2.5 (2.31-2.71)	8.02 (7.22-8.93)	2.38 (2.19-2.59)	7.77 (6.93-8.72)	1.18 (1.09-1.28)	0.05 (-0.76-0.97)

© 2022 Momen NC et al. *JAMA Psychiatry*.

Mental disorder	General medical condition	Sex	Compared to those with neither MD nor GMC		Compared to those with GMC only		Compared to those with MD only	
			MRR	LYLs	MRR	LYLs	MRR	LYLs
Neurotic	Migraine	Males	2.74 (2.54-2.96)	8.23 (7.05-9.14)	2.62 (2.42-2.85)	7.91 (6.66-8.88)	1.12 (1.04-1.21)	-0.07 (-1.27-0.88)
Eating	Migraine	Males	NA	NA	NA	NA	NA	NA
Personality	Migraine	Males	3.03 (2.77-3.32)	9.65 (8.56-10.68)	2.87 (2.61-3.16)	9.39 (8.28-10.48)	1.2 (1.1-1.32)	0.9 (-0.21-1.98)
Intellectual	Migraine	Males	3.29 (2.04-5.29)	NA	3.01 (1.87-4.84)	NA	0.95 (0.59-1.53)	NA
Developmental	Migraine	Males	2.14 (0.89-5.15)	NA	1.96 (0.82-4.71)	NA	0.9 (0.37-2.18)	NA
Behavioral	Migraine	Males	3.34 (2.47-4.5)	7.74 (-8.22-13.46)	3.07 (2.27-4.14)	7.17 (-8.79-12.84)	1.11 (0.81-1.5)	-1.39 (-17.36-4.36)
Organic	Migraine	Females	3.05 (2.9-3.21)	7.62 (7.19-8.01)	3.16 (3-3.34)	8.11 (7.65-8.52)	1.13 (1.08-1.2)	-0.59 (-1.01--0.23)
Substance Use	Migraine	Females	4.4 (4.19-4.62)	12.25 (11.27-12.85)	4.64 (4.41-4.89)	12.75 (11.78-13.36)	1.33 (1.26-1.4)	0.32 (-0.66-0.94)
Schizophrenia	Migraine	Females	2.98 (2.76-3.22)	9.64 (8.6-10.54)	3.05 (2.82-3.3)	10.11 (9.1-11.03)	1.31 (1.21-1.42)	0.27 (-0.8-1.16)
Mood	Migraine	Females	2.08 (2-2.17)	6.09 (5.6-6.52)	2.2 (2.11-2.31)	6.57 (6.06-7)	1.17 (1.12-1.22)	-0.39 (-0.9-0.09)
Neurotic	Migraine	Females	2.32 (2.23-2.42)	6.59 (6.09-7.02)	2.49 (2.39-2.61)	7.05 (6.54-7.52)	1.23 (1.17-1.28)	0.22 (-0.29-0.69)
Eating	Migraine	Females	3 (2.35-3.83)	7.17 (-2.71-11.42)	3.06 (2.39-3.91)	7.57 (-2.34-11.85)	1.2 (0.92-1.55)	-0.93 (-10.85-3.8)
Personality	Migraine	Females	2.53 (2.41-2.66)	7.48 (6.55-8.08)	2.68 (2.54-2.82)	7.96 (7.02-8.56)	1.27 (1.21-1.34)	0.41 (-0.56-1.06)
Intellectual	Migraine	Females	3.06 (2.3-4.09)	9.2 (-4.84-11.65)	3.11 (2.33-4.16)	9.64 (-4.38-12.04)	0.8 (0.59-1.07)	-3.66 (-17.86--1.13)
Developmental	Migraine	Females	NA	NA	NA	NA	NA	NA
Behavioral	Migraine	Females	2.56 (2.05-3.2)	4.37 (-4.38-8.12)	2.61 (2.09-3.26)	4.77 (-3.95-8.52)	1.11 (0.88-1.41)	-3.66 (-12.42-0.28)
Organic	Epilepsy	Persons	5.03 (4.88-5.18)	11.19 (10.93-11.46)	1.49 (1.44-1.53)	2.94 (2.7-3.19)	1.77 (1.72-1.83)	1.82 (1.58-2.05)
Substance Use	Epilepsy	Persons	8.59 (8.3-8.89)	18.17 (17.81-18.51)	2.66 (2.56-2.76)	5.18 (4.82-5.55)	2.25 (2.17-2.33)	6.64 (6.3-6.98)
Schizophrenia	Epilepsy	Persons	5.99 (5.7-6.29)	16.64 (16.07-17.22)	1.8 (1.71-1.89)	3.21 (2.61-3.77)	2.35 (2.23-2.47)	7.35 (6.76-7.92)
Mood	Epilepsy	Persons	4.68 (4.5-4.85)	14.22 (13.8-14.64)	1.39 (1.33-1.44)	2.26 (1.83-2.69)	2.46 (2.36-2.55)	8.08 (7.66-8.5)
Neurotic	Epilepsy	Persons	5.63 (5.42-5.85)	15.95 (15.52-16.34)	1.7 (1.63-1.77)	2.1 (1.65-2.52)	2.69 (2.59-2.8)	9.28 (8.83-9.68)
Eating	Epilepsy	Persons	9.4 (7.2-12.28)	NA	2.8 (2.14-3.66)	NA	3.72 (2.81-4.92)	NA
Personality	Epilepsy	Persons	5.97 (5.7-6.25)	16.24 (15.67-16.73)	1.8 (1.71-1.88)	2.72 (2.16-3.23)	2.72 (2.6-2.85)	9.12 (8.57-9.62)
Intellectual	Epilepsy	Persons	7.34 (6.79-7.93)	17.9 (16.91-18.74)	2.21 (2.04-2.39)	1.75 (0.71-2.61)	2.24 (2.05-2.44)	5.99 (4.93-6.92)
Developmental	Epilepsy	Persons	5.85 (4.51-7.59)	13.17 (1.52-15.88)	1.74 (1.34-2.26)	-5.39 (-17.04--2.62)	2.48 (1.87-3.29)	5.07 (-6.51-8.19)

© 2022 Momen NC et al. *JAMA Psychiatry*.

Mental disorder	General medical condition	Sex	Compared to those with neither MD nor GMC		Compared to those with GMC only		Compared to those with MD only	
			MRR	LYLs	MRR	LYLs	MRR	LYLs
Behavioral	Epilepsy	Persons	8.54 (7.33-9.93)	19.4 (16.75-21.61)	2.55 (2.19-2.96)	1.23 (-1.45-3.47)	3.27 (2.79-3.84)	10.72 (8.16-12.91)
Organic	Epilepsy	Males	5.39 (5.17-5.61)	11.57 (11.25-11.93)	1.59 (1.52-1.66)	3.07 (2.76-3.37)	1.76 (1.69-1.84)	1.74 (1.43-2.05)
Substance Use	Epilepsy	Males	9.23 (8.84-9.63)	18.25 (17.82-18.69)	2.84 (2.72-2.98)	5.07 (4.63-5.52)	2.2 (2.11-2.3)	6.42 (6-6.84)
Schizophrenia	Epilepsy	Males	7.12 (6.65-7.62)	17.39 (16.65-18.14)	2.12 (1.97-2.27)	3.53 (2.77-4.29)	2.38 (2.21-2.55)	7.33 (6.6-8.11)
Mood	Epilepsy	Males	5.13 (4.85-5.42)	14.17 (13.54-14.73)	1.51 (1.42-1.6)	2.3 (1.69-2.85)	2.43 (2.29-2.57)	7.29 (6.7-7.83)
Neurotic	Epilepsy	Males	6.78 (6.42-7.17)	16.75 (16.12-17.34)	2.03 (1.92-2.15)	2.83 (2.21-3.45)	2.82 (2.66-2.98)	9.07 (8.44-9.66)
Eating	Epilepsy	Males	12.53 (5.63-27.89)	NA	3.68 (1.65-8.2)	NA	3.54 (1.53-8.2)	NA
Personality	Epilepsy	Males	6.83 (6.39-7.29)	16.34 (15.64-16.96)	2.03 (1.9-2.17)	3.01 (2.29-3.62)	2.75 (2.57-2.94)	8.38 (7.68-8.96)
Intellectual	Epilepsy	Males	6.86 (6.18-7.62)	16.78 (15.47-17.95)	2.04 (1.83-2.26)	0.34 (-0.98-1.53)	2.18 (1.94-2.45)	5.47 (4.02-6.75)
Developmental	Epilepsy	Males	5.76 (4.26-7.8)	NA	1.69 (1.25-2.29)	NA	2.63 (1.89-3.66)	NA
Behavioral	Epilepsy	Males	9.68 (8.08-11.59)	20.34 (16.92-22.89)	2.85 (2.38-3.42)	1.86 (-1.58-4.45)	3.37 (2.79-4.08)	11.25 (7.51-13.92)
Organic	Epilepsy	Females	4.69 (4.49-4.9)	10.78 (10.41-11.14)	1.39 (1.32-1.45)	2.8 (2.46-3.15)	1.74 (1.66-1.81)	1.91 (1.58-2.26)
Substance Use	Epilepsy	Females	7.61 (7.18-8.07)	18.02 (17.41-18.62)	2.36 (2.22-2.51)	5.38 (4.79-5.97)	2.28 (2.15-2.43)	7.03 (6.47-7.61)
Schizophrenia	Epilepsy	Females	5.09 (4.73-5.47)	15.86 (15.01-16.73)	1.54 (1.43-1.66)	2.87 (2-3.68)	2.24 (2.08-2.41)	7.37 (6.48-8.2)
Mood	Epilepsy	Females	4.35 (4.14-4.58)	14.25 (13.66-14.88)	1.3 (1.23-1.38)	2.23 (1.63-2.84)	2.43 (2.31-2.56)	8.65 (8.07-9.26)
Neurotic	Epilepsy	Females	4.87 (4.62-5.13)	15.36 (14.72-15.97)	1.48 (1.4-1.57)	1.56 (0.87-2.19)	2.55 (2.42-2.7)	9.44 (8.84-10.06)
Eating	Epilepsy	Females	9.11 (6.87-12.1)	NA	2.76 (2.08-3.66)	NA	3.74 (2.78-5.02)	NA
Personality	Epilepsy	Females	5.31 (4.98-5.67)	16.17 (15.39-16.92)	1.62 (1.52-1.74)	2.52 (1.66-3.27)	2.66 (2.49-2.84)	9.66 (8.88-10.39)
Intellectual	Epilepsy	Females	8.01 (7.14-8.99)	19.4 (18.05-20.72)	2.45 (2.18-2.75)	3.65 (2.3-4.97)	2.32 (2.04-2.64)	6.69 (5.16-8.11)
Developmental	Epilepsy	Females	6.12 (3.69-10.15)	NA	1.85 (1.11-3.07)	NA	2.04 (1.18-3.55)	NA
Behavioral	Epilepsy	Females	6.66 (5.03-8.81)	17.8 (14.32-21.73)	2.01 (1.52-2.67)	0.16 (-3.38-4.13)	2.96 (2.21-3.95)	9.83 (6.19-13.72)
Organic	Parkinson's disease	Persons	4.92 (4.75-5.09)	6.96 (6.78-7.12)	1.87 (1.8-1.94)	2.26 (2.11-2.42)	1.73 (1.67-1.79)	1.63 (1.5-1.77)
Substance Use	Parkinson's disease	Persons	4.37 (4.02-4.75)	9.76 (9.08-10.37)	1.61 (1.48-1.75)	3.03 (2.42-3.6)	1.11 (1.02-1.21)	3.21 (2.62-3.75)
Schizophrenia	Parkinson's disease	Persons	3.51 (3.27-3.78)	8.4 (7.88-8.98)	1.31 (1.22-1.41)	1.91 (1.41-2.46)	1.35 (1.26-1.46)	3.15 (2.68-3.67)
Mood	Parkinson's disease	Persons	3.26 (3.13-3.41)	6.75 (6.47-7.05)	1.21 (1.15-1.27)	1.17 (0.89-1.43)	1.71 (1.63-1.79)	3.48 (3.21-3.76)
Neurotic	Parkinson's disease	Persons	3.32 (3.12-3.54)	7.85 (7.35-8.32)	1.23 (1.16-1.32)	1.4 (0.92-1.86)	1.56 (1.46-1.66)	4.21 (3.73-4.68)

© 2022 Momen NC et al. *JAMA Psychiatry*.

Mental disorder	General medical condition	Sex	Compared to those with neither MD nor GMC		Compared to those with GMC only		Compared to those with MD only	
			MRR	LYLs	MRR	LYLs	MRR	LYLs
Eating	Parkinson's disease	Persons	NA	NA	NA	NA	NA	NA
Personality	Parkinson's disease	Persons	3.43 (3.16-3.73)	8.13 (7.46-8.78)	1.28 (1.18-1.39)	1.45 (0.77-2.06)	1.53 (1.41-1.66)	4.24 (3.61-4.88)
Intellectual	Parkinson's disease	Persons	5.03 (4.01-6.32)	NA	1.89 (1.5-2.37)	NA	1.39 (1.1-1.75)	NA
Developmental	Parkinson's disease	Persons	NA	NA	NA	NA	NA	NA
Behavioral	Parkinson's disease	Persons	3.09 (1.99-4.79)	NA	1.16 (0.75-1.8)	NA	1.13 (0.73-1.75)	NA
Organic	Parkinson's disease	Males	5.17 (4.94-5.41)	6.99 (6.78-7.21)	2 (1.91-2.11)	2.43 (2.24-2.64)	1.69 (1.61-1.77)	1.61 (1.42-1.77)
Substance Use	Parkinson's disease	Males	4.72 (4.23-5.27)	9.7 (8.92-10.54)	1.73 (1.55-1.94)	3 (2.25-3.78)	1.09 (0.98-1.22)	2.87 (2.17-3.59)
Schizophrenia	Parkinson's disease	Males	4.21 (3.78-4.69)	9.33 (8.44-10.11)	1.59 (1.42-1.77)	2.74 (1.9-3.47)	1.37 (1.23-1.53)	3.57 (2.8-4.28)
Mood	Parkinson's disease	Males	3.25 (3.04-3.47)	6.82 (6.36-7.27)	1.21 (1.13-1.3)	1.35 (0.92-1.77)	1.52 (1.42-1.63)	3.24 (2.81-3.66)
Neurotic	Parkinson's disease	Males	3.2 (2.89-3.53)	7.9 (7.1-8.66)	1.19 (1.08-1.32)	1.54 (0.83-2.24)	1.29 (1.17-1.43)	3.77 (3.01-4.51)
Eating	Parkinson's disease	Males	NA	NA	NA	NA	NA	NA
Personality	Parkinson's disease	Males	3.77 (3.33-4.27)	8.2 (7.23-9.16)	1.42 (1.25-1.6)	1.69 (0.72-2.58)	1.48 (1.31-1.68)	3.98 (3-4.84)
Intellectual	Parkinson's disease	Males	5.52 (4.14-7.37)	NA	2.09 (1.56-2.79)	NA	1.59 (1.19-2.14)	NA
Developmental	Parkinson's disease	Males	NA	NA	NA	NA	NA	NA
Behavioral	Parkinson's disease	Males	6.76 (3.22-14.17)	NA	2.55 (1.22-5.36)	NA	2.22 (1.06-4.68)	NA
Organic	Parkinson's disease	Females	4.62 (4.38-4.88)	6.92 (6.64-7.17)	1.71 (1.61-1.81)	2.04 (1.79-2.28)	1.71 (1.62-1.8)	1.66 (1.43-1.87)
Substance Use	Parkinson's disease	Females	4 (3.53-4.53)	9.84 (8.71-10.83)	1.47 (1.3-1.67)	3.07 (1.99-3.94)	1.17 (1.03-1.32)	3.71 (2.7-4.54)
Schizophrenia	Parkinson's disease	Females	3.07 (2.78-3.39)	7.58 (6.85-8.35)	1.13 (1.02-1.25)	1.18 (0.51-1.89)	1.33 (1.2-1.47)	2.79 (2.08-3.51)
Mood	Parkinson's disease	Females	3.27 (3.09-3.47)	6.71 (6.32-7.08)	1.2 (1.13-1.28)	1.03 (0.65-1.4)	1.83 (1.72-1.94)	3.67 (3.29-4.02)
Neurotic	Parkinson's disease	Females	3.42 (3.14-3.71)	7.82 (7.2-8.42)	1.26 (1.15-1.37)	1.31 (0.64-1.89)	1.77 (1.62-1.92)	4.52 (3.89-5.14)
Eating	Parkinson's disease	Females	NA	NA	NA	NA	NA	NA
Personality	Parkinson's disease	Females	3.2 (2.87-3.57)	8.07 (7.13-8.93)	1.18 (1.05-1.32)	1.27 (0.33-2.1)	1.57 (1.4-1.76)	4.44 (3.52-5.29)
Intellectual	Parkinson's disease	Females	4.39 (3.03-6.36)	NA	1.63 (1.13-2.37)	NA	1.14 (0.79-1.66)	NA
Developmental	Parkinson's disease	Females	NA	NA	NA	NA	NA	NA
Behavioral	Parkinson's disease	Females	2.39 (1.39-4.12)	NA	0.89 (0.52-1.53)	NA	1.02 (0.59-1.77)	NA
Organic	Multiple sclerosis	Persons	5.61 (5-6.3)	13.2 (12.17-14.27)	2.01 (1.78-2.27)	6.35 (5.36-7.37)	1.97 (1.76-2.21)	1.81 (0.89-2.73)
Substance Use	Multiple sclerosis	Persons	7.46 (6.4-8.69)	16.07 (13.25-17.6)	2.58 (2.21-3.01)	7.32 (4.52-8.82)	1.91 (1.64-2.22)	3.59 (0.8-5.03)

© 2022 Momen NC et al. *JAMA Psychiatry*.

Mental disorder	General medical condition	Sex	Compared to those with neither MD nor GMC		Compared to those with GMC only		Compared to those with MD only	
			MRR	LYLs	MRR	LYLs	MRR	LYLs
Schizophrenia	Multiple sclerosis	Persons	5.64 (4.64-6.87)	12.5 (6.92-15.05)	2 (1.64-2.44)	3.5 (-2.15-6.11)	2.18 (1.79-2.65)	2.5 (-3.1-5.01)
Mood	Multiple sclerosis	Persons	4.83 (4.31-5.42)	13.28 (11.84-14.44)	1.72 (1.53-1.94)	4.65 (3.19-5.81)	2.52 (2.25-2.83)	6.35 (4.9-7.5)
Neurotic	Multiple sclerosis	Persons	5.05 (4.5-5.67)	11.79 (8.52-13.18)	1.8 (1.6-2.03)	2.81 (-0.37-4.25)	2.37 (2.11-2.66)	4.89 (1.62-6.29)
Eating	Multiple sclerosis	Persons	NA	NA	NA	NA	NA	NA
Personality	Multiple sclerosis	Persons	4.58 (3.93-5.33)	9.91 (4.41-12.44)	1.62 (1.39-1.89)	1 (-4.39-3.63)	2.05 (1.76-2.39)	2.42 (-3.1-4.99)
Intellectual	Multiple sclerosis	Persons	NA	NA	NA	NA	NA	NA
Developmental	Multiple sclerosis	Persons	NA	NA	NA	NA	NA	NA
Behavioral	Multiple sclerosis	Persons	3.93 (1.87-8.25)	NA	1.4 (0.67-2.95)	NA	1.44 (0.68-3.02)	NA
Organic	Multiple sclerosis	Males	6.26 (5.22-7.51)	NA	2.23 (1.85-2.7)	NA	2.04 (1.7-2.44)	NA
Substance Use	Multiple sclerosis	Males	8.49 (6.83-10.56)	NA	2.89 (2.31-3.61)	NA	1.98 (1.59-2.47)	NA
Schizophrenia	Multiple sclerosis	Males	6.01 (4.41-8.2)	NA	2.11 (1.54-2.88)	NA	1.97 (1.44-2.68)	NA
Mood	Multiple sclerosis	Males	5.28 (4.35-6.4)	NA	1.87 (1.53-2.28)	NA	2.48 (2.05-3.01)	NA
Neurotic	Multiple sclerosis	Males	6.8 (5.57-8.3)	NA	2.4 (1.96-2.95)	NA	2.77 (2.27-3.38)	NA
Eating	Multiple sclerosis	Males	NA	NA	NA	NA	NA	NA
Personality	Multiple sclerosis	Males	5.67 (4.43-7.26)	NA	1.99 (1.55-2.56)	NA	2.24 (1.75-2.87)	NA
Intellectual	Multiple sclerosis	Males	NA	NA	NA	NA	NA	NA
Developmental	Multiple sclerosis	Males	NA	NA	NA	NA	NA	NA
Behavioral	Multiple sclerosis	Males	NA	NA	NA	NA	NA	NA
Organic	Multiple sclerosis	Females	5.23 (4.5-6.07)	12.87 (11.6-14.36)	1.88 (1.61-2.2)	6.06 (4.84-7.41)	1.94 (1.67-2.25)	1.53 (0.3-2.72)
Substance Use	Multiple sclerosis	Females	6.68 (5.39-8.27)	14.73 (10.68-17.24)	2.34 (1.88-2.91)	6.22 (2.12-8.7)	1.96 (1.58-2.43)	2.83 (-1.16-5.3)
Schizophrenia	Multiple sclerosis	Females	5.42 (4.21-6.98)	12.26 (5.65-15.67)	1.94 (1.5-2.5)	3.38 (-3.13-6.77)	2.35 (1.83-3.03)	2.81 (-3.87-6.2)
Mood	Multiple sclerosis	Females	4.6 (3.99-5.32)	13.27 (11.65-14.66)	1.65 (1.42-1.92)	4.63 (3.04-6.07)	2.56 (2.22-2.96)	6.74 (5.07-8.08)
Neurotic	Multiple sclerosis	Females	4.46 (3.87-5.14)	10.83 (7.05-12.65)	1.6 (1.38-1.85)	1.88 (-1.96-3.75)	2.31 (2-2.66)	4.38 (0.56-6.18)
Eating	Multiple sclerosis	Females	NA	NA	NA	NA	NA	NA
Personality	Multiple sclerosis	Females	4.08 (3.36-4.96)	9.22 (2.07-12.6)	1.45 (1.19-1.77)	0.3 (-6.93-3.61)	2.01 (1.65-2.44)	2.13 (-5.09-5.45)
Intellectual	Multiple sclerosis	Females	NA	NA	NA	NA	NA	NA
Developmental	Multiple sclerosis	Females	NA	NA	NA	NA	NA	NA

© 2022 Momen NC et al. *JAMA Psychiatry*.

Mental disorder	General medical condition	Sex	Compared to those with neither MD nor GMC		Compared to those with GMC only		Compared to those with MD only	
			MRR	LYLs	MRR	LYLs	MRR	LYLs
Behavioral	Multiple sclerosis	Females	NA	NA	NA	NA	NA	NA
Organic	Neuropathies	Persons	3.24 (3.14-3.35)	7.19 (6.96-7.43)	2.43 (2.36-2.52)	6.44 (6.22-6.69)	1.13 (1.09-1.16)	0.37 (0.15-0.56)
Substance Use	Neuropathies	Persons	5.35 (5.19-5.5)	13.5 (13.18-13.77)	4.25 (4.13-4.38)	10.76 (10.45-11.03)	1.4 (1.35-1.44)	2.66 (2.35-2.94)
Schizophrenia	Neuropathies	Persons	3.6 (3.42-3.8)	11.88 (11.32-12.47)	2.76 (2.62-2.91)	9.52 (8.96-10.11)	1.39 (1.32-1.47)	3.32 (2.74-3.92)
Mood	Neuropathies	Persons	2.4 (2.33-2.47)	7.72 (7.42-8.01)	1.85 (1.8-1.91)	5.82 (5.51-6.12)	1.25 (1.22-1.29)	1.82 (1.5-2.12)
Neurotic	Neuropathies	Persons	2.81 (2.72-2.89)	8.47 (8.13-8.8)	2.19 (2.12-2.26)	6.23 (5.88-6.56)	1.33 (1.29-1.37)	2.24 (1.89-2.57)
Eating	Neuropathies	Persons	4.26 (3.26-5.56)	7.52 (-4.24-11.87)	3.26 (2.5-4.26)	5.61 (-6.21-10.01)	1.64 (1.24-2.17)	-0.41 (-12.12-4.11)
Personality	Neuropathies	Persons	3.08 (2.96-3.2)	9.19 (8.69-9.61)	2.38 (2.29-2.48)	7 (6.5-7.44)	1.39 (1.34-1.45)	2.53 (2.01-2.96)
Intellectual	Neuropathies	Persons	4.24 (3.53-5.1)	NA	3.25 (2.7-3.91)	NA	1.16 (0.96-1.4)	NA
Developmental	Neuropathies	Persons	5.73 (3.33-9.87)	NA	4.39 (2.55-7.57)	NA	2.27 (1.31-3.95)	NA
Behavioral	Neuropathies	Persons	3.7 (3.08-4.44)	8.98 (3-11.37)	2.84 (2.36-3.41)	5.45 (-0.57-7.88)	1.36 (1.13-1.65)	0.57 (-5.47-3.04)
Organic	Neuropathies	Males	3.63 (3.47-3.8)	8.15 (7.8-8.49)	2.41 (2.29-2.53)	6.55 (6.22-6.9)	1.16 (1.11-1.22)	0.62 (0.31-0.91)
Substance Use	Neuropathies	Males	5.8 (5.6-6.01)	13.9 (13.55-14.26)	4.09 (3.94-4.25)	10.23 (9.86-10.61)	1.38 (1.33-1.43)	2.88 (2.51-3.22)
Schizophrenia	Neuropathies	Males	4.19 (3.89-4.51)	12.85 (12.06-13.62)	2.82 (2.62-3.05)	9.1 (8.32-9.85)	1.37 (1.27-1.48)	3.64 (2.84-4.37)
Mood	Neuropathies	Males	2.87 (2.74-2.99)	9.16 (8.77-9.61)	1.95 (1.87-2.04)	5.91 (5.5-6.38)	1.35 (1.29-1.42)	2.67 (2.27-3.12)
Neurotic	Neuropathies	Males	3.38 (3.23-3.54)	10.08 (9.62-10.52)	2.32 (2.22-2.43)	6.33 (5.86-6.79)	1.39 (1.33-1.46)	3.05 (2.57-3.53)
Eating	Neuropathies	Males	6.05 (2.52-14.54)	NA	4.08 (1.7-9.81)	NA	1.66 (0.67-4.13)	NA
Personality	Neuropathies	Males	3.7 (3.5-3.91)	10.62 (9.94-11.21)	2.52 (2.38-2.67)	6.95 (6.26-7.52)	1.48 (1.39-1.57)	3.28 (2.58-3.87)
Intellectual	Neuropathies	Males	5.37 (4.21-6.85)	NA	3.62 (2.84-4.62)	NA	1.54 (1.2-1.97)	NA
Developmental	Neuropathies	Males	8.22 (4.55-14.84)	NA	5.55 (3.07-10.02)	NA	3.51 (1.92-6.42)	NA
Behavioral	Neuropathies	Males	4.3 (3.43-5.4)	9.41 (-0.24-13.5)	2.91 (2.31-3.65)	4.4 (-5.25-8.46)	1.43 (1.13-1.8)	0.57 (-9.16-4.49)
Organic	Neuropathies	Females	2.96 (2.84-3.09)	6.39 (6.1-6.68)	2.52 (2.41-2.64)	6.35 (6.07-6.67)	1.09 (1.04-1.14)	0.16 (-0.1-0.44)
Substance Use	Neuropathies	Females	4.66 (4.44-4.9)	12.83 (12.22-13.35)	4.15 (3.95-4.37)	11.62 (11.04-12.14)	1.4 (1.33-1.48)	2.31 (1.71-2.83)
Schizophrenia	Neuropathies	Females	3.16 (2.93-3.4)	11.04 (10.25-11.86)	2.75 (2.55-2.96)	9.87 (9.1-10.7)	1.38 (1.28-1.49)	3.04 (2.25-3.86)
Mood	Neuropathies	Females	2.1 (2.02-2.19)	6.86 (6.43-7.25)	1.85 (1.77-1.93)	5.77 (5.33-6.19)	1.17 (1.13-1.22)	1.31 (0.88-1.72)
Neurotic	Neuropathies	Females	2.43 (2.33-2.54)	7.49 (7.05-7.94)	2.16 (2.07-2.26)	6.17 (5.67-6.61)	1.27 (1.22-1.33)	1.74 (1.27-2.21)

© 2022 Momen NC et al. *JAMA Psychiatry*.

Mental disorder	General medical condition	Sex	Compared to those with neither MD nor GMC		Compared to those with GMC only		Compared to those with MD only	
			MRR	LYLs	MRR	LYLs	MRR	LYLs
Eating	Neuropathies	Females	4.1 (3.1-5.43)	7.64 (-4.5-12.31)	3.56 (2.69-4.72)	5.85 (-6.26-10.59)	1.65 (1.23-2.22)	-0.21 (-12.32-4.69)
Personality	Neuropathies	Females	2.64 (2.5-2.79)	8.34 (7.68-8.93)	2.33 (2.2-2.46)	7.03 (6.35-7.66)	1.32 (1.24-1.39)	2.08 (1.4-2.71)
Intellectual	Neuropathies	Females	3.3 (2.49-4.38)	NA	2.86 (2.16-3.8)	NA	0.85 (0.64-1.14)	NA
Developmental	Neuropathies	Females	NA	NA	NA	NA	NA	NA
Behavioral	Neuropathies	Females	2.95 (2.17-4.01)	8.48 (3.05-11.19)	2.57 (1.89-3.49)	6.69 (1.32-9.41)	1.28 (0.93-1.76)	0.57 (-4.75-3.46)

© 2022 Momen NC et al. *JAMA Psychiatry*.

eFigures 1A-1J. Comparison of mortality rate ratios (MRRs) and life years lost (LYLs) for pairs of mental disorder and general medical conditions, for all persons

The left panels show MRRs, with 95% CIs, for all persons with a diagnosis of both the mental disorder and GMC of interest compared to those i) with neither the mental disorder nor the GMC (MD- GMC-; green), ii) with the GMC only (MD- GMC-; purple), and iii) with the mental disorder only (MD+ GMC-; orange). All MRRs were adjusted for age, sex and calendar time.

The right panels show LYLs (the reduction in life expectancy) for all persons with a diagnosis of both the mental disorder and the GMC of interest compared to those i) in the general population (green), ii) with GMCs, regardless of mental disorder status (purple), and iii) with mental disorders, regardless of GMC status (orange). This is calculated for people of the same sex alive at ages corresponding to the age-of-onset distribution for those with the mental disorder-general medical condition.

MRRs are shown on a log scale. Estimates are not displayed where numbers do not meet requirements for reporting of Danish register data. Narrow 95% CIs may not be visible.

© 2022 Momen NC et al. *JAMA Psychiatry*.

eFigure 1A. Organic disorders

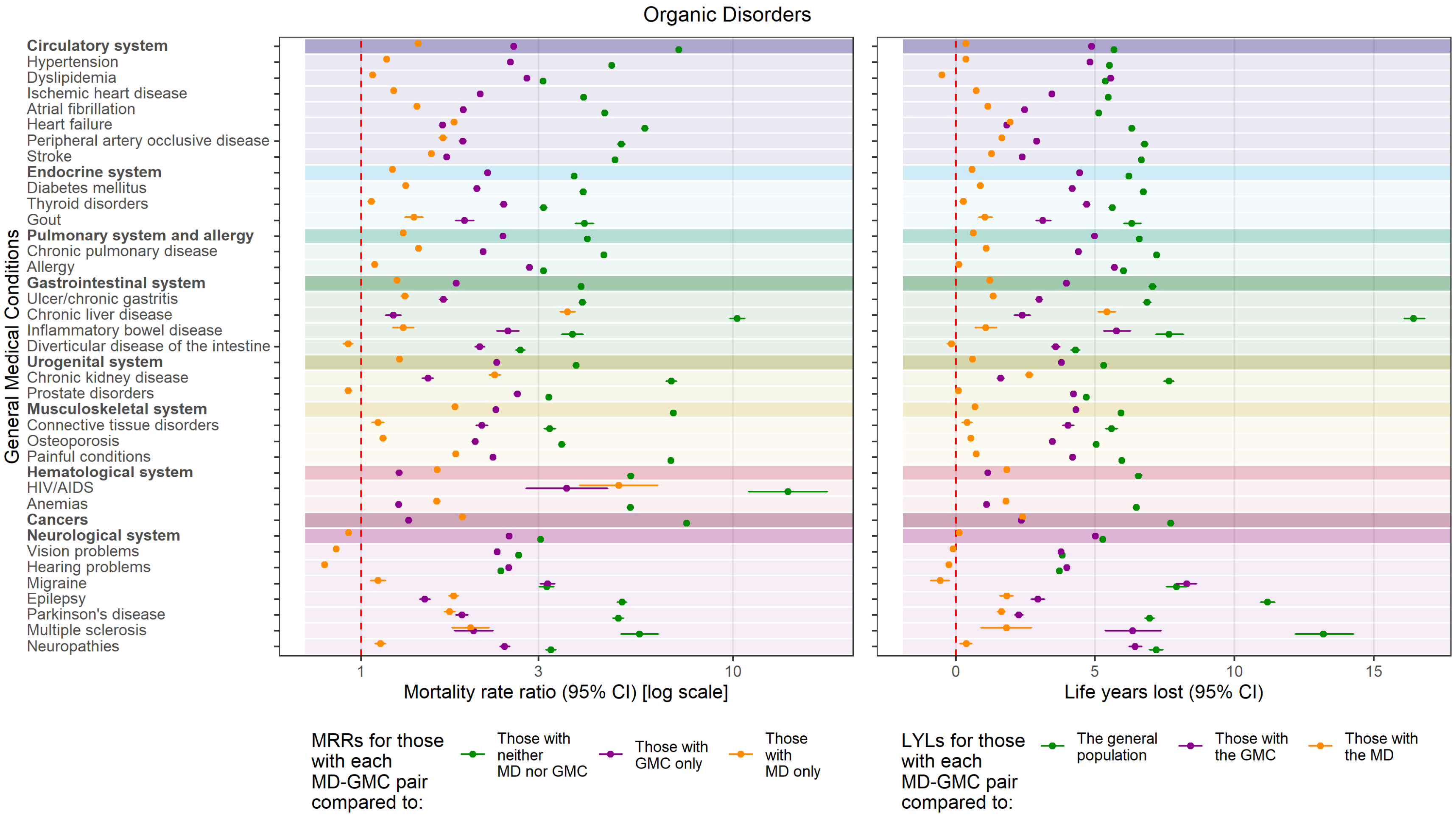

© 2022 Momen NC et al. *JAMA Psychiatry*.

eFigure 1B. Substance use disorders

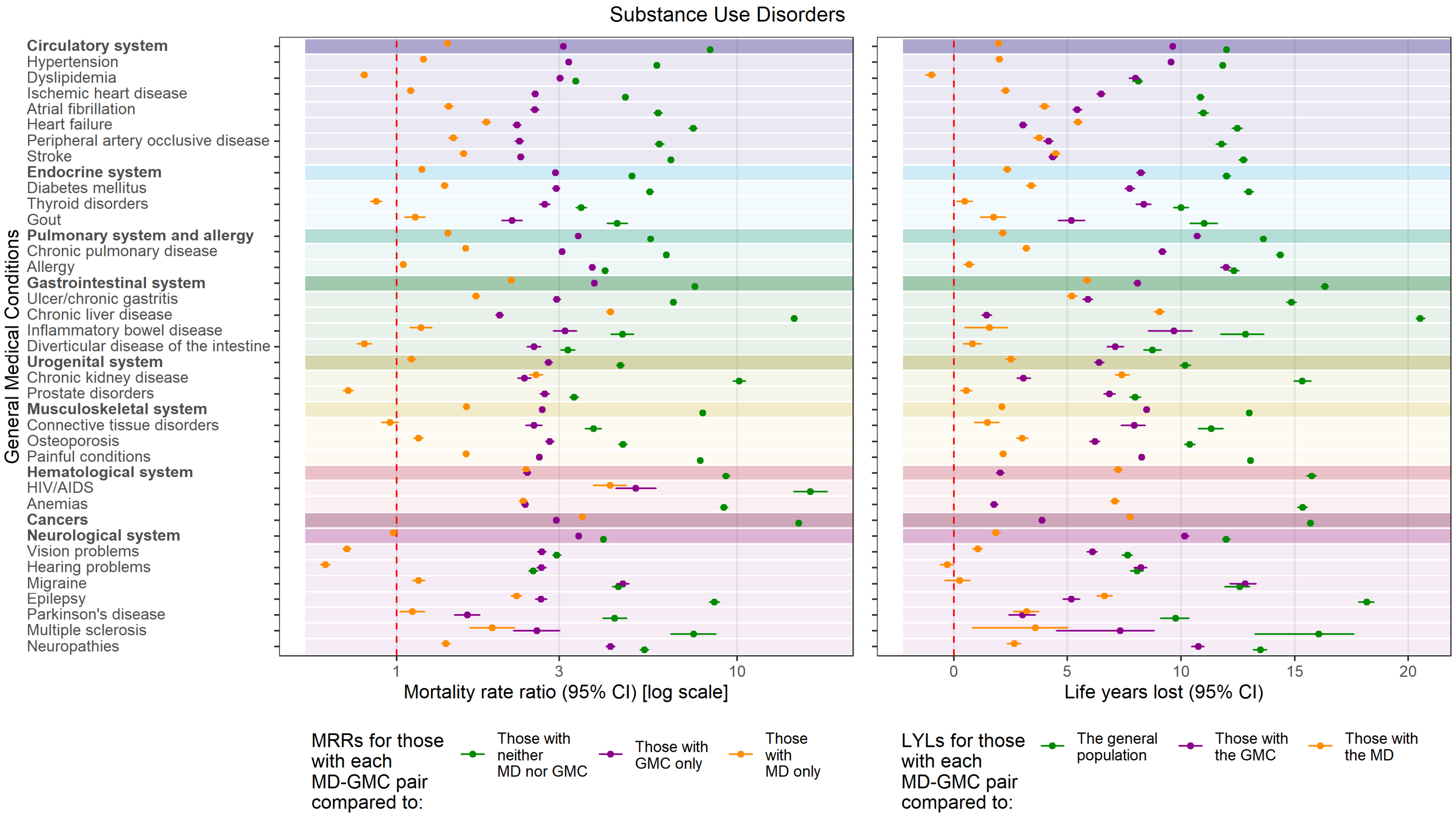

© 2022 Momen NC et al. *JAMA Psychiatry*.

eFigure 1C. Schizophrenia

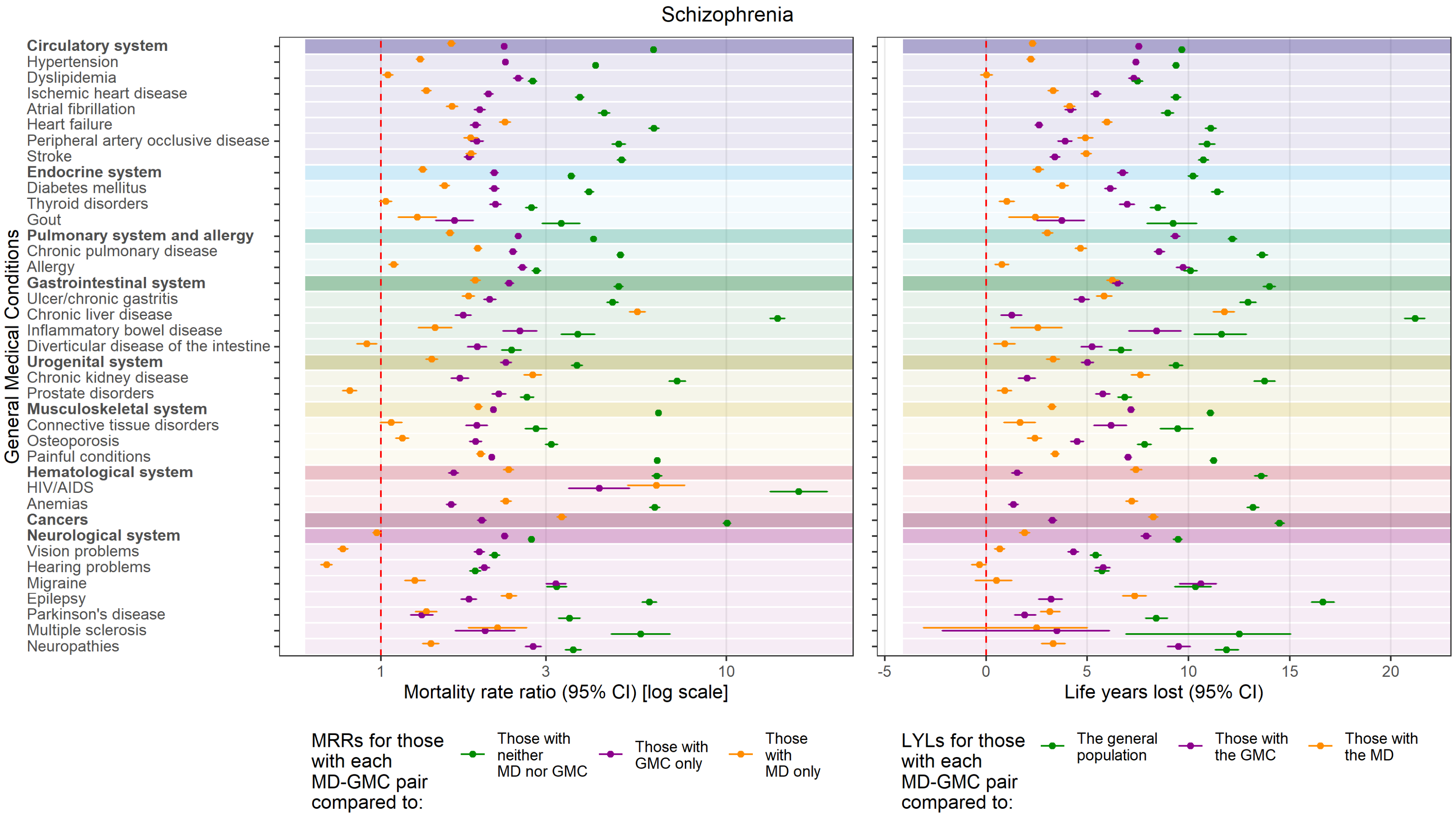

© 2022 Momen NC et al. *JAMA Psychiatry*.

eFigure 1D. Mood disorders

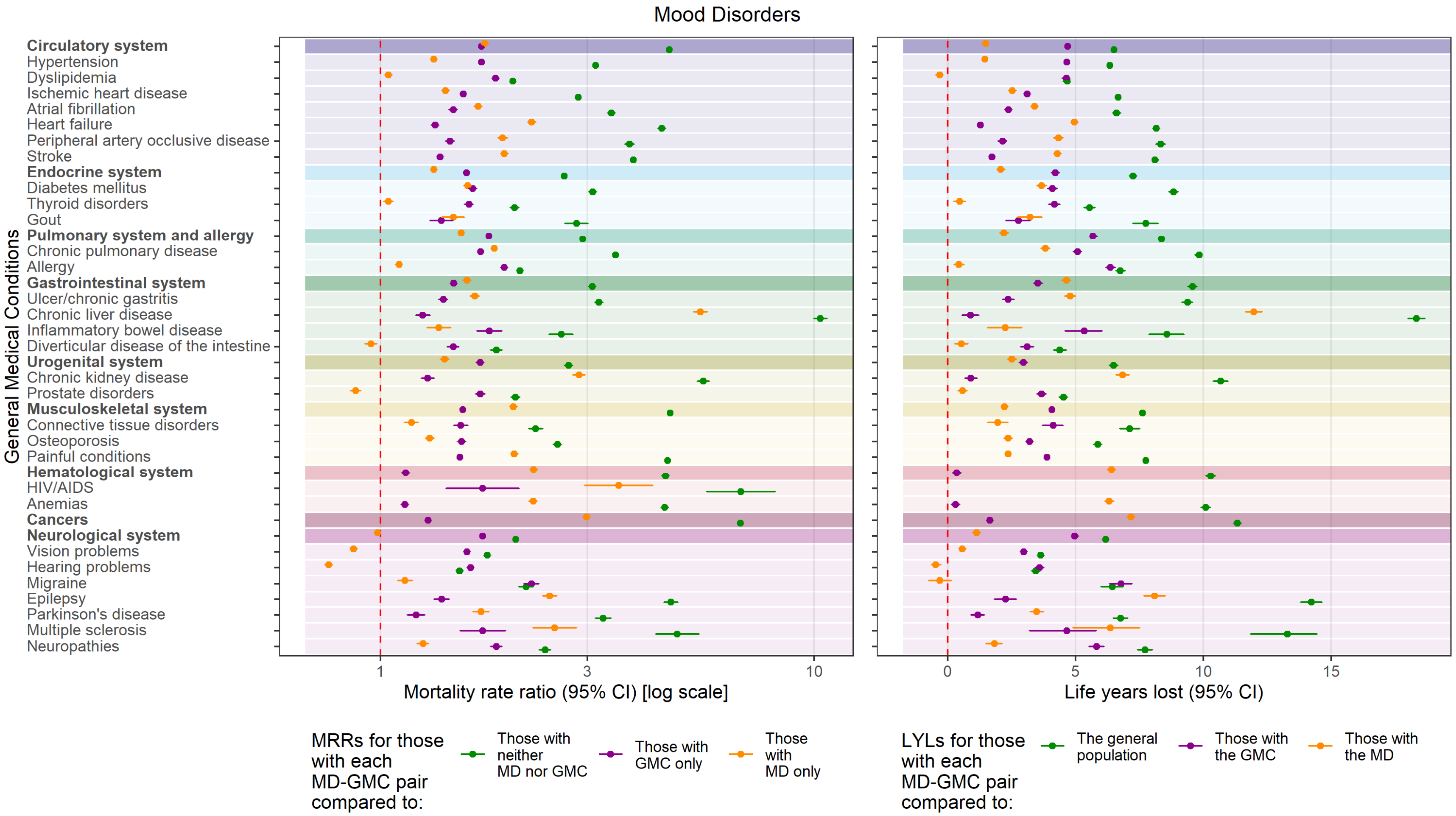

© 2022 Momen NC et al. *JAMA Psychiatry*.

eFigure 1E. Neurotic disorders

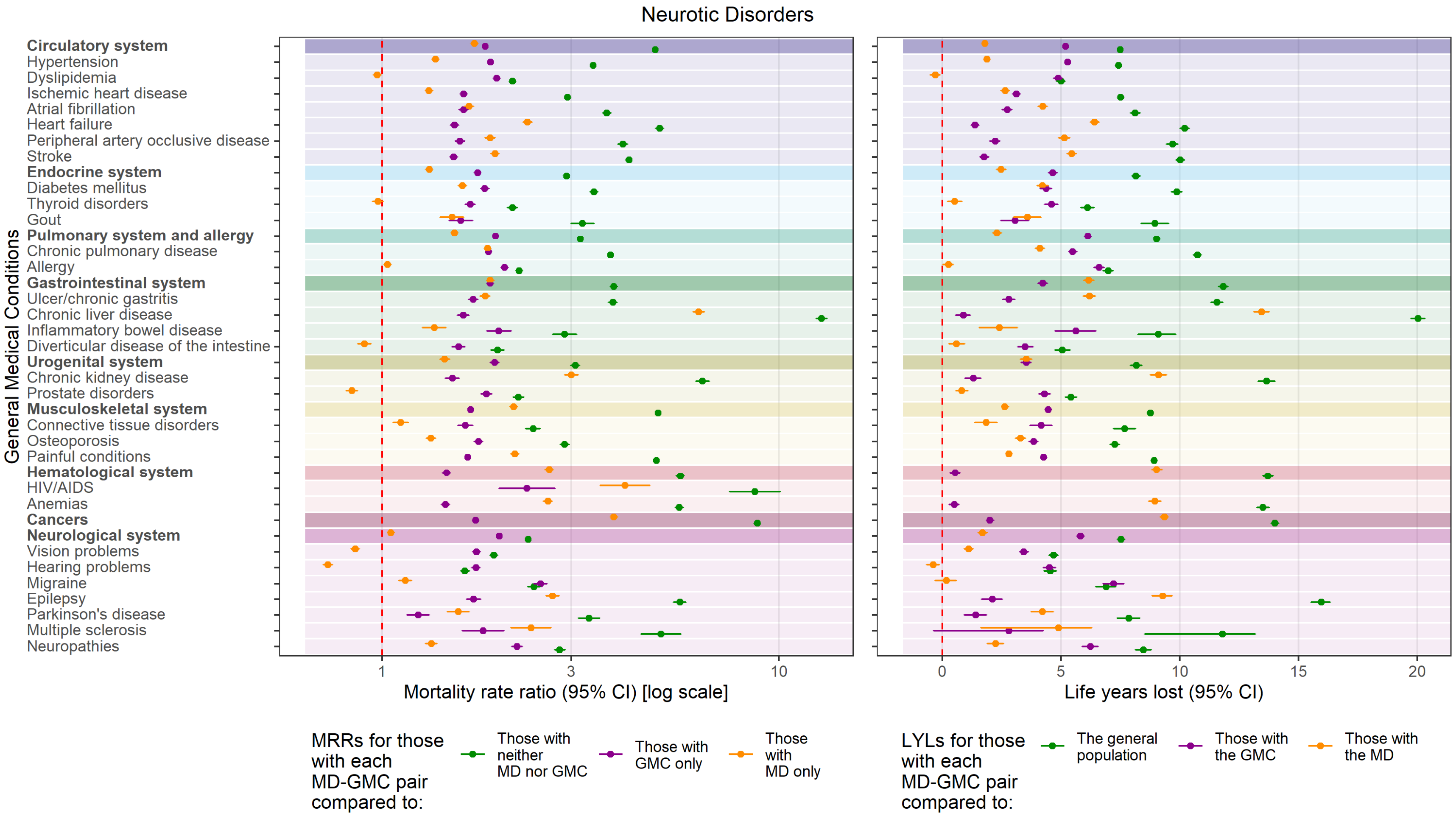

© 2022 Momen NC et al. *JAMA Psychiatry*.

eFigure 1F. Eating disorders

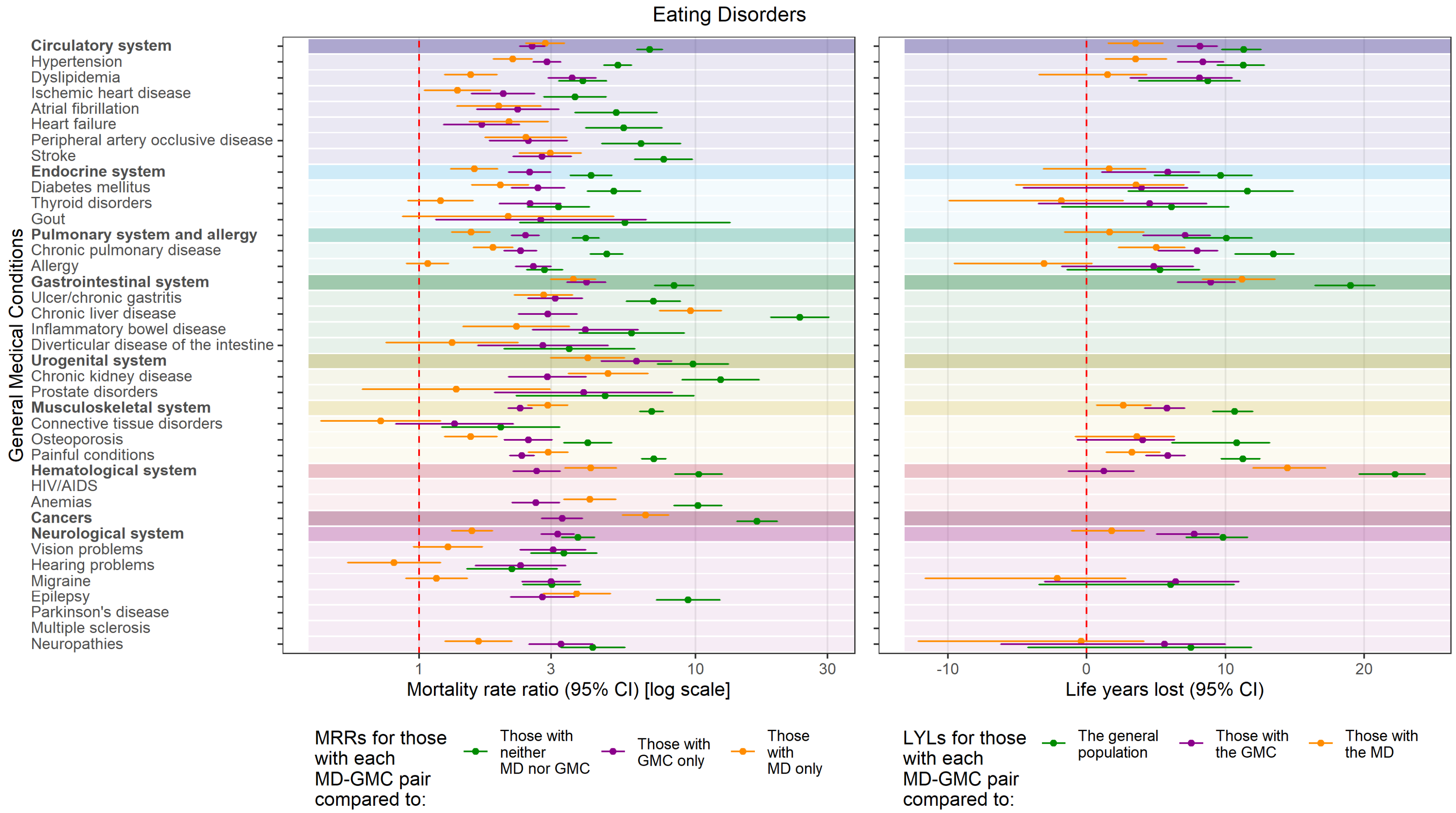

© 2022 Momen NC et al. *JAMA Psychiatry*.

eFigure 1G. Personality disorders

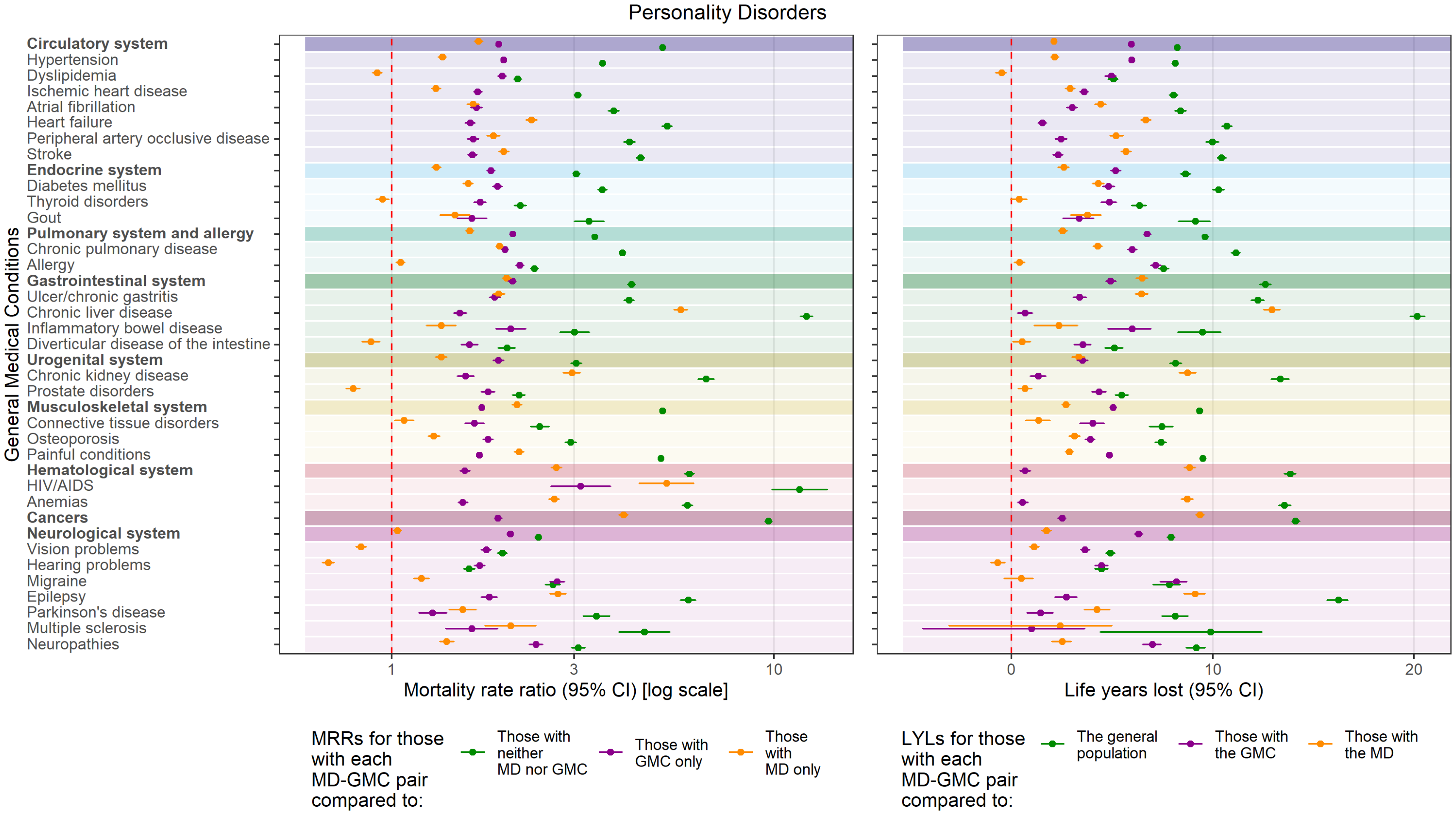

© 2022 Momen NC et al. *JAMA Psychiatry*.

eFigure 1H. Intellectual disabilities

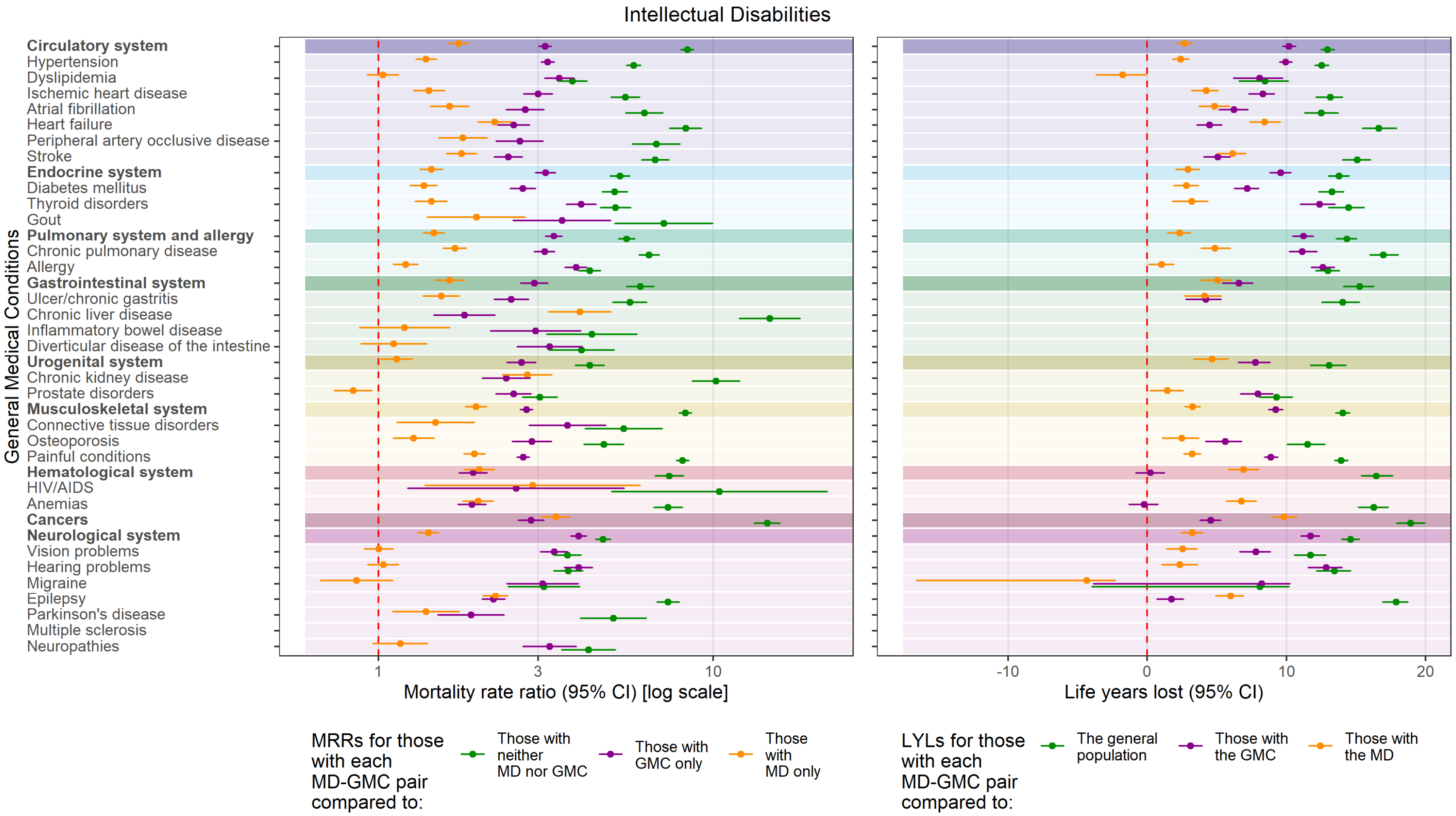

© 2022 Momen NC et al. *JAMA Psychiatry*.

eFigure 1l. Developmental disorders

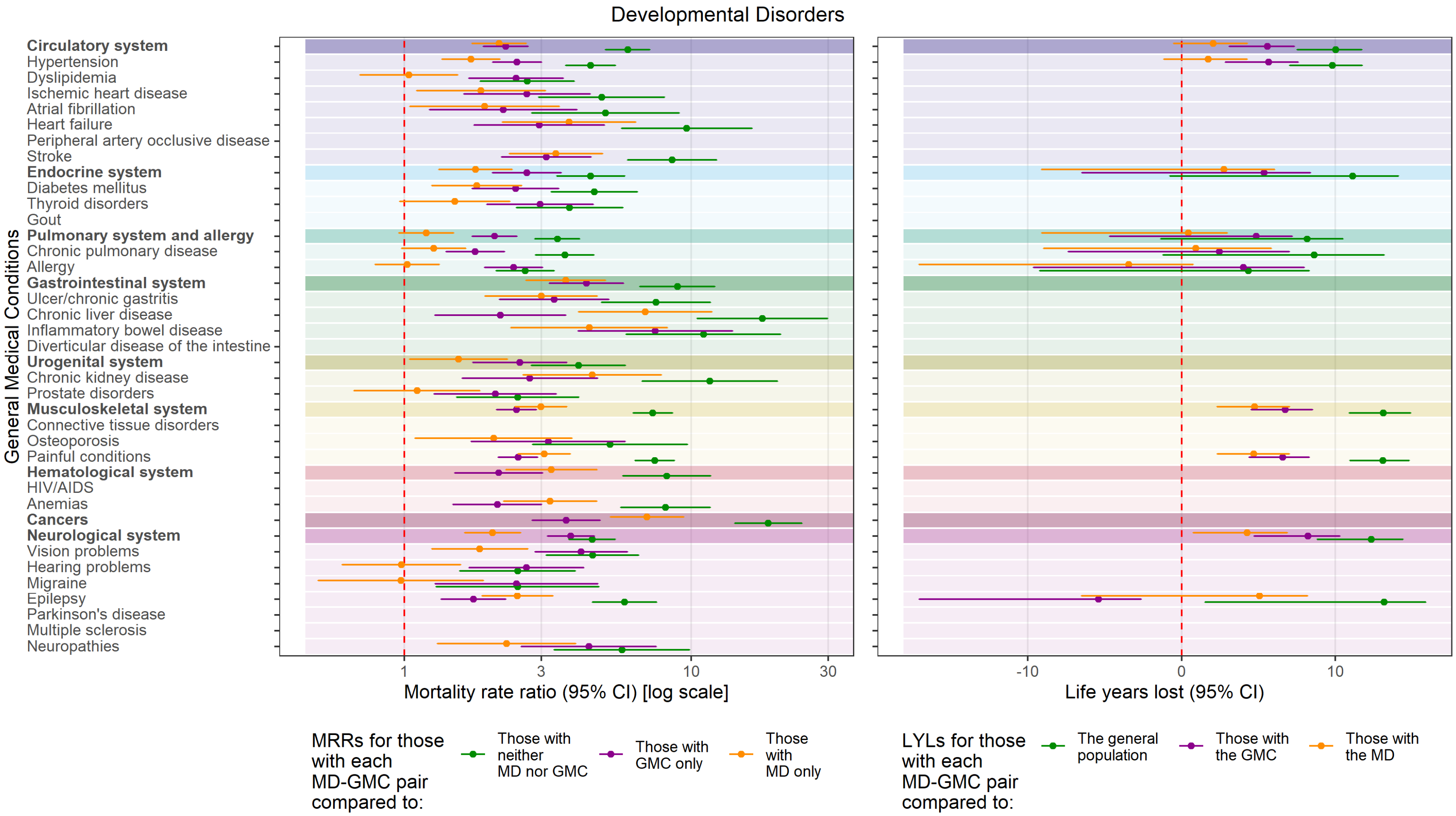

© 2022 Momen NC et al. *JAMA Psychiatry*.

eFigure 1J. Behavioral disorders

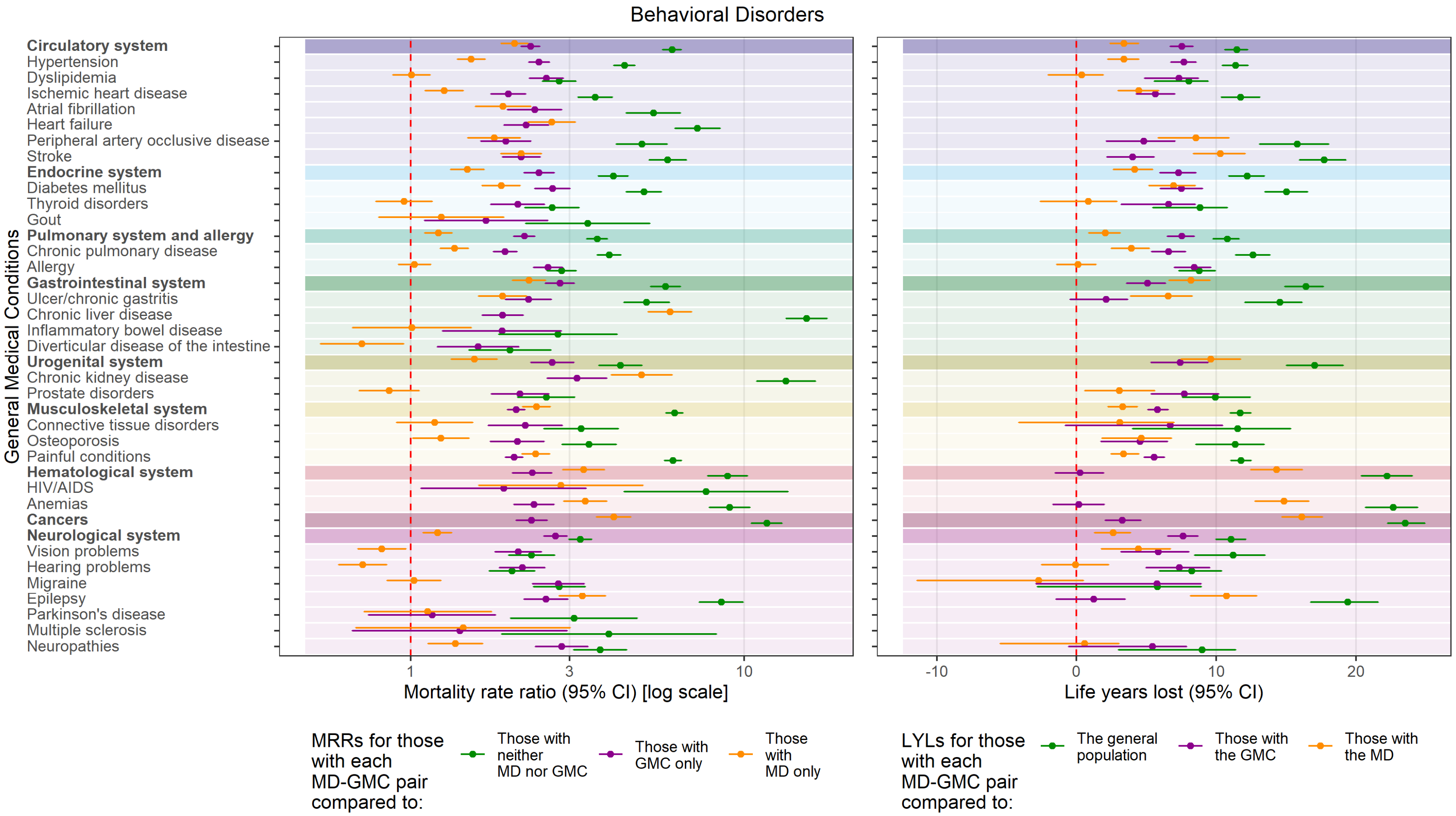

© 2022 Momen NC et al. *JAMA Psychiatry*.

eFigures 2A-2J. Sex-specific comparison of mortality rate ratios (MRRs) and life years lost (LYLs) for pairs of mental disorder and general medical conditions

The left panels show MRRs, with 95% CIs, for females (triangles) and males (circles) with a diagnosis of both the mental disorder and GMC of interest compared to those in the respective sex i) with neither the mental disorder nor the GMC (MD- GMC-; green), ii) with the GMC only (MD- GMC-; purple), and iii) with the mental disorder only (MD+ GMC-; orange). All MRRs were adjusted for age and calendar time.

The right panels show LYLs (the reduction in life expectancy) for females (triangles) and males (circles) with a diagnosis of both the mental disorder and the GMC of interest compared to those in the respective sex i) in the general population (green), ii) with GMCs, regardless of mental disorder status (purple), and iii) with mental disorders, regardless of GMC status (orange). This is calculated for people of the same sex alive at ages corresponding to the age-of-onset distribution for those with the mental disorder-general medical condition.

MRRs are shown on a log scale. Estimates are not displayed where numbers do not meet requirements for reporting of Danish register data. Narrow 95% CIs may not be visible.

© 2022 Momen NC et al. *JAMA Psychiatry*.

eFigure 2A. Organic disorders

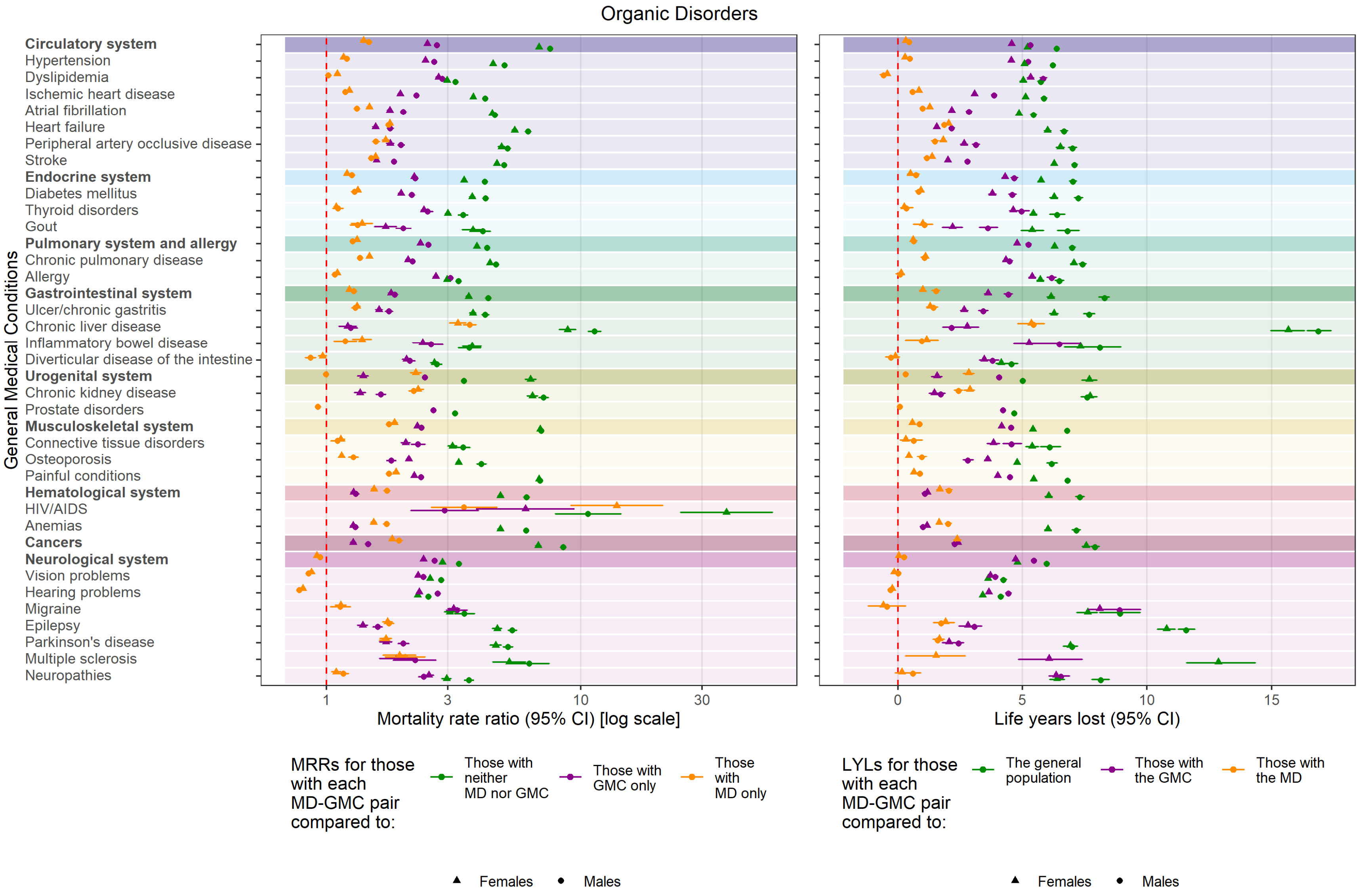

© 2022 Momen NC et al. *JAMA Psychiatry*.

eFigure 2B. Substance-use disorders

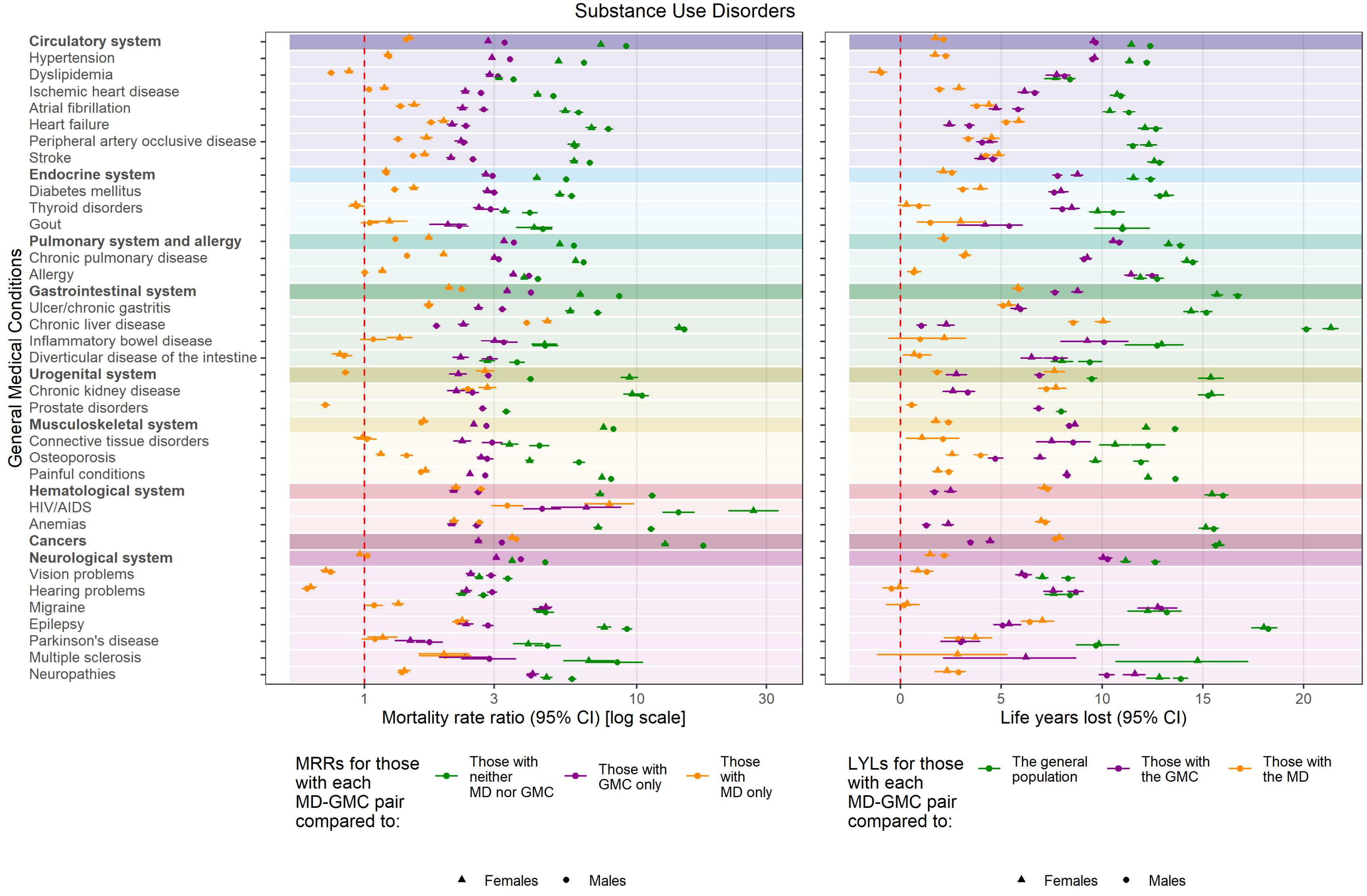

© 2022 Momen NC et al. *JAMA Psychiatry*.

eFigure 2C. Schizophrenia

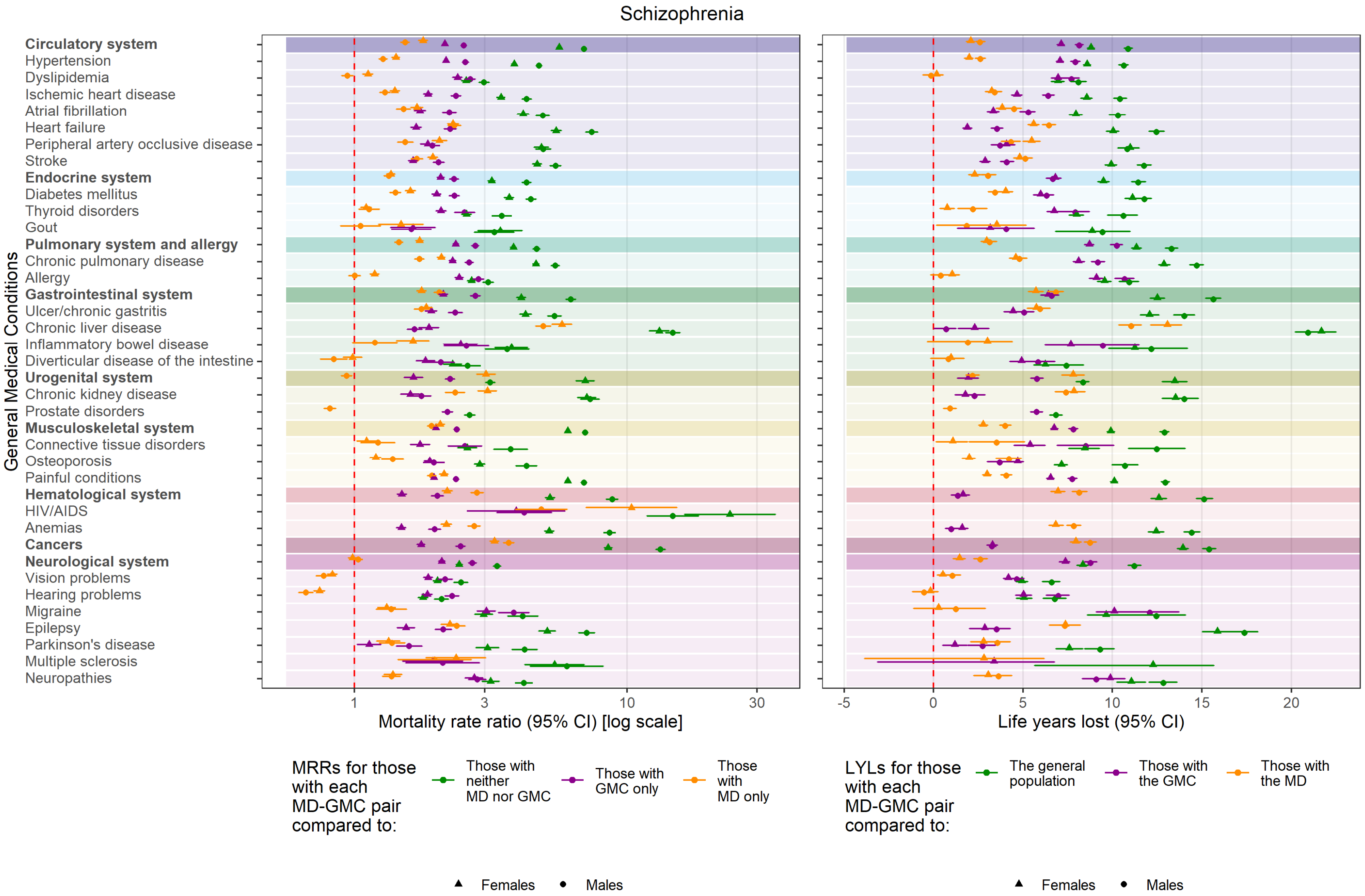

© 2022 Momen NC et al. *JAMA Psychiatry*.

eFigure 2D. Mood disorders

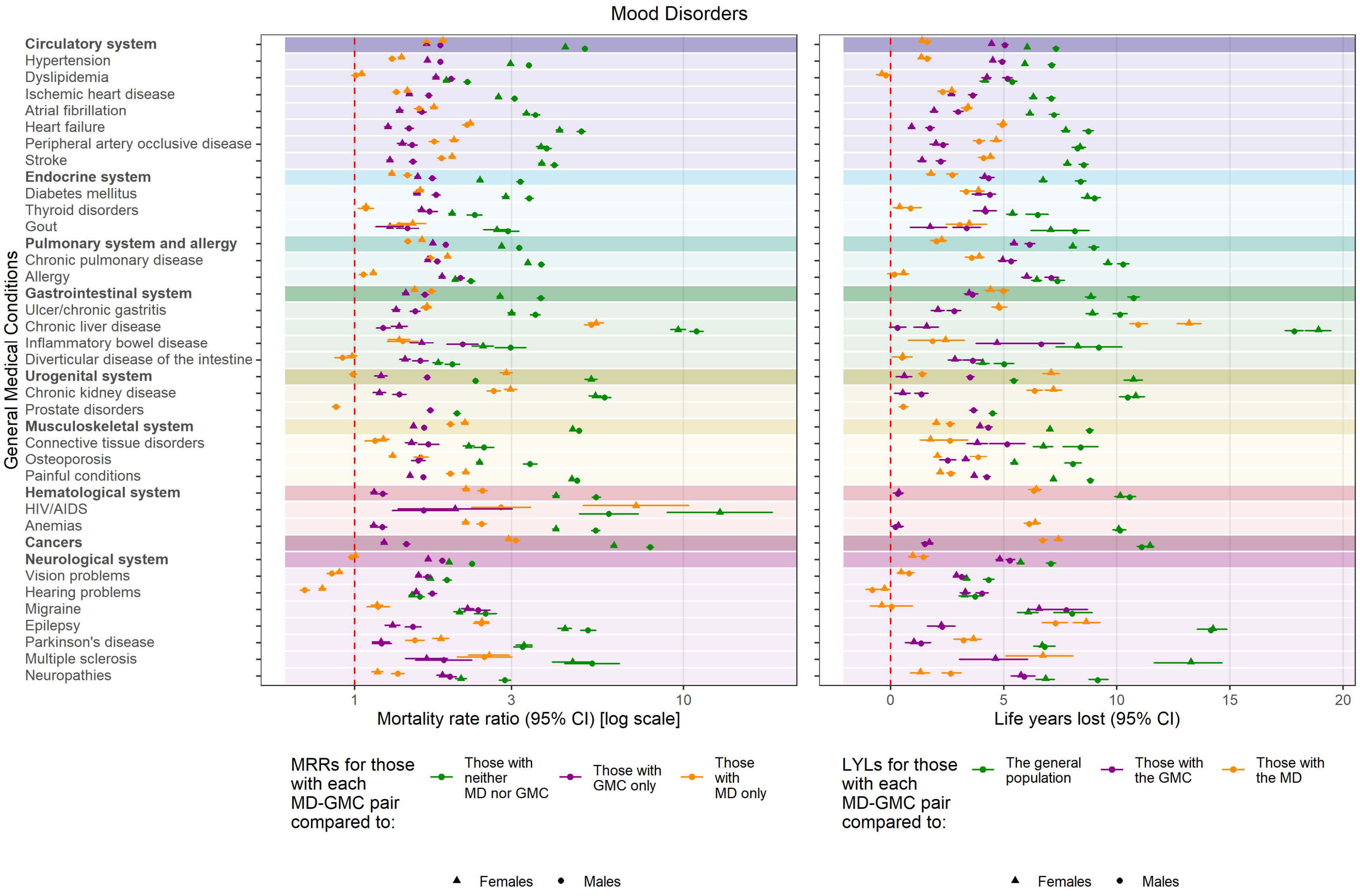

© 2022 Momen NC et al. *JAMA Psychiatry*.

eFigure 2E. Neurotic disorders

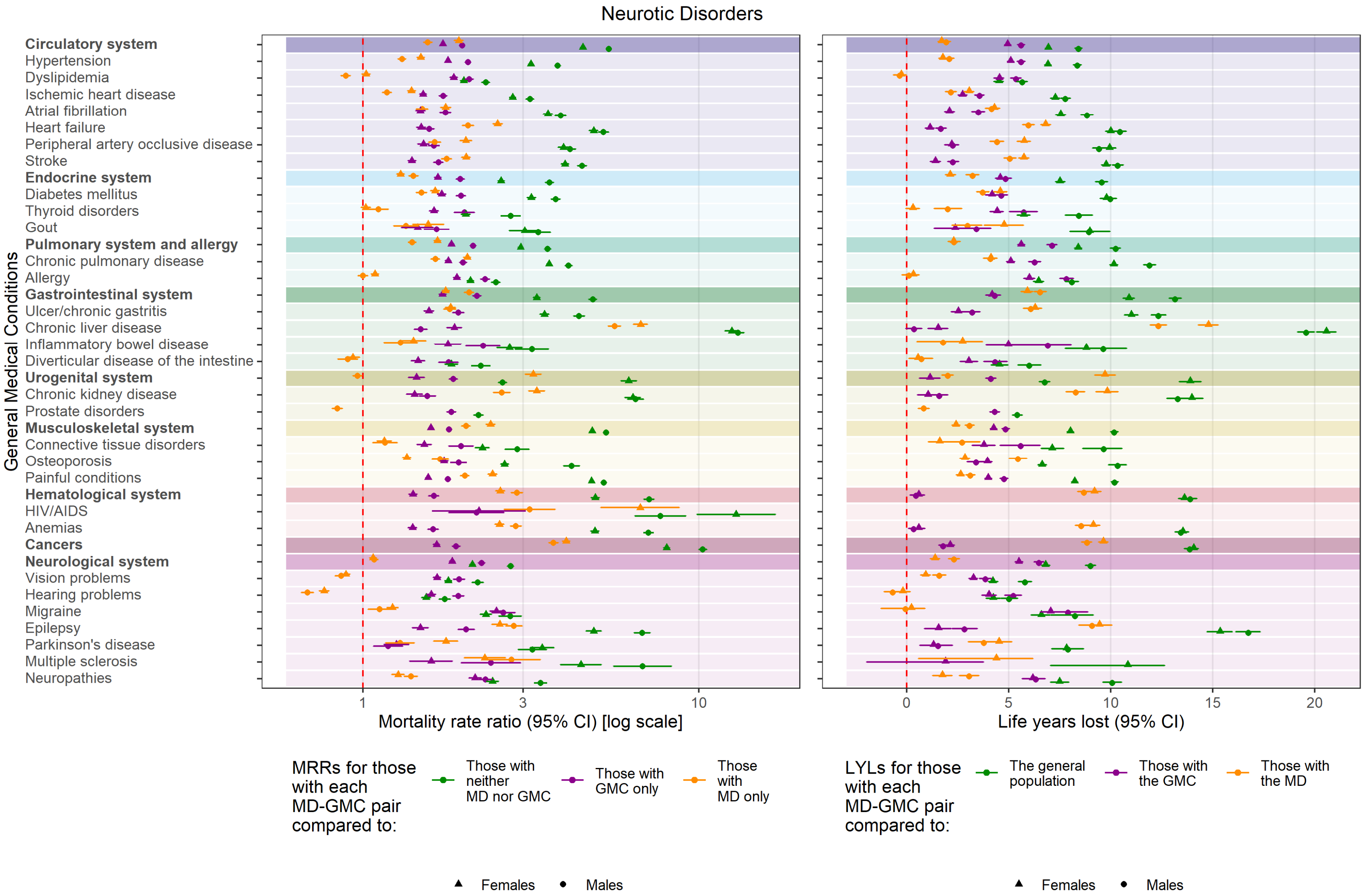

© 2022 Momen NC et al. *JAMA Psychiatry*.

eFigure 2F. Eating disorders

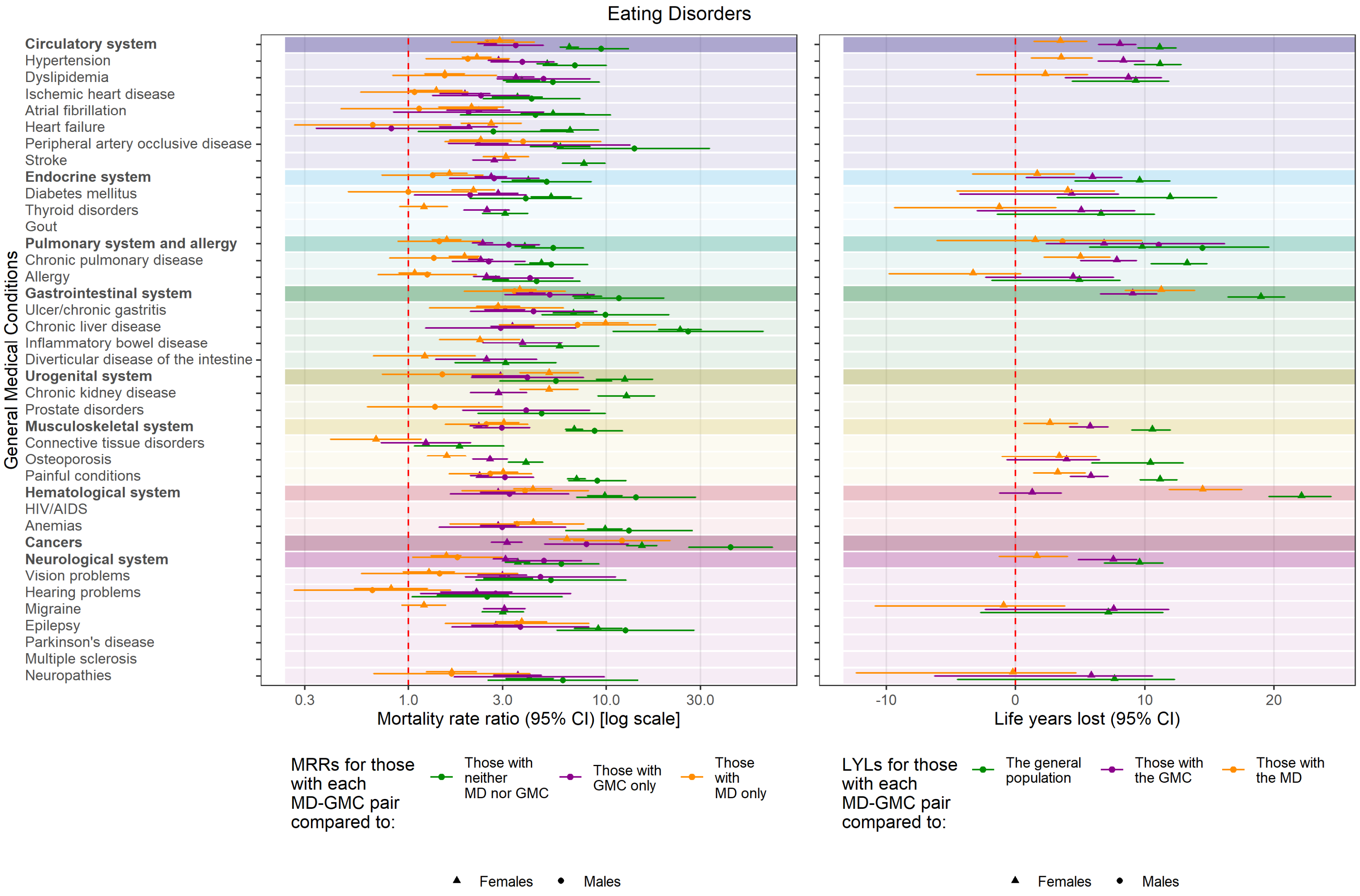

© 2022 Momen NC et al. *JAMA Psychiatry*.

eFigure 2G. Personality disorders

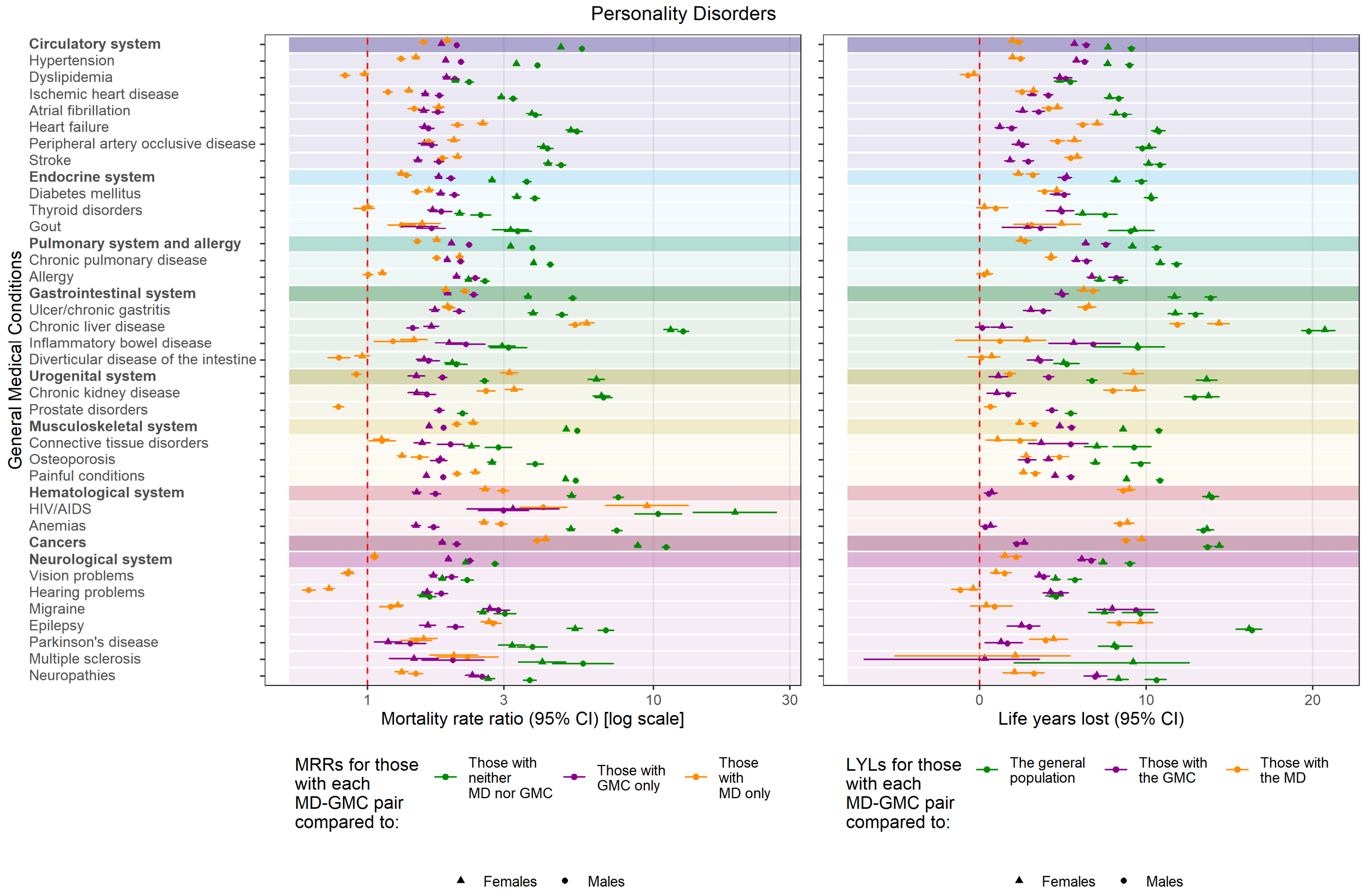

© 2022 Momen NC et al. *JAMA Psychiatry*.

eFigure 2H. Intellectual disabilities

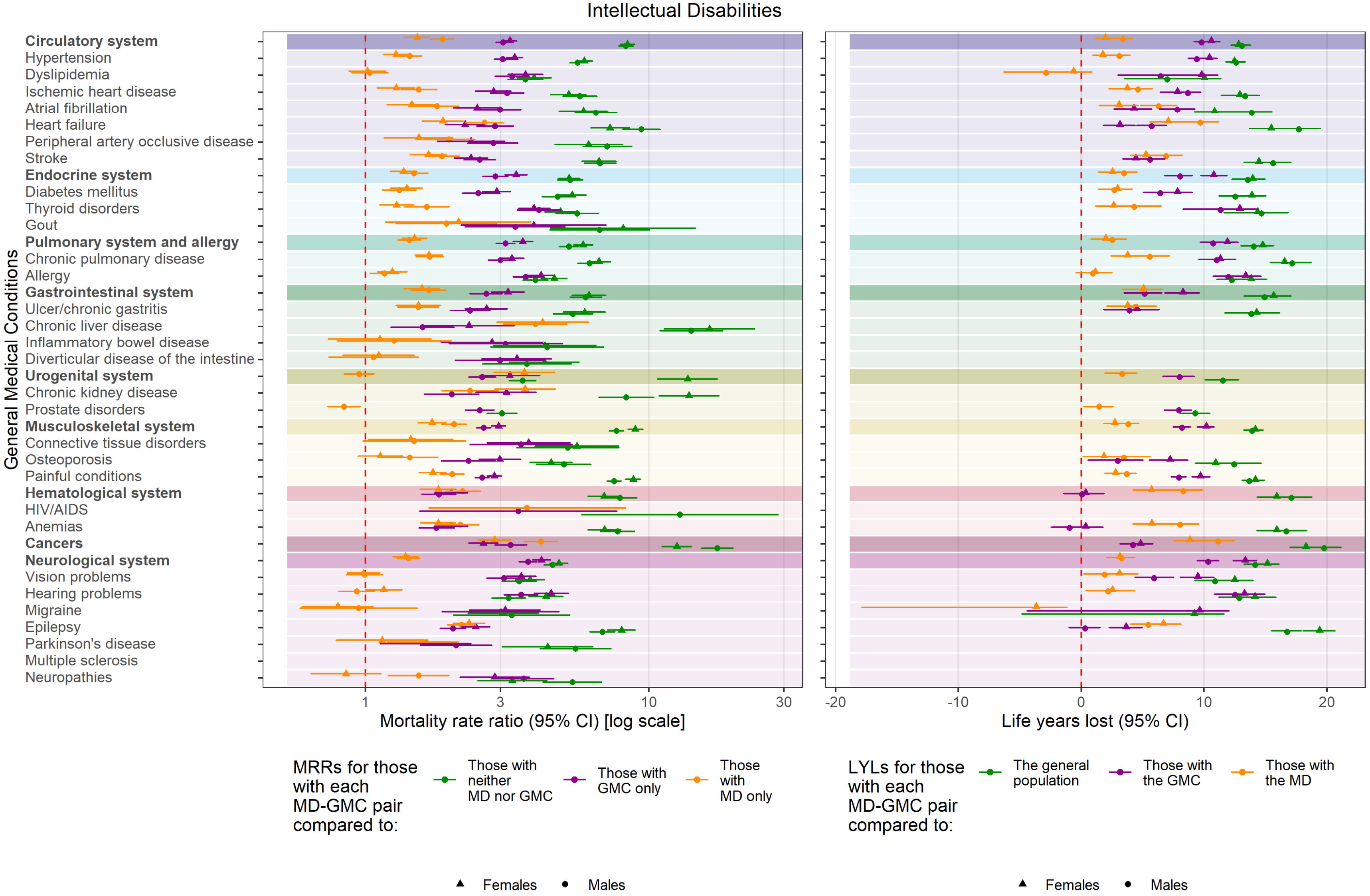

© 2022 Momen NC et al. *JAMA Psychiatry*.

eFigure 2I. Developmental disorders

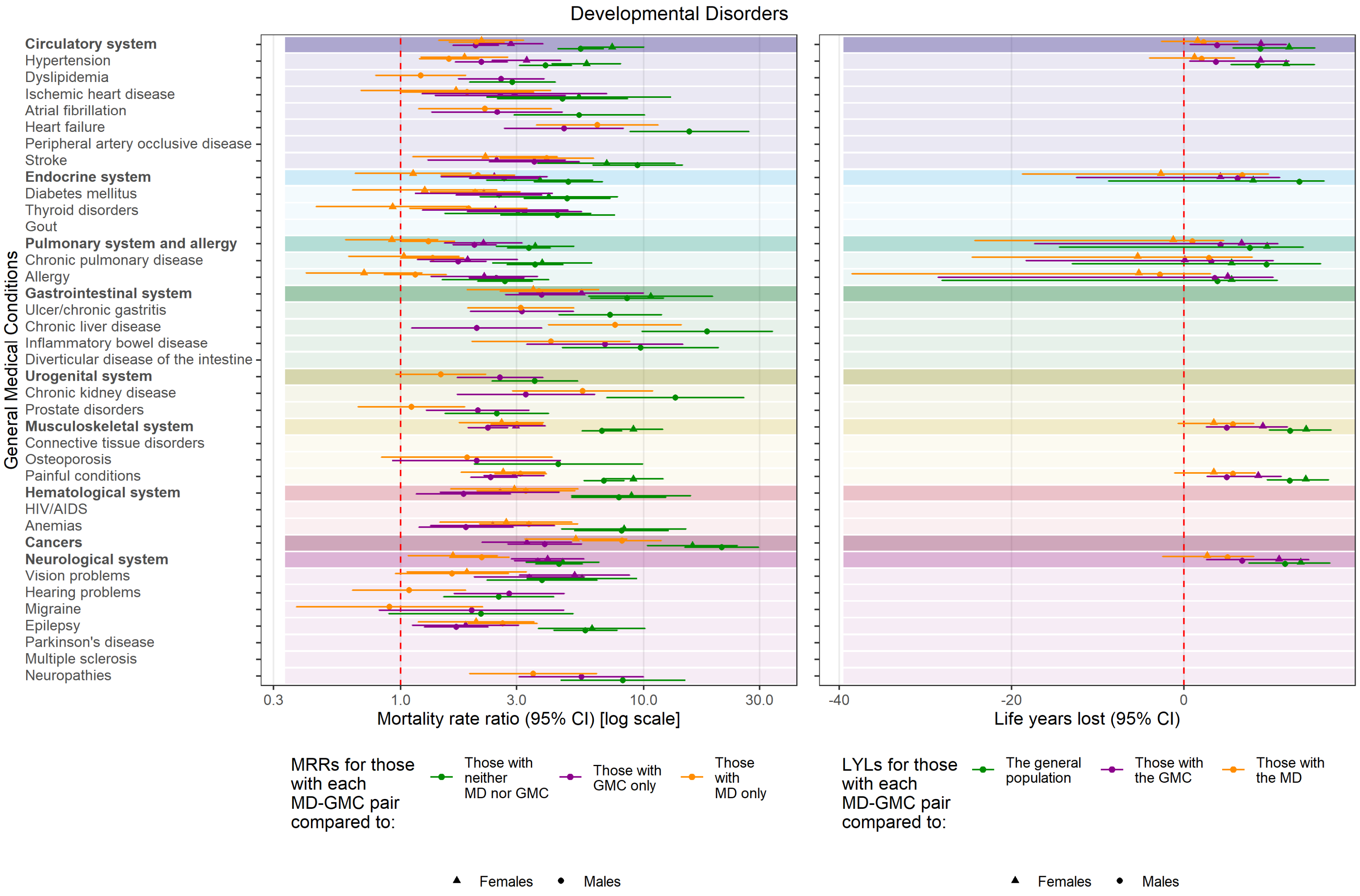

© 2022 Momen NC et al. *JAMA Psychiatry*.

eFigure 2J. Behavioral disorders

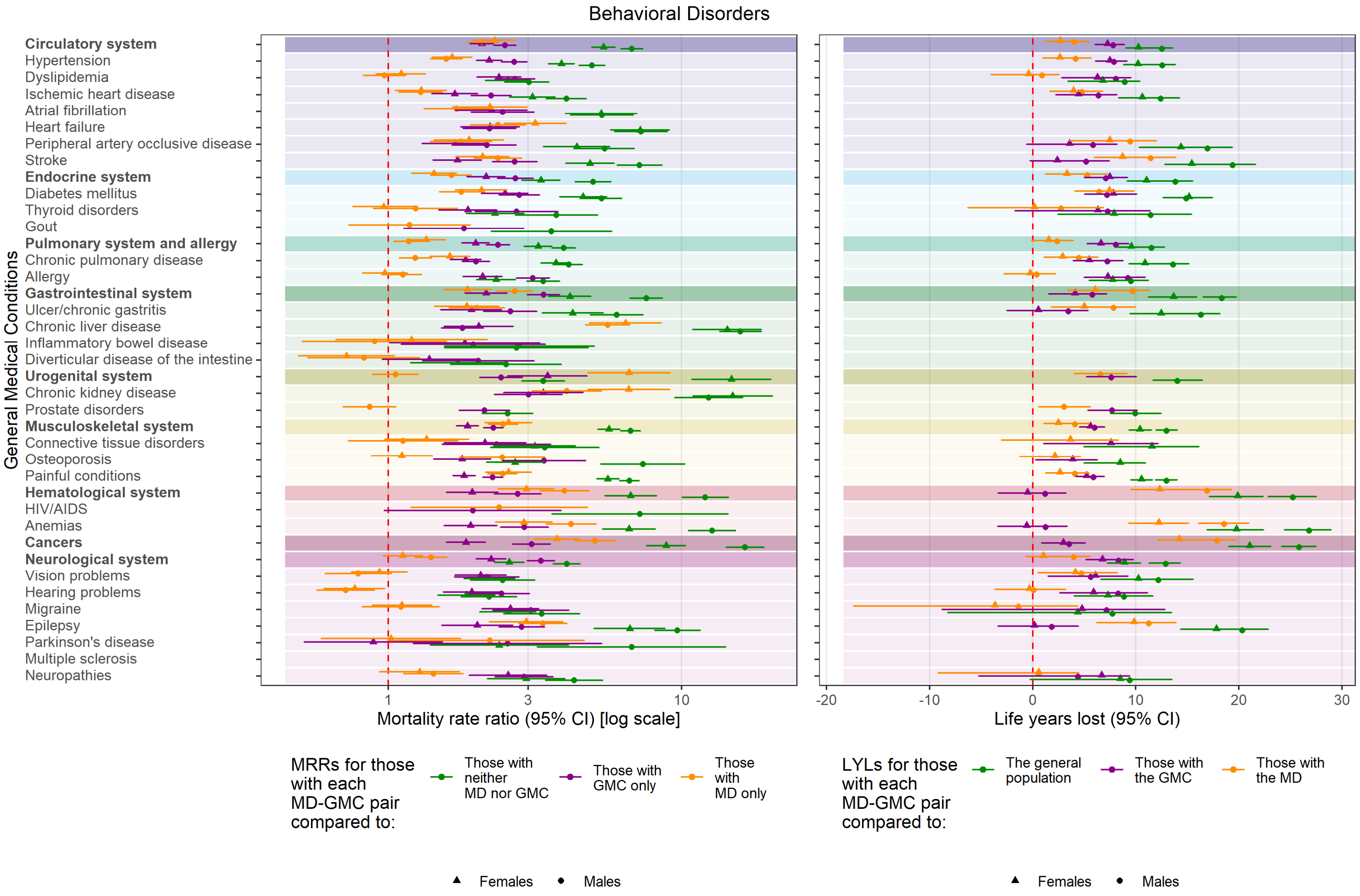

© 2022 Momen NC et al. *JAMA Psychiatry*.

eReferences

1. Mors O, Perto GP, Mortensen PB. The Danish Psychiatric Central Research Register. Scandinavian Journal of Public Health 2011;39(7 Suppl):54-7. DOI: 10.1177/1403494810395825.
2. Pedersen CB, Mors O, Bertelsen A, et al. A comprehensive nationwide study of the incidence rate and lifetime risk for treated mental disorders. JAMA Psychiatry 2014;71(5):573-81. DOI: 10.1001/jamapsychiatry.2014.16.
3. Momen NC, Plana-Ripoll O, Agerbo E, et al. Association between Mental Disorders and Subsequent Medical Conditions. N Engl J Med 2020;382(18):1721-1731. DOI: 10.1056/NEJMoa1915784.
4. Plana-Ripoll O, Pedersen CB, Holtz Y, et al. Exploring Comorbidity Within Mental Disorders Among a Danish National Population. JAMA Psychiatry 2019;76:3:259–270. DOI: 10.1001/jamapsychiatry.2018.3658.
5. Prior A, Fenger-Gron M, Larsen KK, et al. The Association Between Perceived Stress and Mortality Among People With Multimorbidity: A Prospective Population-Based Cohort Study. Am J Epidemiol 2016;184(3):199-210. DOI: 10.1093/aje/kwv324.
6. Lynge E, Sandegaard JL, Rebolj M. The Danish National Patient Register. Scandinavian Journal of Public Health 2011;39(7 Suppl):30-3. DOI: 10.1177/1403494811401482.
7. Schmidt M, Schmidt SA, Sandegaard JL, Ehrenstein V, Pedersen L, Sorensen HT. The Danish National Patient Registry: a review of content, data quality, and research potential. Clin Epidemiol 2015;7:449-90. DOI: 10.2147/CLEP.S91125.
8. Kildemoes HW, Sorensen HT, Hallas J. The Danish National Prescription Registry. Scand J Public Health 2011;39(7 Suppl):38-41. DOI: 10.1177/1403494810394717.
9. Xu R, O'Quigley J. Estimating average regression effect under non-proportional hazards. Biostatistics 2000;1(4):423-39. DOI: 10.1093/biostatistics/1.4.423.
10. Plana-Ripoll O, Musliner KL, Dalsgaard S, et al. Nature and prevalence of combinations of mental disorders and their association with excess mortality in a population-based cohort study. World Psychiatry 2020;19(3):339-349. DOI: 10.1002/wps.20802.

© 2022 Momen NC et al. *JAMA Psychiatry*.
